# Dilute electrolytes for suppressing metal anode corrosion during calendar aging and cycling in aqueous zinc batteries

**DOI:** 10.1038/s41467-026-75100-x

**Published:** 2026-08-06

**Authors:** Haoyang Wu, Bo Liu, Dingyi Zhao, Dongfang Cheng, Keyue Liang, Xintong Yuan, Kaixi Chen, Min-Ho Kim, Kaiyan Liang, Jung Tae Kim, Jiayi Yu, Tian-Yu Wang, Philippe Sautet, Yuzhang Li

**Affiliations:** 1 Department of Chemical and Biomolecular Engineering, University of California, Los Angeles, CA 90095 USA; 2 California NanoSystems Institute (CNSI), University of California, Los Angeles, CA 90095 USA; 3 Department of Chemistry and Biochemistry, University of California, Los Angeles, CA 90095 USA

**Keywords:** Batteries, Energy, Batteries

## Abstract

While numerous improvements in cycling stability have been demonstrated for next-generation battery chemistries with metallic anodes, their calendar aging (e.g., capacity loss during idle periods of rest) performance painfully lags behind and remains a critical bottleneck hindering their practical deployment. In contrast to their commercial counterparts, metallic anodes exhibit substantial capacity loss during calendar aging. Despite several recent studies exploring the underlying reasons for calendar aging, few solutions have been proposed to mitigate this key issue. Here, we design a low concentration electrolyte (0.1 M ZnSO_4_) that can reduce calendar aging losses in Zn metal chemistries by more than an order of magnitude (<1.5% capacity fade after 24 hours of aging) while still maintaining improved cycling stability (>3300 cycles at 4 C) with an average Coulombic efficiency of 99.8%. We find that solvated water molecules (rather than unsolvated water molecules) drive Zn corrosion, motivating our effort to minimize these reactive solvated water molecules through a holistic approach centered around concentration reduction, aided by isotopic solvent substitution and targeted additives. This strategy could be applicable to other battery chemistries and provides an approach that can address both calendar aging and cycling stability, both of which are necessary for practical applications.

## Abstract (web summary)

Aqueous zinc batteries lose capacity while resting, limiting their use for grid storage. Here, authors show that low Zn^2+^ concentration electrolytes suppress zinc corrosion by reducing reactive solvated water while maintaining long cycling stability.

## Introduction

**Fig. 1:**
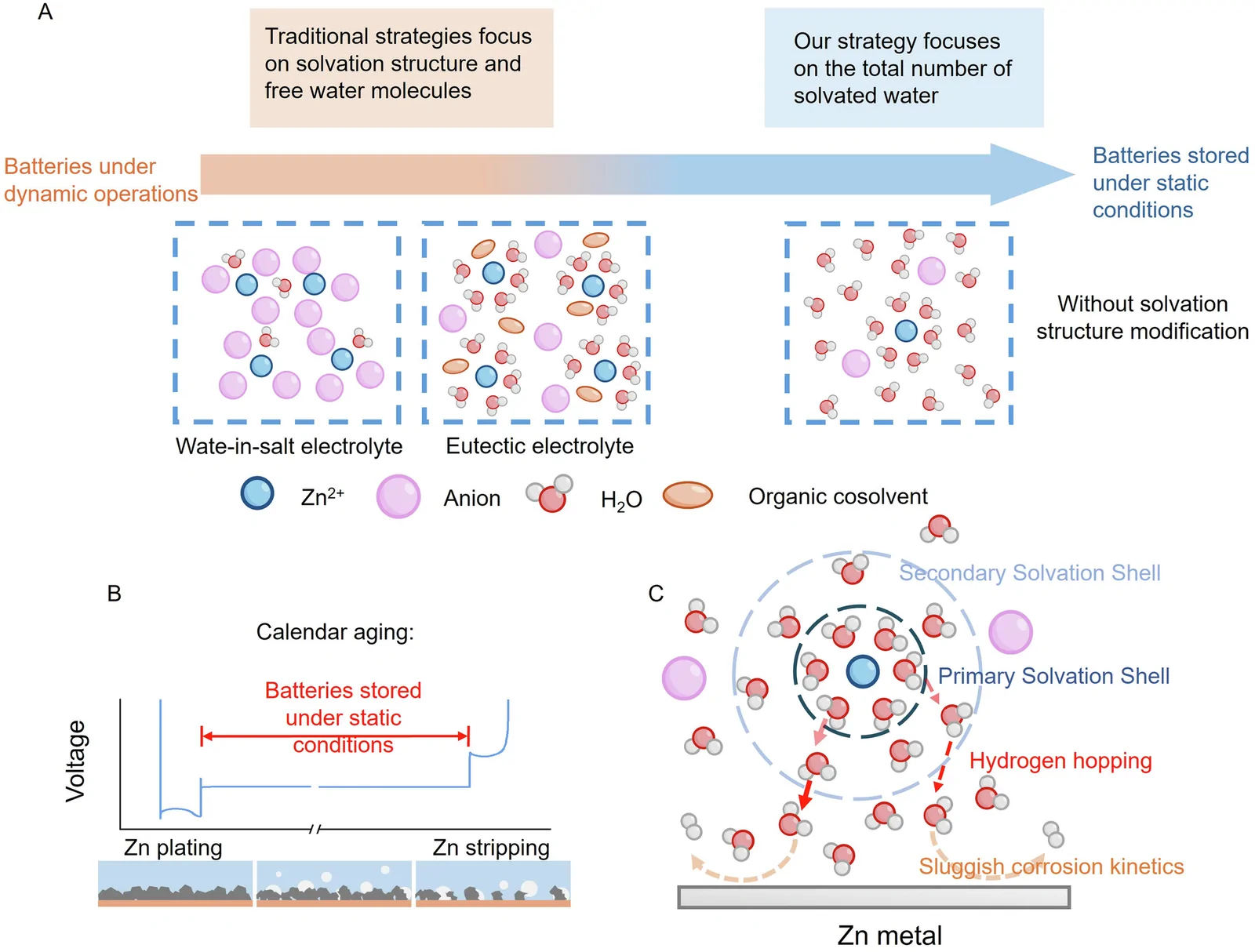
Schematics of electrolyte design principle between traditional electrolyte and our electrolyte, calendar aging process, and Zn^2+^ solvation structure. **A** Design principle on solvation structure. **B** Chemical corrosion and dead Zn formation during 24-h calendar aging lead to pronounced CE loss^8^. **C** Schematic illustration showing how a dilute electrolyte reduces Zn metal corrosion during calendar aging by minimising the amount of solvated water molecules. Source data are provided as a Source Data file.

Aqueous zinc-ion batteries (AZBs) are promising candidates for grid-scale energy storage due to their non-flammability, low cost, and low toxicity^1–3^. Recent advancements have focused on enhancing cycling performance (Fig. 1A), with neutral or mildly acidic electrolytes outperforming alkaline counterparts, achieving Coulombic efficiency (CE) above 99.9%^4,5^. However, in real-world battery applications, batteries typically spend around 80% of their operational lifetime in idle or rest conditions without active charging or discharging^6^. The stability during calendar aging—capacity loss during ideal rest periods—remains a necessary yet critically unmet need for practical application which was also proposed in lithium metal battery (Fig. 1B)^7^. Current state-of-the-art electrolytes fail to suppress zinc corrosion and hydrogen evolution reactions (HER) over extended durations, leading to the formation of dead Zn with substantial capacity losses of 30–40%^8,9^. Unlike lithium-based systems, where solid electrolyte interphases (SEI) provide passivation^10–12^, AZBs lack such protective layers, leaving Zn highly susceptible to chemical corrosion and dead Zn formation^13^. Moreover, strategies that improve cycling stability often exacerbate calendar aging^8^, highlighting a trade-off that hinders practical implementation. Enabling battery stability during both cycling and rest remains an elusive challenge that requires a deeper understanding of the factors driving corrosion during calendar aging and charge/discharge cycling.

Efforts to mitigate corrosion during cycling have focused on modifying the solvation structure of aqueous electrolytes (Fig. 1A). An important example is the water-in-salt electrolyte, which uses 1 m Zn(TFSI)_2_ and 20 m LiTFSI or 30 m ZnCl_2_ to reduce water activity by promoting the coordination of Zn^2+^ with TFSI anions^14,15^. This strategy drastically reduces the total number of water molecules available and thus the net water reactivity, leading to the apparent voltage stability window. While these approaches enhance cycling performance, their high salt concentration increases viscosity and cost, limiting their practicality. Another strategy introduces organic molecules (e.g., dimethylsulfoxide (DMSO), methanol) with high donor numbers (DN) to modify the solvation structure to strengthen the O–H bond and decrease H_2_O activity^16–18^. Although effective for improving cycling performance, these additives fail to adequately suppress capacity loss caused by chemical corrosion during calendar aging^19^, raising two critical questions: will free water outside the solvation shell contribute to Zn corrosion, and is there an effective way to reduce the concentration of solvated water molecules? Insights from fuel cells, where pure water electrolysis exhibits a high overpotential for HER^20^, suggest that free water in the bulk electrolyte may not substantially drive HER. Instead, water molecules in the solvation shell—categorized as primary (strongly bound to cations) and secondary (dipole-oriented toward the central ion)^21^—play a decisive role in corrosion mechanisms (Fig. 1C). Understanding these molecular interactions could guide the design of cost-effective electrolytes that simultaneously enhance cycling stability and mitigate calendar aging, advancing AZBs for practical applications.

Here, we present a dilute ZnSO_4_-based electrolyte (defined here as ~10× lower Zn-salt concentration than conventional aqueous Zn electrolytes (0.5-3 M)) engineered to address the challenge of maintaining calendar aging stability without compromising cycling stability. By systematically reducing the presence of highly reactive solvated water, identified as the main driver of Zn corrosion, we achieve a near-neutral pH environment supplemented by isotopic substitution to further decrease electrolyte reactivity, as well as carefully selected additives. Specifically, Li_2_SO_4_ decreases ionic transport resistance, while N, N-dimethylformamide (HCON(CH_3_)_2_, DMF) promotes the in situ formation of an SEI, thereby protecting the Zn anode from chemical corrosion during prolonged idle periods and extended cycling. As a result, this electrolyte retains 98.5% of its capacity after 24 h of aging, a substantial improvement compared to 63% retention with a 2 M ZnSO_4_ electrolyte across various conditions. Moreover, it delivers distinct cycling performance, with an average CE of 99.8% over 3300 cycles in Zn | |LiMn_2_O_4_ full-cells. By simultaneously mitigating capacity loss during calendar aging and enhancing cycling efficiency, our strategy offers a promising route toward practical, high-performance AZBs for large-scale energy storage and beyond.

## Results

### Understanding the reactivities of solvated water and free water

**Fig. 2:**
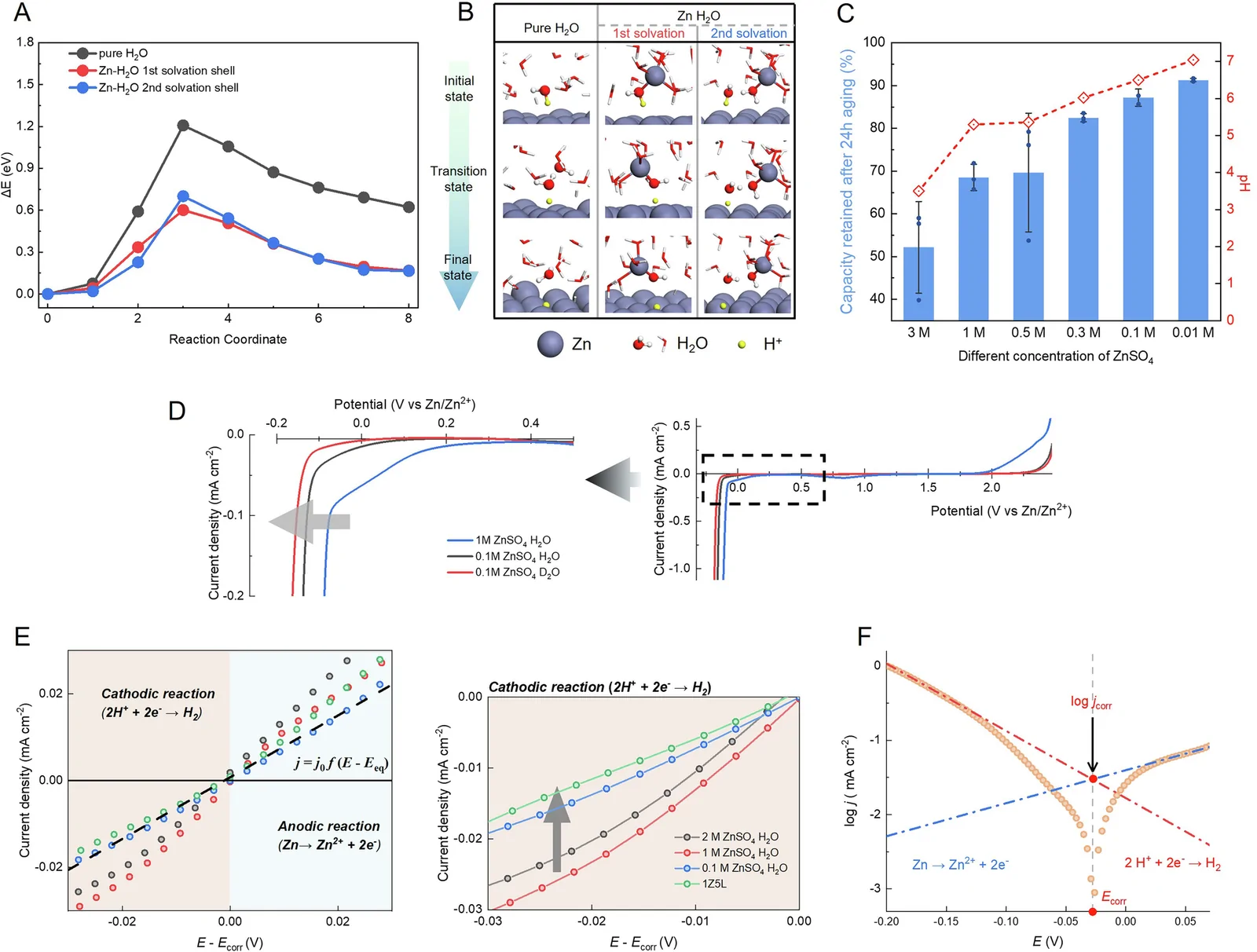
Reducing salt concentration and substituting D_2_O to decrease water reactivity. **A** Reaction profile of proton-donating process at −0.8 V vs SHE with different proton sources and reaction pathways. **B** Atomic structures of initial state, transition state, and final state with different H_2_O source. **C** Relationship between capacity retention after 24 hours of aging and various ZnSO_4_ concentrations using H_2_O as solvent, along with the corresponding pH values (the batteries were tested at a capacity of 1 mAh cm^−2^ and a current of 1 mA cm^−2^ at 25 °C, with a cutoff voltage of 0.5 V). **D** Overall electrochemical stability windows of the electrolytes at 0.1 M ZnSO_4_ with and without D_2_O, including magnified views of the cathodic regions, at a scan rate of 1 mV s^−1^. **E** Representative LSV data. The dashed line indicates the fitted response of the dilute electrolyte at equilibrium potential (E = E_eq_), including magnified views of the cathodic reaction. **F** Tafel plot of dilute electrolyte derived by LSV measurements. Error bars represent the standard deviation of 3 to 4 independent measurements. Source data are provided as a Source Data file.

Distinguishing the reactivities between solvated and free water is critical for elucidating Zn corrosion mechanisms in aqueous electrolytes. Crystal Orbital Hamilton Population (COHP) analysis (Supplementary Note 1) reveals that O-H bonds in Zn-coordinated solvated water have diminished bonding contributions and a pronounced anti-bonding feature near the Fermi level (Supplementary Fig. 1), leading to a weaker O–H bond in solvated water that is more susceptible to cleavage and stronger proton-donating ability compared to free water. This increased lability facilitates proton transfer and promotes hydrogen evolution reaction (HER), identifying solvated water as the primary driver of the acidic environment responsible for Zn corrosion. To quantify the proton donation ability, we sampled the minimum-energy path to calculate the reaction barrier for proton donation, using the Volmer step as our example since it is widely considered the rate-determining step in the hydrogen evolution reaction (HER)^22,23^. In pure water, the Volmer step in proton donation exhibits a high reaction barrier (~1.2 eV) and overall energy (~0.6 eV), largely due to the destabilization of the hydroxide ion in solution^24^. When Zn is present in solvated water, both proton donation from the 1st shell solvated water directly and 2nd shell solvated water via a Grotthuss-like mechanism benefit from strong Zn–OH interactions, reducing the reaction barrier and reaction energy by ~0.6 eV and ~0.4 eV, respectively. (Fig. 2A and Supplementary Note 2). Overall, Zn coordination in solvated water substantially weakens the O–H bond and enhances the HER by stabilizing the transition and final states compared to free water (Fig. 2B).

### Reducing salt concentration to decrease solvated water reactivity

To mitigate these effects, we propose reducing the proportion of highly reactive solvated water by decreasing the Zn salt concentration. To minimize corrosion during long-term aging, we intentionally avoid inducing (electro)chemical reactions that could form SEI layers through salt composition. Conventional salts such as Zn(TFSI)_2_ and Zn(OTF)_2_ contain highly reactive anions (e.g., -CF_3_ groups) that tend to decompose, producing SEI films. Although such decomposition can provide passivation benefits, it typically proceeds at the cost of parasitic Zn consumption, making these salts unsuitable for long-term aging conditions^18^. Thus, we did a systematic study of ZnSO_4_-based electrolytes at various concentrations, showing a pH increase from 3.4 at 3 M ZnSO_4_ to 6.5 at 0.1 M ZnSO_4_, eventually reaching neutrality (pH = 7) at 0.01 M ZnSO_4_ (Fig. 2C), supported by a decreased H-band absorbance peak detected at ~3414 cm^-1^ in Fourier transform infrared (FTIR) spectra (Supplementary Fig. 2)^25–27^. More importantly, this pH rise correlates with substantially improved capacity retention after 24 hours of calendar aging, with 87.2% at 0.1 M and 91.3% at 0.01 M, compared to 52.2% at 3 M ZnSO_4_ and 65.3% at 2 M ZnSO_4_ as previously reported (Fig. 2C)^9^. Specifically, this result contrasts with earlier strategies aimed at reducing water reactivity, which have largely focused on highly concentrated electrolytes to strengthen Zn^2+^-anions coordination^14^. Another common strategy is cosolvent design by organic molecules with high donor number (DN) to replace H_2_O in Zn^2+^ primary solvation shell and break H-bond network in bulk electrolyte^28^. However, even partial replacement of H_2_O with typical organic solvents to modify the Zn²⁺ solvation structure fails to maintain over 90% Zn capacity retention after 24 hours of aging (Supplementary Fig. 3). In addition, dilute electrolytes are substantially lighter and cheaper than previous high-concentration and water-in-salt formulations, which could also facilitate a higher overall energy density in the full-cell. Although very dilute electrolytes (e.g., 0.01 M) are impractical for real-world batteries due to limited ion transport and a higher risk of dendrite formation^29,30^, a 0.1 M ZnSO_4_ solution with a near-neutral pH of 6.5 provides a viable balance between corrosion mitigation (87.2% capacity retention after 24 hours of calendar aging) and practical feasibility in the subsequent experiments.

### Solvent design to further decrease water reactivity

When aiming to control corrosion reactions in electrolyte engineering, solvent design is also important. Although water is traditionally used in AZBs due to its high ionic conductivity, its narrow electrochemical stability window (1.23 V for pure water) severely limits operation, primarily because of HER and oxygen evolution reactions (OER)^31^. To expand the operating voltage range and further mitigate corrosion, we introduce deuterium oxide (D_2_O, also known as heavy water) into the 0.1 M electrolyte. Through the kinetic isotope effect, O–D bonds have higher binding energies than O–H bonds^32^, making deuterium less prone to protonation and reducing the overall reactivity of water—particularly the solvated water molecules that directly drive corrosion. Lower proton availability helps suppress HER and OER, thereby extending both the cathodic and anodic stability limits of the electrolyte^9,33^. As shown in Fig. 2D, in cyclic voltammetry (CV) tests, D_2_O substitution (0.1 M ZnSO_4_ in D_2_O) effectively broadens the electrochemical window, underscoring its potential to increase the energy density of AZBs while simultaneously inhibiting corrosion.

To further evaluate the corrosion behavior, we performed Tafel analysis (Fig. 2E, F, Supplementary Table 2). The corrosion current density (j_corr_), a key descriptor of interfacial charge-transfer kinetics^34^, increases with ZnSO_4_ concentration, reflecting more facile Zn redox reactions in concentrated electrolytes. In contrast, our dilute electrolyte exhibits a lower j_0_ at negative overpotentials (E - E_eq_ < 0), indicating suppressed spontaneous Zn dissolution and hydrogen evolution. This trend aligns with our goal of minimizing parasitic reactions by decreasing the number of solvated water molecules. As shown in Fig. 2F and Supplementary Fig. 4A, B, decreased j_corr_ with D_2_O substitution strengthens our conclusion that deuterated systems, particularly when dilute, substantially mitigate electrolyte-induced corrosion. In addition, D_2_O-based electrolytes display a more negative corrosion potential (E_corr_), indicating that Zn corrosion is thermodynamically unfavorable in these systems (Supplementary Fig. 4C). In particular, pure H_2_O and D_2_O show low j_corr_ values and negative E_corr_ (Supplementary Fig. 4A–C), suggesting that free water molecules alone are less aggressive to Zn compared to solvated water. Although the addition of supporting salts slightly increases electrolyte reactivity, the dilute electrolyte still shows a much lower j_corr_ compared to conventional water-based electrolytes, further demonstrating its low corrosion behavior (Supplementary Fig. 4A).

### Ionic transport elevation and conformal SEI formation

Although the 0.1 M ZnSO_4_-D_2_O electrolyte effectively mitigates corrosion, its low Zn^2+^ concentration and the use of D_2_O markedly reduce ionic conductivity, potentially hindering long-term cycling stability and overall cell performance (Supplementary Fig. 5). To resolve this issue, 0.5 M Li_2_SO_4_ was added into the electrolyte as supporting salt without increasing Zn metal corrosion rate. Unlike divalent Zn^2+^, the low ionic potential Li^+^ exerts a substantially weaker effect on the electron density of coordinated water molecules^35^, cutting the impact on solvation roughly in half compared to a similarly sized divalent cation confirmed by different cation-water complex free energy (Supplementary Table 1 and Supplementary Fig. 6A)^36^. Moreover, Li^+^ (ionic radius: 90 pm) maintains a shorter internuclear distance to the oxygen atom in water than Zn^2+^ (ionic radius: 88 pm), further diminishing its influence on electron density distribution and the resulting solvation structure^14^. As shown in Supplementary Fig. 7, adding Li_2_SO_4_ substantially decreases the overall ionic transport resistance, offsetting the drawbacks of using a dilute Zn^2+^ without specifically altering the reactivity of solvated water in the electrolyte.

**Fig. 3:**
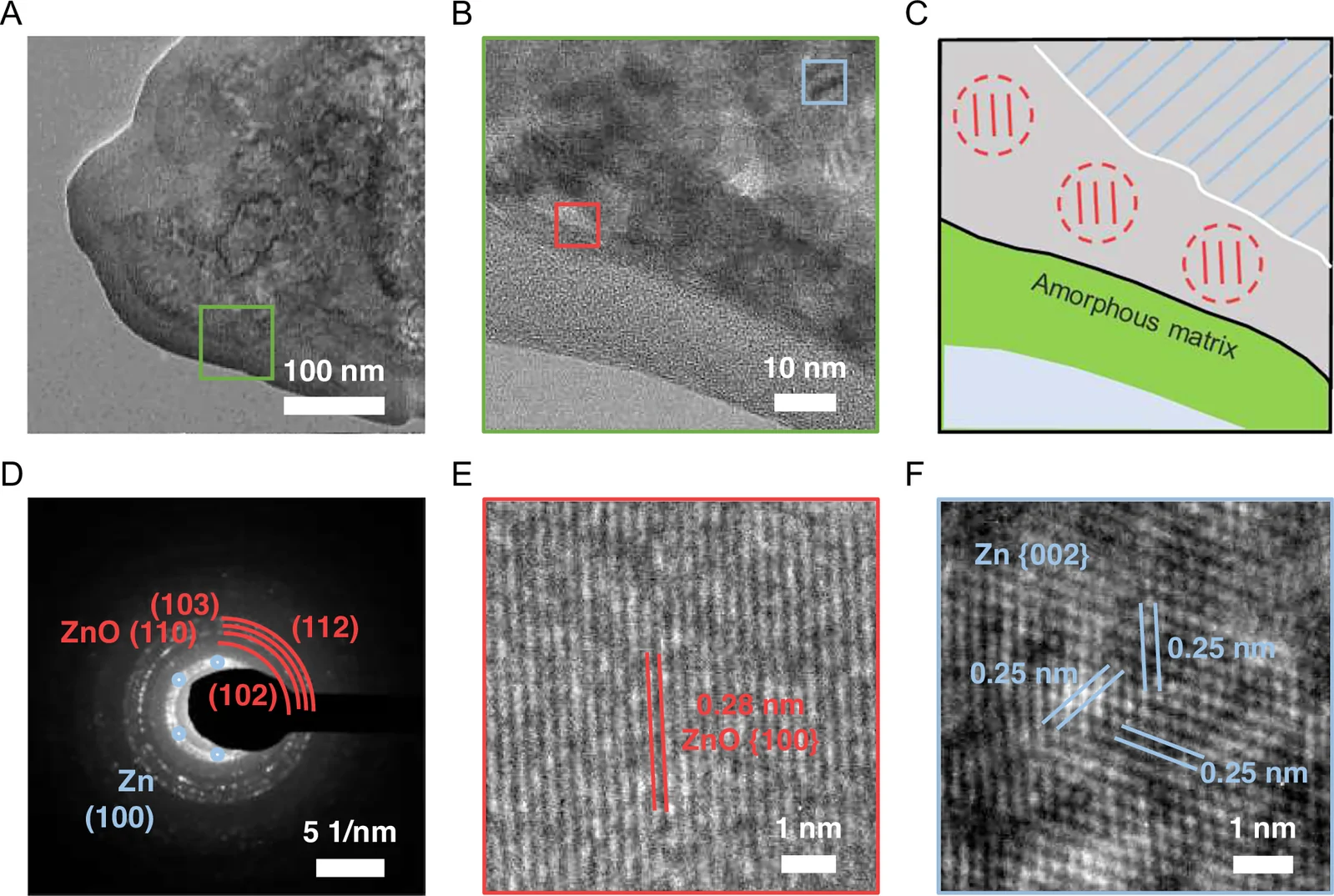
Incorporating Li_2_SO_4_ and DMF to facilitate stable SEI formation. **A**, **B** TEM images of deposited Zn metal with a conformal SEI layer on the surface. **C** Schematic depiction of the amorphous SEI layer encapsulating the metallic Zn. **D** SAED pattern revealing ZnO and Zn. Lattice spacings of **E** ZnO and **F** Zn along the (002) direction. TEM samples were prepared by drop-casting after the half-cell had been cycled for 3 cycles at an areal capacity of 1 mAh cm^−2^ and a current density 1 mA cm^−2^.

However, this modification alone is insufficient to maintain a robust, long-lasting electrode/electrolyte interface with strong anti-corrosion properties for long-term cycling. A sacrificial additive is necessary to regulate Zn deposition morphology. This additive should be aprotic, with a relatively high donor number (DN) and high dielectric constant, to exhibit Zn salt affinity comparable to that of water (Supplementary Fig. 6B). To fulfill this role, we selected low concentration, 5 vol%, DMF as an effective choice to promote in situ SEI formation guided by earlier studies^37^, where DMF acts as an additive to make a compact Zn morphology (Supplementary Figs. 9 and 10). Especially, the SEI is essentially a form of corrosion and does not necessarily prevent direct contact between water and Zn metal. Therefore, the concentration of DMF is intentionally kept low to minimize its decomposition and associated side reactions. Transmission electron microscopy (TEM) images (Fig. 3A, B and Supplementary Fig. 11A) reveal a ~ 15 nm conformal SEI on the Zn metal surface after 3 plating/stripping cycles, exhibiting a characteristic core-shell structure in which the crystalline core is surrounded by an amorphous shell (Fig. 3B, C and Supplementary Fig. 11A). Selected area electron diffraction (SAED, Fig. 3D, Supplementary Fig. 12, and Table 3) and high-resolution TEM (HRTEM) analyses indicate the coexistence of ZnO (Fig. 3E and Supplementary Fig. 11E–H) and metallic Zn (Fig. 3F and Supplementary Fig. 11B–D). The presence of ZnO as a by-product suggests that some corrosion inevitably occurs during the initial cycles before the SEI fully develops. Elemental mappings (Supplementary Fig. 13) further indicate that the SEI is composed primarily of C, O, and Zn, along with small amounts of S. X-ray Photoelectron Spectroscopy (XPS) reveals a carbonate-associated C 1 *s* component consistent with carbonate species formation at 289.6 eV^38,39^, supporting the presence of ZnCO_3_ products (Supplementary Fig. 14A). We also detect an N-H bonding at 402.0 eV in the N 1 *s* spectrum^40,41^, which is consistent with protonated amine-type species and supports the plausibility of DMF-derived nitrogenous products (i.e., H_2_N(CH_3_)_2_^+^) at the interface (Supplementary Fig. 14B). Therefore, we hypothesize that DMF (HCON(CH_3_)_2_) reacts initially with the Zn to form ZnCO_3_ and ZnO on the electrode surface, along with the involvement of dissolved oxygen present in the electrolyte^22,36^. The possible reactions are as follows:

$${{\rm{Zn}}}\,+\,{{{\rm{Zn}}}}^{2+}+\,{{{\rm{OH}}}}^{-}+\,{{\rm{HCON}}}{({{{\rm{CH}}}}_{3})}_{2}{+{{\rm{O}}}}_{2}\to {{{\rm{ZnCO}}}}_{3}{+{{\rm{ZnO}}}+{{\rm{H}}}}_{2}{{\rm{N}}}{{({{{\rm{CH}}}}_{3})}_{2}}^{+}$$

This nanostructured SEI, rich in ZnCO_3_ and ZnO, enables efficient cation transport while regulating Zn deposition by minimizing uncontrolled side reactions. Consequently, by incorporating both Li_2_SO_4_ and DMF to decrease ionic transport resistance and facilitate stable SEI formation, we get a cost-effective, well-engineered electrolyte with the formula 0.1 M ZnSO_4_ + 0.5 M Li_2_SO_4_ + 5 vol% DMF in D_2_O (1Z5L). This formulation substantially decreases the solvated water reactivity, improves ionic transport, and offers sustained anti-corrosion characteristics (Supplementary Fig. 15). Importantly, this strategy is not limited to a single electrolyte composition but instead reflects a generalizable design principle: suppressing solvated water reactivity through dilution and weakly coordinating co-solvents, combined with enhanced ionic transport and SEI formation. This general framework distinguishes our work from prior studies that focus on fixed electrolyte recipes, underscoring the potential of rational electrolyte design for corrosion-resistant aqueous Zn battery technologies. We anticipate promising electrochemical performance under both long-term calendar aging and cycling conditions with this generalizable design principle, marking a substantial step toward the practical deployment of Zn-based battery chemistries.

### Long-term calendar aging and cycling performance analysis

**Fig. 4:**
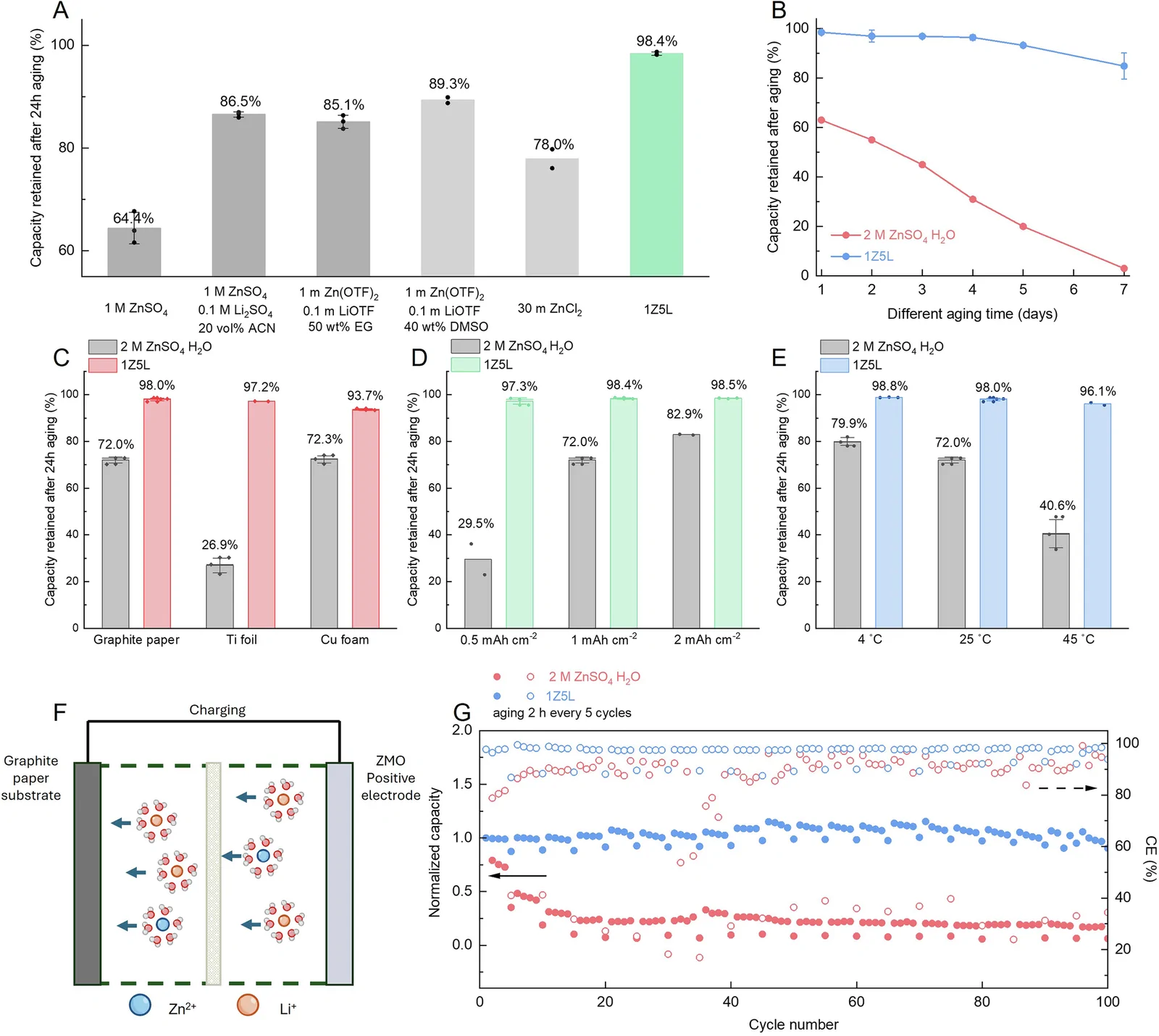
Comprehensive analysis of calendar aging performance. **A** Comparison of capacity retention after 24 hours of storage with state-of-the-art electrolytes reported in the literature. **B** Capacity retention over various storage times. **C** Capacity retention on different substrates (with a Zn deposition capacity of 1 mAh cm^−2^ at 25 °C at 1 mA cm^−2^). **D** Capacity retention under varying Zn deposition capacities (at 25 °C at 1 mA cm^−2^, using graphite paper as substrate). **E** Capacity retention at different temperatures (with a Zn deposition capacity of 1 mAh cm^−2^ at 1 mA cm^−2^, using graphite paper as substrate). All results are benchmarked against a reference sample. **F** Schematic illustration of a Zn anode-free cell using ZMO positive electrode (cathode) and graphite paper as the negative electrode (anode) substrate. **G** Anode-free full-cell performance under a hybrid cycling-and-aging protocol (aging 2 h every 5 cycles) at 0.5 mA cm^−2^ and 25 °C. Half-cells in (**A**, **B**) were tested at 25 °C with 1 mAh cm^−2^ deposited Zn metal at 1 mA cm^−2^, and the cutoff voltage was set at 0.5 V. Error bars on the figures show the standard deviation of 3–5 independent measurements. Source data are provided as a Source Data file.

Compared to commonly used and state-of-the-art electrolytes reported in the literature^22,36^, our low concentration electrolyte with low water reactivity demonstrates competitive calendar aging performance, retaining 98.4% of its capacity after 24 hours, an order-of-magnitude reduction in capacity loss (1.5% vs. 35.6% for 1 M ZnSO_4_) (Fig. 4A)^19^. To quantify the contribution of dead Zn and chemical corrosion using a low-concentration electrolyte, we applied titration gas chromatography (TGC) and found that continuous cycling led to 0.5% irreversible Zn formation. After calendar aging, 1.5% irreversible Zn was observed, comprising 0.65% electrically isolated Zn (Zn^0^) and 0.85% corrosion-derived Zn^2+^ (Supplementary Fig. 16). We further investigated the influence of aging duration, substrate type, deposition capacity, and aging temperature, all of which are known to affect aging performance^9^. The electrolyte maintains over 90% capacity retention after 5 days of storage (Fig. 4B). Even after 7 days, the cell retains 84.4% of its capacity with a stable electrolyte reactivity, which is far exceeding the 3% observed in the 2 M ZnSO_4_–H_2_O control electrolyte (Fig. 4B). Because three-dimensional (3D) hosts often outperform 2D substrates in mitigating the formation of dead Zn^8^, we evaluate both substrate types. The engineered electrolyte shows low corrosivity on both 2D (Ti foil) and 3D (graphite paper, Cu foam) substrates, achieving 97.2%, 98.0%, and 93.7% capacity retention, respectively, after 24 h (Fig. 4C). These results indicate that the chemical corrosion responsible for inactive Zn formation is largely suppressed with our engineered electrolyte. Furthermore, the aging performance maintains promising across different Zn deposition capacities with 97.3%, 98.4%, and 98.5% retention at 0.5, 1, and 2 mAh cm^-2^, respectively (Fig. 4D and Supplementary Fig. 17), implying that a larger electrode/electrolyte interface does not markedly compromise corrosion resistance. At higher temperatures (45 °C), where water reactivity typically increases^42^, the electrolyte still retains 96.1% of its capacity after 24 h, which is comparable to that at 25 °C (Fig. 4E). Under lower-temperature conditions (0 and 4 °C), the Zn anode demonstrates reversible capacities of 97.3% and 98.8% with only 2.7% and 1.2% capacity loss during calendar aging, respectively, demonstrating that the electrolyte system remains effective for suppressing aging even under near-freezing conditions (Fig. 4E and Supplementary Fig. 18). To assess performance in a more practical setting, we paired a zinc-rich ZnMn_2_O_4_ (ZMO) cathode (Supplementary Fig. 19 and 20 A) with a graphite paper current collector as an anode-free-cell at 100% depth of discharge (Fig. 4F)^43^. Under a hybrid protocol of aging (2-hour rest at 100% state of charge) after every five cycles, our dilute electrolyte substantially mitigates capacity loss, delivering an average CE of 96.3% and capacity retention of 90.4% over 100 cycles at 0.5 mA cm^-2^ (Fig. 4G and Supplementary Fig. 21). In contrast, the 2 M ZnSO_4_–H_2_O control electrolyte only has 76.8% average Coulombic efficiency and 0.1% capacity retention under the same conditions (Fig. 4G and Supplementary Fig. 22). Taken together, the versatility across different substrates, deposition capacities, and temperatures highlights the strong anti-corrosion and aging mitigation capabilities of our electrolyte in Zn battery chemistries, suggesting that it could be a promising candidate for broader implementation without sacrificing cycling performance.

**Fig. 5:**
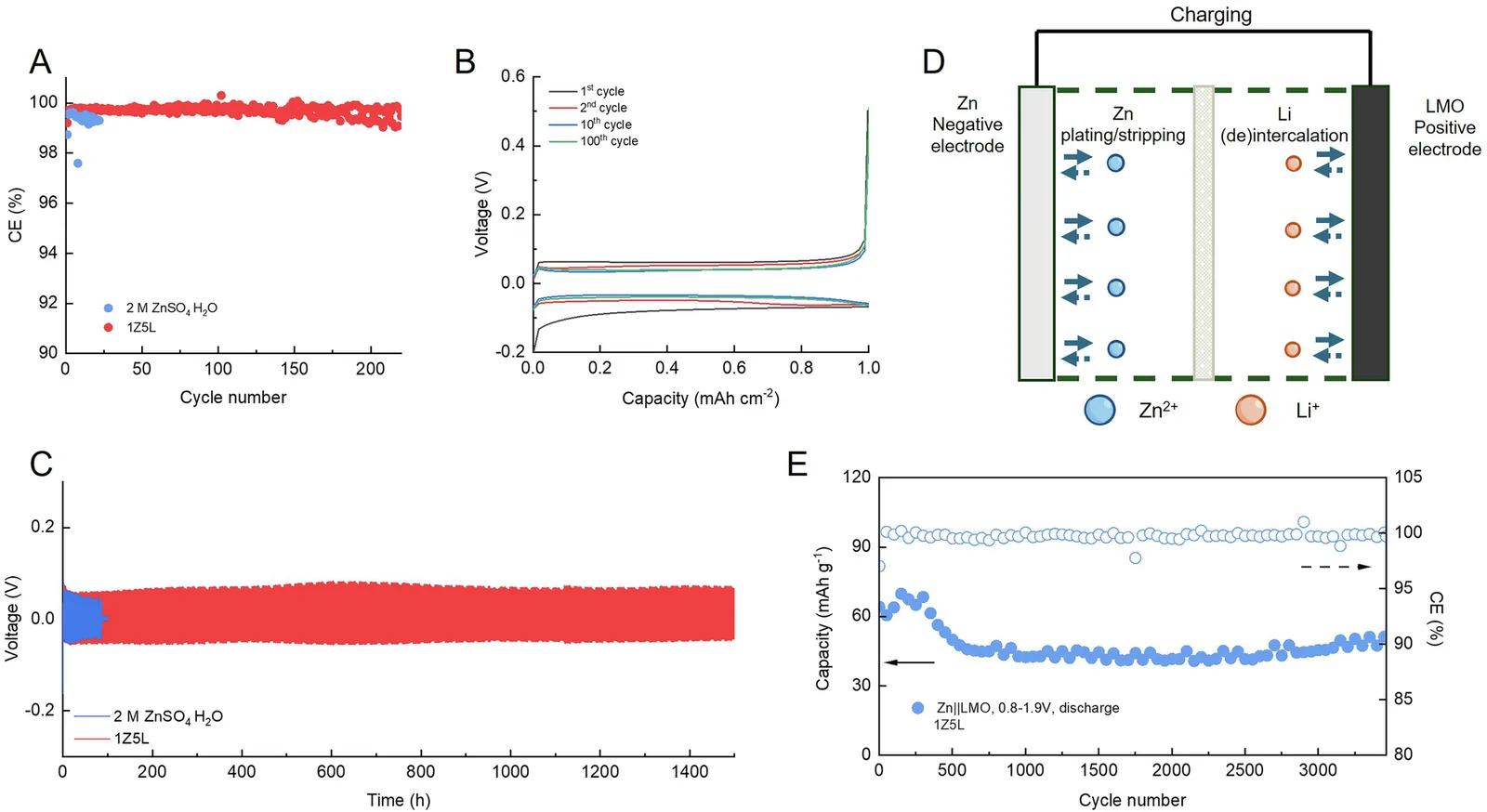
Comprehensive analysis of cycling performance. **A** CE performance at 1 mA cm^−2^ and 1 mAh cm^−2^ at 25 °C with a cutoff voltage of 0.5 V. **B** Voltage polarization profiles in different cycles observed in the half-cell. **C** Zn anode stability test at 1 mA cm^−2^ and 1 mAh cm^−2^. **D** Schematic of a Zn | |LMO full-cell configuration. **E** Cycling performance of the Zn | |LMO full-cell at a 4 C charge/discharge rate at 25 °C (theoretical capacity: 104 mAh g^−1^, 1 C = 104 mA g^−1^). Source data are provided as a Source Data file.

To demonstrate the long-term cycling performance of our designed electrolyte in various battery configurations, we first evaluate Zn plating and stripping in a Zn | |graphite paper half-cell (Supplementary Fig. 23). Typically, low Zn-ion concentrations will create a cation depletion region that promotes dendrite formation during Zn deposition^44^. However, adding Li_2_SO_4_ as a supporting salt improves ionic transport, mitigating short-circuit risks and enabling extended plating/stripping. Furthermore, DMF in the electrolyte favors SEI formation, providing an efficient ion pathway. Consequently, the Zn plating/stripping efficiency exceeds 99.0% in the first cycle at 1 mA cm^-2^ with a capacity of 1 mAh cm^-2^ and further increases to 99.7% over 220 cycles with stable voltage polarizations (Fig. 5A, B). In contrast, a 2 M ZnSO_4_-H_2_O electrolyte shows a steep decline in CE after only 23 cycles (Fig. 5A), underscoring the efficacy of our electrolyte design in achieving stable Zn plating/stripping over prolonged cycling. A Zn||Zn symmetric cell under galvanostatic conditions further illustrates the stability of Zn metal in the low-concentration electrolyte. While the 2 M ZnSO_4_-H_2_O electrolyte fails within 100 h at 1 mA cm^-2^ and 1 mAh cm^-2^, our low concentration electrolyte maintains stable voltage profiles beyond 1500 hours (Fig. 5C). We emphasize that despite the presence of free water molecules in the bulk electrolyte, consistent Zn plating/stripping performance is retained, suggesting that free water is less reactive than solvated water to Zn anode corrosion. To evaluate full-cell cycling performance, we then coupled Zn metal anodes with LiMn2O4 (LMO) cathodes (Fig. 5D), supplementing the low-concentration electrolyte with 0.05M MnSO4 to stabilise the LMO^4^. Under 4 C conditions, this cell achieves robust cycling stability, maintaining 80% of its capacity over 3300 cycles with an average CE of 99.8% (Fig. 5E and Supplementary Fig. 23), which is comparable with previous reports^2,14,45^. Guided by the above-mentioned electrolyte design principle, the Zn salt concentration can be modestly increased to 0.5 M, maintaining a similar low corrosion rate over an aging period of 24 hours (Supplementary Fig. 24). This electrolyte also demonstrates promising electrochemical performance in half-cells and symmetric-cells (Supplementary Fig. 25), as well as long-term cycling stability in full-cells with 78% capacity retention and a nearly 100% average Coulombic efficiency over 1000 cycles at 2 C (Supplementary Fig. 26A). Benefiting from the high reversibility on both the Zn anode (stripping/plating) and the LMO cathode (Li^+^ intercalation/deintercalation), the full-cell exhibits nearly 100% CE, highlighting the potential of this electrolyte design for practical zinc battery applications.

## Discussion

We demonstrate the effectiveness of a low-concentration regime (0.1 M ZnSO_4_) in mitigating corrosion driven by solvated water, thus highlighting an emerging design space that deserves increased attention. Compared to high-concentration or water-in-salt electrolytes^14^, this low-concentration electrolyte has a lower weight, thereby elevating the overall energy density. Low-concentration ZnSO_4_-based electrolyte that achieves both long-term cycling stability and markedly reduced capacity loss during calendar aging—by an order of magnitude compared to conventional systems. Detailed analyses of solvation structures reveal that corrosion is predominantly driven by solvated water molecules rather than free water, highlighting the importance of mitigating this reactive species to enhance long-term performance. Critically, our formulation balances ionic transport, SEI formation, and minimal corrosive reactivity—key elements for real-world implementation^46^. This strategy is not confined to Zn-based systems; aqueous Li-ion and Na-ion chemistries face similar hurdles, like narrow electrochemical stability windows^43^. Incorporating low salt concentrations, isotopic solvents, and carefully selected additives could enable broader application across multiple battery platforms. Ultimately, this electrolyte design highlights the necessity of integrating both aging mitigation and long-term cycling strategies early in the design process to produce long-lived aqueous batteries.

## Methods

### Material preparation

Preparation of electrolytes: All electrolytes were prepared by dissolving the salts into deionized (DI) water or deuterium oxide (D_2_O, Cambridge Isotope Laboratories, Inc., Deuterium oxide “100%” (D, 99.96%) low paramagnetic). Salts and organic additives include zinc sulfate (ZnSO_4_, Sigma Aldrich, 99%), lithium sulfate (Li_2_SO_4_, Sigma Aldrich, 99%), zinc trifluoromethanesulfonate (Zn(OTF)_2_, Fisher Scientific, ≥98%), and dimethylformamide (DMF, Sigma Aldrich, anhydrous, 99.8%).

Preparation of ZnMn_2_O_4_ (ZMO)/C: ZMO/C positive electrode (cathode) powder was synthesized by growing ZMO on carbon nanotubes^46^. 20 mL 0.2 M zinc nitrate (Zn(NO_3_)_2_, Fisher Scientific, ≥98%), 40 mL 0.2 M manganese nitrate (Mn(NO_3_)_2_, Sigma-Aldrich, ≥97.0%), and 640 mg carbon nanotubes (CNTs, US research Nanomaterials, 95%) were dissolved in a 200 mL beaker. 36 mL aqueous ammonia solution was added to the beaker and stirred for 1 h. Then, the mixture was evaporated in the oven at 180 °C for 3 h. The product precipitate was washed with DI water and ethanol 3 times, then centrifuged. The powder was vacuum-dried at 80 °C overnight in vacuum oven.

Preparation of ZMO/C positive electrode (cathode): The ZMO/C powder was combined with polyvinylidene fluoride (PVDF, Canrd Technology Co. Ltd, ≥99.5%) in a 6:4 mass ratio. The mixed powder was dispersed in N-methyl-2-pyrrolidone (NMP, Canrd Technology Co. Ltd, ≥99.9%) to make a slurry. Then, it was cast onto stainless steel foil (10 µm thick, 99.99%, Canrd Technology Co. Ltd.) using the doctor-blading method. Finally, the electrode was dried at 60 °C in a vacuum oven for 24 h.

Preparation of LiMn_2_O_4_ (LMO) positive electrode (cathode): The LMO powder (MTI Corporation), PVDF binder, and Super P (Canrd Technology Co. Ltd, 99%) with a mass ratio 7:2:1 was dispersed in NMP and cast onto stainless steel foil using the doctor blading method as above mentioned. The as-prepared slurry was dried overnight at 80 °C in a vacuum oven. The mass loading of ZMO and LMO is around 1-2 mg cm^-2^, and all positive electrodes (cathodes) were single-side coated.

### Electrochemical characterization

Zn foil (70 µm thickness, Leishent, 99%), graphite paper (70 µm thick, Digi-Key Electronics), Cu foil (10 µm thick, Canrd Technology Co. Ltd, 99%), and Ti foil (10 µm thick, Canrd Technology Co. Ltd, 99%) were punched into discs and rinsed with DI water and ethanol to remove surface contaminants before using as the current collectors. Glass fiber (260 µm thick, pore size 1.2 µm, Cytiva, Grade GF/C) was chosen as a separator, and one-piece separator was used in all half-cell, full-cell and symmetric-cell. For half-cells, Zn foil was used as the negative electrode (anode), with different substrates as the positive electrode (cathode). For graphite paper | |ZMO anode-free full-cells, graphite paper was used as a negative electrode (anode) substrate, and ZMO/C was used as a positive electrode (cathode). For Zn | |LMO hybrid full-cells, Zn metal and LMO were used as negative electrodes (anodes) and positive electrodes (cathodes), respectively. All aging experiments were conducted at 25 °C, and the electrolyte volume-to-electrode-area ratio was controlled to be 30 μL cm^-2^ for all half-cell calendar-aging tests, symmetric-cell tests, anode-free full-cell tests and full-cell cycling tests. This number falls within typical coin-cell electrolyte loadings reported in the literature and provides a practical balance between ensuring adequate wetting and avoiding unrealistically flooded conditions^8,47,48^.

Electrochemical data were collected on a LANDt battery tester at 25 °C using 2032-type coin-cells. The CE was measured using different substrates with a cut-off voltage of 0.5 V vs. Zn in half-cells without precycling procedure. Half-cells with aging were aged for 24 h at an open circuit after the plating step in the second cycle. For cycling performance, the graphite paper | |ZMO anode-free full-cell was cycling between 0.8-2.1 V at 0.5 mA cm^-2^ current density with 3 activation cycles with 0.05 mA cm^-2^ current density. The anode-free-cells with aging intervals were aged at 100% state of charge for 2 h every 5 cycles. The Zn | |LMO hybrid full-cell was cycling between 0.8-2.0 V/0.8-1.9 V with various current density after 2 activation cycles at 0.3 C using 1Z5L + 0.05 M MnSO_4_ otherwise specified (theoretical capacity: 104 mAh g^−1^)^45^. The formation cycle information is provided in Supplementary Fig. 27. The electrochemical performance values were calculated based on the discharge capacity. Cyclic voltammetry (CV) tests were conducted over a range of applied voltages scanning at 1 mV s^-1^ in Zn | |Zn symmetric cell. The apparent transference number (t^+^_app_) was tested in Zn | |Zn symmetric-cells using the protocol originally developed by Bruce and Vincent and widely used as a comparative transport metric. Briefly, cells were rested for 12 h, then polarized at ΔV = 10 mV for 1 h to reach steady state. Then, EIS was collected before/after polarization to obtain R_0_ and R_ss_, and t^+^_app_ was calculated following the Bruce-Vincent framework:

1 $${{{\rm{t}}}}_{+}=\frac{{{{\rm{I}}}}_{{{\rm{ss}}}}({\Delta {{\rm{V}}}-{{\rm{I}}}}_{0}{{{\rm{R}}}}_{0})}{{{{\rm{I}}}}_{0}({\Delta {{\rm{V}}}-{{\rm{I}}}}_{{{\rm{ss}}}}{{{\rm{R}}}}_{{{\rm{ss}}}})}$$

Ionic conductivity (σ) was measured using a symmetric stainless-steel blocking-electrode configuration (SS|electrolyte|SS) assembled in CR2032 coin-cells. A donut-shaped Celgard 2325 separator, saturated with electrolyte, was sandwiched between two stainless steel electrodes. Electrochemical impedance spectroscopy (EIS) measurements were performed in potentiostatic mode using a 10 mV sinusoidal voltage perturbation over a frequency range of 1 MHz to 0.1 Hz, with 10 points per decade. Unless otherwise stated, all EIS measurements were carried out at 25 ^o^C using the corresponding coin-cell configurations. The bulk ionic resistance (R_b_) was extracted from the high-frequency intercept of the Nyquist plot obtained via EIS. Ionic conductivity was calculated as

2 $${{\rm{\sigma }}}\,=\,\frac{{{\rm{L}}}}{{{{\rm{R}}}}_{{{\rm{b}}}}\times {{\rm{A}}}}$$

Where L is the compressed separator thickness and A is the cross-sectional area defined by the separator geometry. While the absolute conductivity values are subject to uncertainty arising from this geometric approximation, all measurements were performed under identical conditions, ensuring internally consistent comparisons across the electrolyte formulations.

### Material characterizations

Substrates were collected for characterization using scanning electron microscopy (SEM), transmission electron microscopy (TEM), and titration gas chromatography (TGC). All samples for material characterization were gently and thoroughly rinsed with DI water/D_2_O solvent to remove the salt residue. SEM images were taken with ZEISS Supra 40VP at an accelerating voltage of 10 kV. All TEM characterizations were performed using an FEI Titan 80-300 scanning transmission electron microscope (STEM) operated at 300 kV acceleration voltage. Thermogravimetry (TG) was performed on a Perkin Elmer Diamond Thermogravimetric analyzer from ambient temperature to 600 °C at a linear heating rate of 5 °C min^-1^ under an air atmosphere. The chemical bond signals of electrolytes were identified with the Fourier-transform infrared spectroscopy (FTIR) technique using Agilent Cary 670 spectrometers from 4000 to 700 cm^-1^ with 500 scans and 4 cm^-1^ wavenumber resolution. Focused-ion-beam scanning electron microscopy (FIB-SEM, Nova 600) was performed using a Ga^+^ source. Electron imaging was carried out at 10 kV and 0.54 nA, while ion milling was conducted at 30 kV, using 5 nA for trench cutting and 0.5 nA for final surface cleaning. The XPS spectra were acquired using a Kratos Axis Ultra DLD spectrometer equipped with a monochromatic Al Kα X-ray source. Fittings of the XPS spectra were performed with CasaXPS software (Casa Software Ltd., version 2.3.15) to determine the chemical species. High-resolution spectra were calibrated using the C 1 s peak at 284.8 eV and fit using a Shirley-type function background. The peak positions and areas were optimized by a Gaussian/Lorentzian product peak shape function, following the literature.

Titration gas chromatography (TGC) method: TGC measurements were carried out using an Agilent 6890 N GC attached to a 5975 MSD (Agilent Technologies) operated in the positive electron ionization mode. The instrument was controlled by Enhanced Chemstation software version E.01. Gas samples were introduced manually via an all-polypropylene syringe fitted with a 27-gauge non-coring needle and injected into a 200 °C inlet set to splitless mode. A J&W 30 m × 320 μm × 25 μm HP-PLOT Molesieve 5 A column (Agilent Technologies) was used for separation. UHP He (Airgas West) was used as the carrier gas, with the flow rate set to 1.0 mL min^-1^. The GC oven was held at 40 °C for the duration of the measurement. Retention times for the HeH species were identified by injecting pure H_2_ gas (Airgas West, ultra-high purity). The MSD was set to selected ion monitoring (SIM) mode, targeting the m/z 5 ion within the retention time window expected for HeH. Signal intensities for HeH were quantified by integrating the area under the extracted ion chromatogram for m/z 5. Hydrogen abundance in unknown samples was determined by comparing their responses to calibration injections of known H₂ volumes.

To assign the measured signal to Zn^0^ and Zn^2+^ proportions, we have the following key assumptions and formulas:

1. All metallic Zn^0^ residues react completely with injected HCl in the sealed vial as follows:
  $${{\rm{Zn}}}\,+\,{{\rm{2HCl}}}\to {{{\rm{ZnCl}}}}_{2}{+{{\rm{H}}}}_{2}$$
2. The generated H_2_ is fully retained and well-mixed before sampling (we shake the vial to avoid gas stratification).
3. Electrochemical capacity is converted to Zn mass using Zn’s theoretical specific capacity of 819 mAh g^-1^, or equivalently, 1 mAh Zn means 1.221 mg Zn.
4. The number of Zn metal mass within the container (m_Zn_) versus H_2_ peak area is based on the relationship 1 p.p.m. = 4.08 × 10^-8 ^mmol mL^-1^ at 1 atm and 298 K.

### Theoretical calculations

Density functional theory calculations were performed using the plane-wave-based Vienna Ab Initio Simulation package (VASP)^49^. The electronic exchange and correlation effects were described by the generalized gradient approximation (GGA) in the form of the Perdew-Burke-Ernzerhof (PBE) functional^50^. The D3 correction method was employed to illustrate the long-range dispersion interactions between the water and surface^51^. The interaction between atomic cores and electrons was described by the projector augmented wave (PAW) method^50^. A cutoff energy of 400 eV for the plane-wave basis set and an atomic force convergence of 0.02 eV/Å were employed. The transition state search was conducted with the climbing image nudged elastic band (CI-NEB) method, followed by the dimer method to converge the saddle point within 0.05 eV/Å. The Zn (002) surface was modeled by a 3-layer (4 × 4) supercell^52^. The bottom layer of the slab was constrained as the bulk region, and the top two layers and adsorbates were allowed to relax as the interface region. The Brillouin zone was sampled using the 4 × 4 × 1 Gamma-centered k-point grids. Two layers of water with and without a Zn ion represent the explicit solvent region. The linearized Poisson-Boltzmann implicit solvation model implemented in VASPsol is used to represent the implicit electrolyte region. The dielectric constant of water, 78.4, and the Debye screening length corresponding to 1 M concentration of electrolytes, 3.0 Å, were used. We used the EChO Python package to achieve the constant-potential modeling, including structural optimization and transition state searching, which is rooted in the surface charging method. The original data are provided in Supplementary Data 1.

## Supplementary information

Supplementary Information

Description of Additional Supplementary Files

Supplementary Data 1

Transparent Peer Review file

## Source data

Source Data

## Data Availability

The source data generated in this study are provided in the Source Data file. Source data are provided with this paper.
