# Supplementary material for: Dilute electrolytes for suppressing metal anode corrosion during calendar aging and cycling in aqueous zinc batteries
Source: Nat Commun. 2026 Aug 6;17:9463. doi: 10.1038/s41467-026-75100-x (PMC13542140; doi:10.1038/s41467-026-75100-x)
Supplement: Supplementary file 1 [file 41467_2026_75100_MOESM1_ESM.pdf]

Supplementary Information

Dilute electrolytes for suppressing metal anode corrosion during calendar aging and cycling in aqueous zinc batteries

Haoyang Wu,[#1] Bo Liu,[#1] Dingyi Zhao,[#1] Dongfang Cheng,[1] Keyue Liang,[1] Xintong Yuan,[1] Kaixi Chen,[1] Min-Ho Kim,[1] Kaiyan Liang,[1] Jung Tae Kim,[1] Jiayi Yu,[1] Tian-Yu Wang,[1] Philippe Sautet,[1,2,3] and Yuzhang Li[*1, 2]

[#]Contributed equally

[1]Department of Chemical and Biomolecular Engineering, University of California, Los Angeles, CA 90095, United States

[2]California NanoSystems Institute (CNSI), University of California, Los Angeles, CA 90095, United States

*Corresponding author: yuzhangli@ucla.edu

Supplementary Notes

Supplementary Note 1: Crystal Orbital Hamilton Population (COHP) Analysis of O–H Bonding Free and Solvated Water

To better understand the activity of proton donors in free water and solvated water, and their role in the hydrogen evolution reaction (HER), we performed density functional theory (DFT) calculations. The Zn(002) surface was used as a model, as confirmed by experimental TEM results. Two layers of water were incorporated to represent the local solvent environment. To investigate the effect of Zn ions on proton donor activity, a Zn ion was introduced into the solvent layer. Constant-potential DFT structural optimizations were then performed at –0.8 V vs. standard hydrogen electrode (SHE) to simulate the potential at which HER typically occurs. The calculations show that the Zn ion becomes solvated by four surrounding water molecules, forming Zn–O bonds.

The ability of water molecules to donate protons is directly linked to the strength of their O–H bonds where weaker bonds facilitate bond cleavage and proton transfer. To quantitatively analyze differences in O–H bond strength, we performed COHP analysis (Supplementary Fig. 1A-B), a method that decomposes the electronic structure into bonding, non-bonding, and anti-bonding contributions, on the O–H bonds in both free and solvated water. The integrated COHP (–ICOHP) values provide a quantitative measure of bond strength, with more negative –ICOHP values corresponding to stronger bonds. Our results indicate that the O–H bond in free water (denoted as O–H@H_2O) exhibits substantial bonding character (Supplementary Fig. 1C). In contrast, the O–H bond in solvated water (O–H@Zn(H_2O)) shows reduced bonding contributions and a pronounced anti-bonding feature near the Fermi level (Supplementary Fig. 1D). This anti-bonding character decreases the –ICOHP value, indicating a much weaker bond in solvated water compared to free

water. As a result, the O–H bond in solvated water is more prone to cleavage, enhancing its proton-donating ability. These electronic structure results demonstrate that Zn ions modulate O–H bond strength via anti-bonding interactions, thereby facilitating proton transfer in HER.

Supplementary Note 2: Energy Barrier Calculations for Proton Donation

To quantify the proton-donating abilities of free and solvated water, we calculated the minimum-energy reaction pathway for the Volmer step, which is widely recognized as the rate-determining step in HER. In the case of free water, proton transfer typically involves migration between adjacent water molecules, ultimately forming a hydroxide ion. Although this OH^- is partially stabilized by hydrogen bonding, the overall process requires substantial O–H bond cleavage and proton migration. Consequently, the reaction barrier is relatively high (~1.2 eV), and the overall reaction energy is about 0.6 eV (Figure 2A),[1] primarily due to the limited stabilization of the OH^- ion in solution.

In contrast, when Zn ions are present, we examined two scenarios involving proton donation from solvated water: (1) direct proton donation from a 1st shell water molecule, and (2) donation from a 2nd shell water molecule, assisted by a Zn-coordinated water molecule. In the first scenario, the resulting OH^- binds strongly to the Zn ion, forming a $Zn^{2+}(H_2O)_3OH^-$ complex (Figure 2B). This stabilization of both the transition and final states lowers the reaction barrier and energy by ~0.6 eV and ~0.4 eV, respectively, compared to the free water case (Figure 2A). In the second scenario, proton donation occurs from a 2nd shell solvated water via a Grotthuss-like mechanism, in which the proton transfer is mediated by a Zn-coordinated water molecule . This forms a transition state represented as Zn^{2+}–OH–H–OH, further stabilized by the Zn ion (Figure 2B). The final state in both scenarios involves the OH^- directly coordinated to Zn^{2+} (Figure 2B). Overall, our explicit reaction path modeling demonstrates that Zn ions substantially facilitate proton donation by stabilizing both the transition and final states through strong Zn–OH interactions. This stabilization lowers the energy required for O–H bond cleavage in solvated water, making the proton transfer

process more favorable and enhancing the HER kinetics.

Supplementary Figures

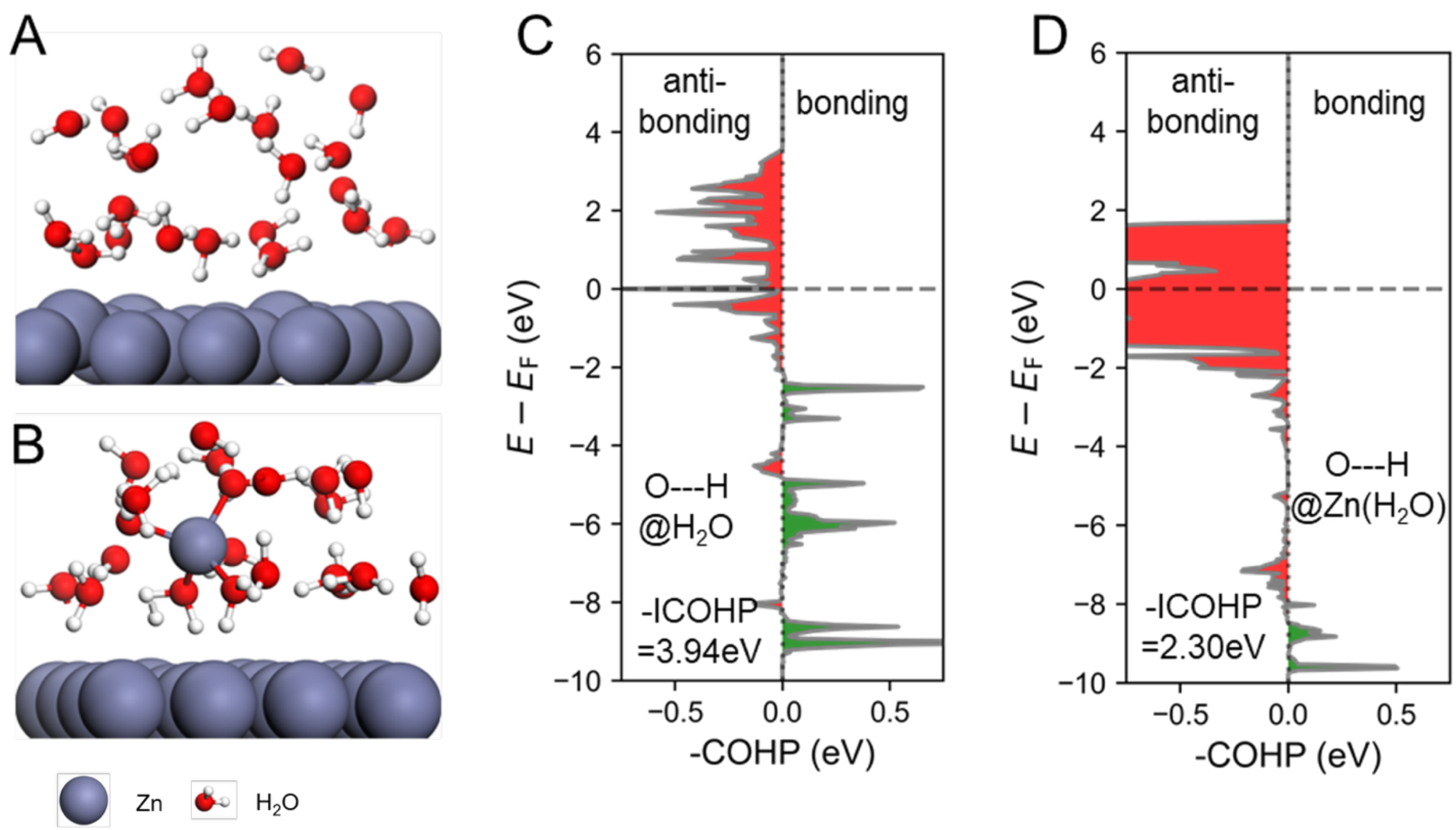

Supplementary Fig. 1. Simulation of bonding energy difference between free water molecules and solvated water molecules. The atomic structure of (A) Zn (002)-water interface and (B) Zn (002)-water-Zn^{2+} interface. The Crystal Orbital Hamilton Population (COHP) analysis of O-H bond in (C) pure water molecule and (D) water molecule which binds to Zn^{2+}.

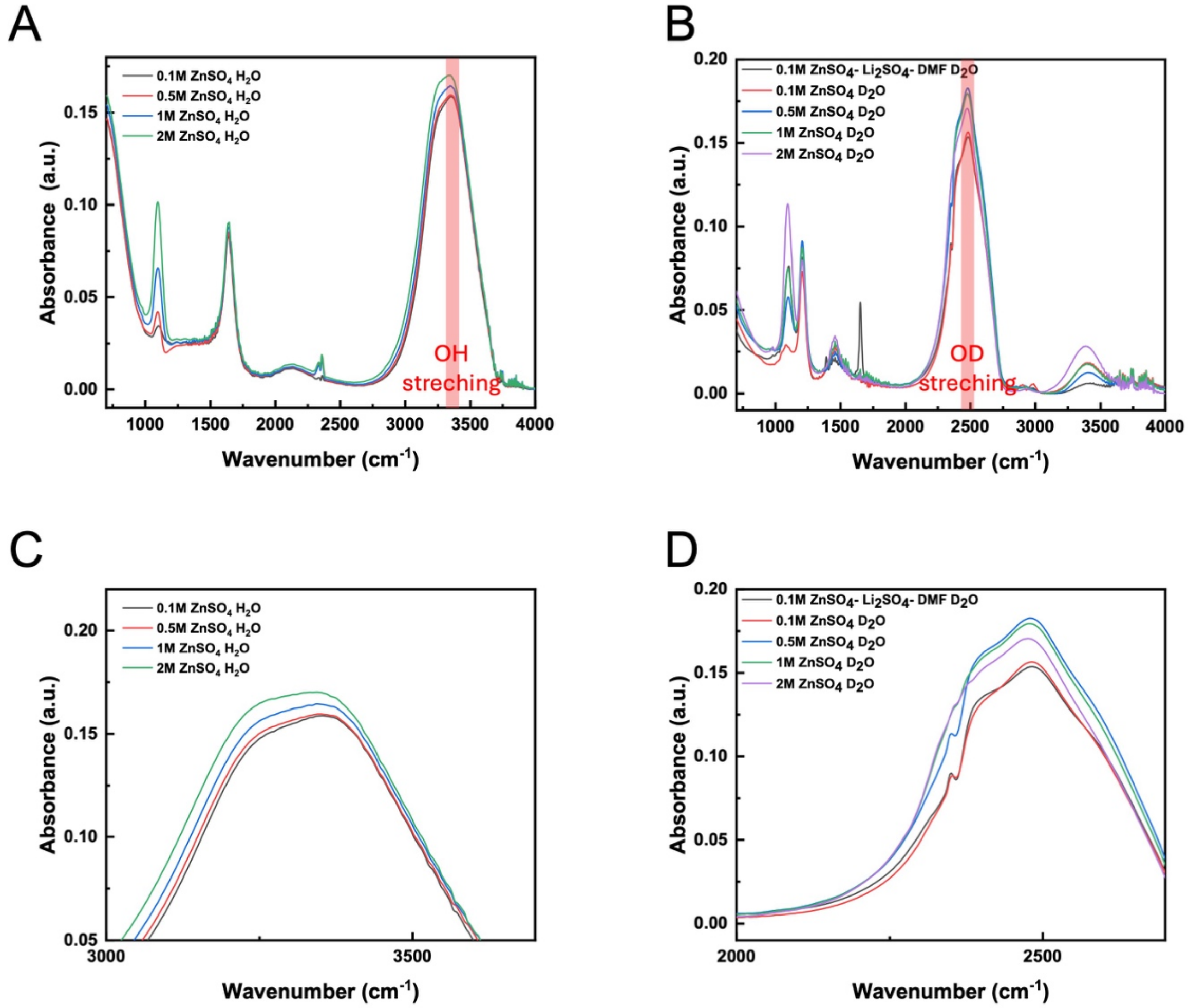

Supplementary Fig. 2. The effect of Zn^{2+} concentration on the water reactivity. Fourier Transform Infrared Spectroscopy (FTIR) spectra between 700 and 4,000 cm^{-1} with different salt concentrations in (A, C) H_2O-based electrolyte and (B, D) D_2O-based electrolyte.[2] Source data are provided as a Source Data file.

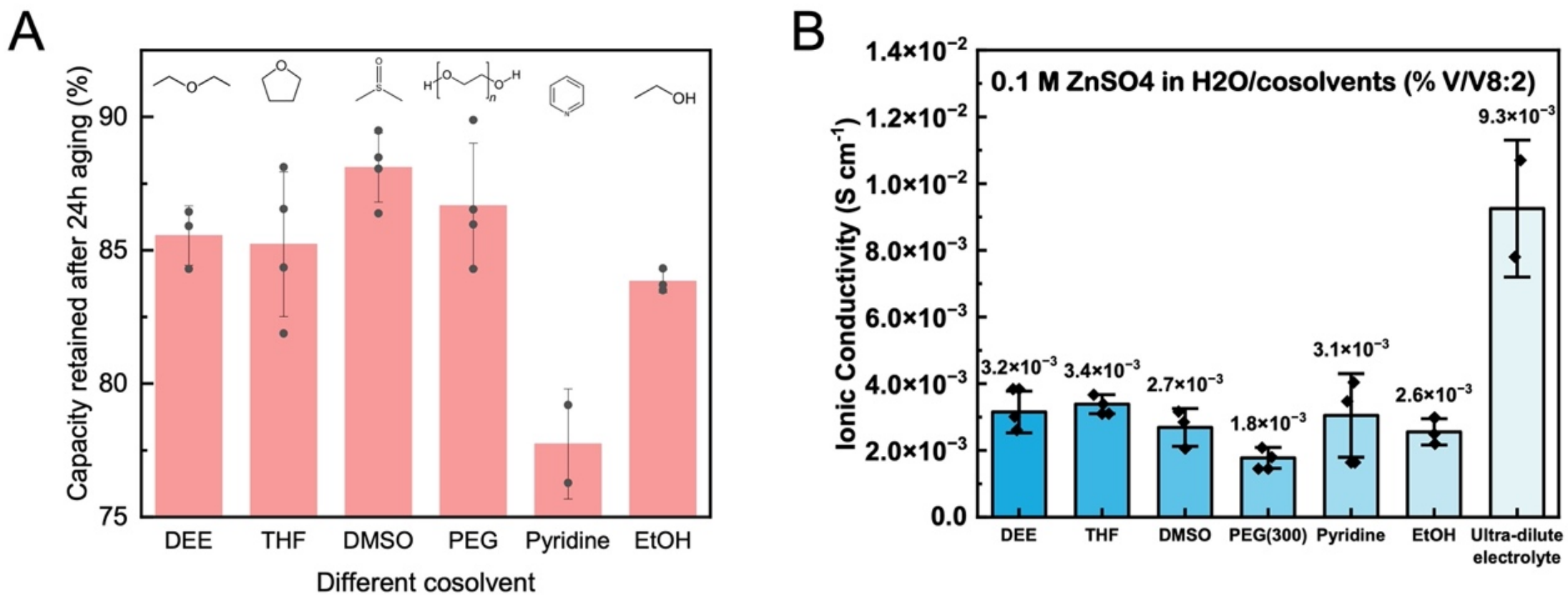

Supplementary Fig. 3. (A) Calendar aging performance and (B) ionic conductivity of different cosolvent electrolytes: 0.1 M $ZnSO_4$ in H_2O/cosolvent mixture (with volume ratio 8:2). Various organice cosolvents include Diethyle ether (DEE), Tetrahydrofuran (THF), Dimethyl sulfoxide (DMSO), Polyethylene glycol (PEG 400), Pyridine, Ethanol (EtOH) (with a Zn deposition capacity of 1 mAh cm^{-2} at 25 °C under 1mA cm^{-2} current density, using graphite paper as substrate). Error bars on plots show the standard deviation of 3 to 4 independent samples. To quantitatively contextualize feasibility and clarify the transport trade-off in the low-concentration regime, we measured the ionic conductivity of each 0.1 M $ZnSO_4$ electrolyte used in the co-solvent screening (H_2O/co-solvent = 8:2, v/v) and summarized the results in Supplementary Fig. 3B. Ionic conductivities show that co-solvents decrease the ionic conductivities, indicating a shift toward transport-limited behavior. Source data are provided as a Source Data file.

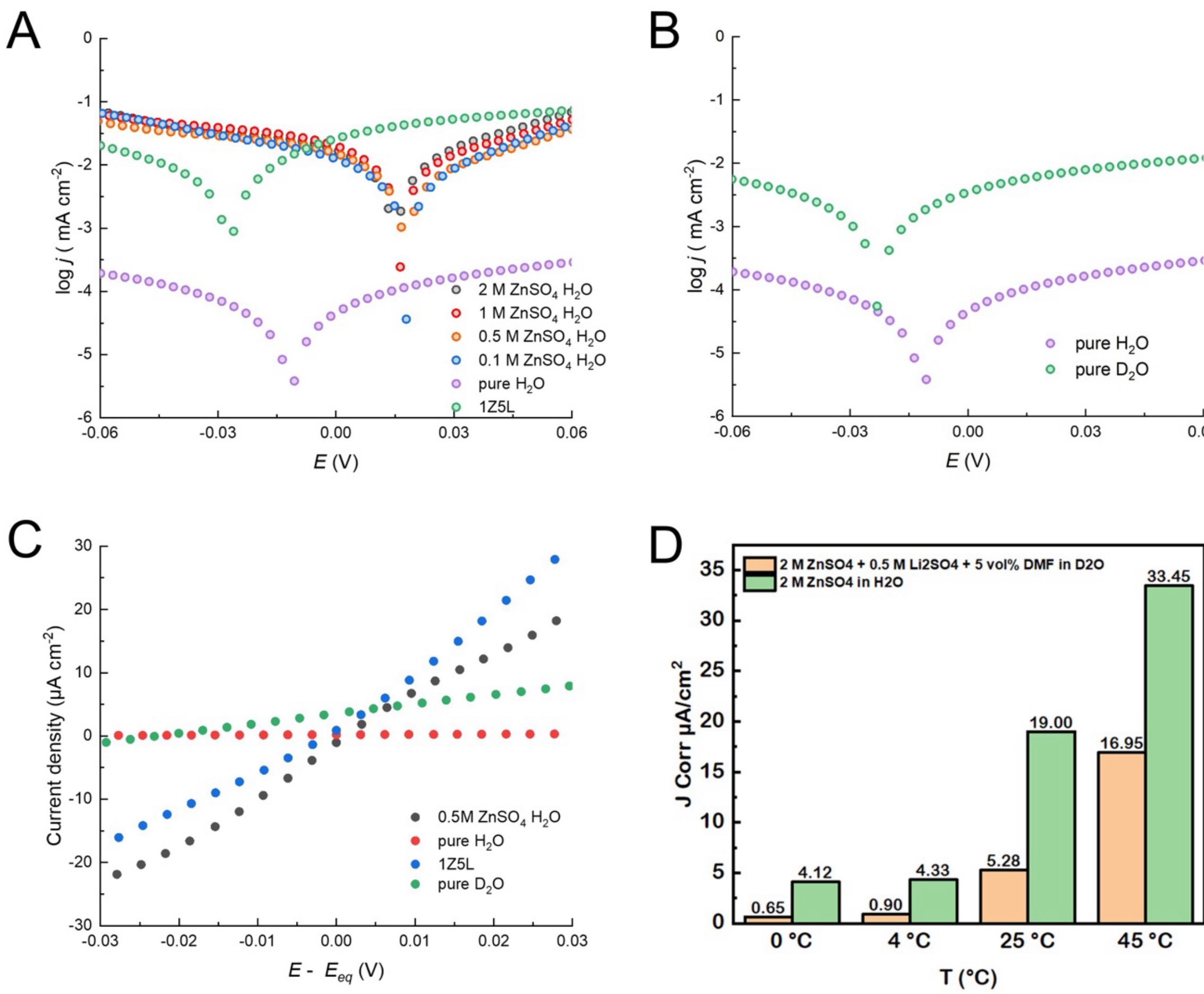

Supplementary Fig. 4. Tafel analysis. (A-B) Tafel plot of different electrolytes derived by LSV measurements. (C) Representative LSV data. (D) Corrosion current density from Tafel analysis at different temperatures. Source data are provided as a Source Data file.

To further confirm the solvation structure regulation and the hydrogen bonding behavior at different temperatures, we conducted Tafel analyses at 0, 4, 25, and 45 °C to compare 2 M $ZnSO_4$ in H_2O and 2 M $ZnSO_4$ + 0.5 M Li_2SO_4 + 5 vol% DMF in D_2O (Supplementary Fig. 4D). The electrolyte with additive and solvent modifications exhibits a lower corrosion current density across different temperatures, highlighting DMF-assisted SEI formation with solvation modulation and reduced HER kinetics in D_2O (kinetic isotope effect) that protects Zn during calendar aging.

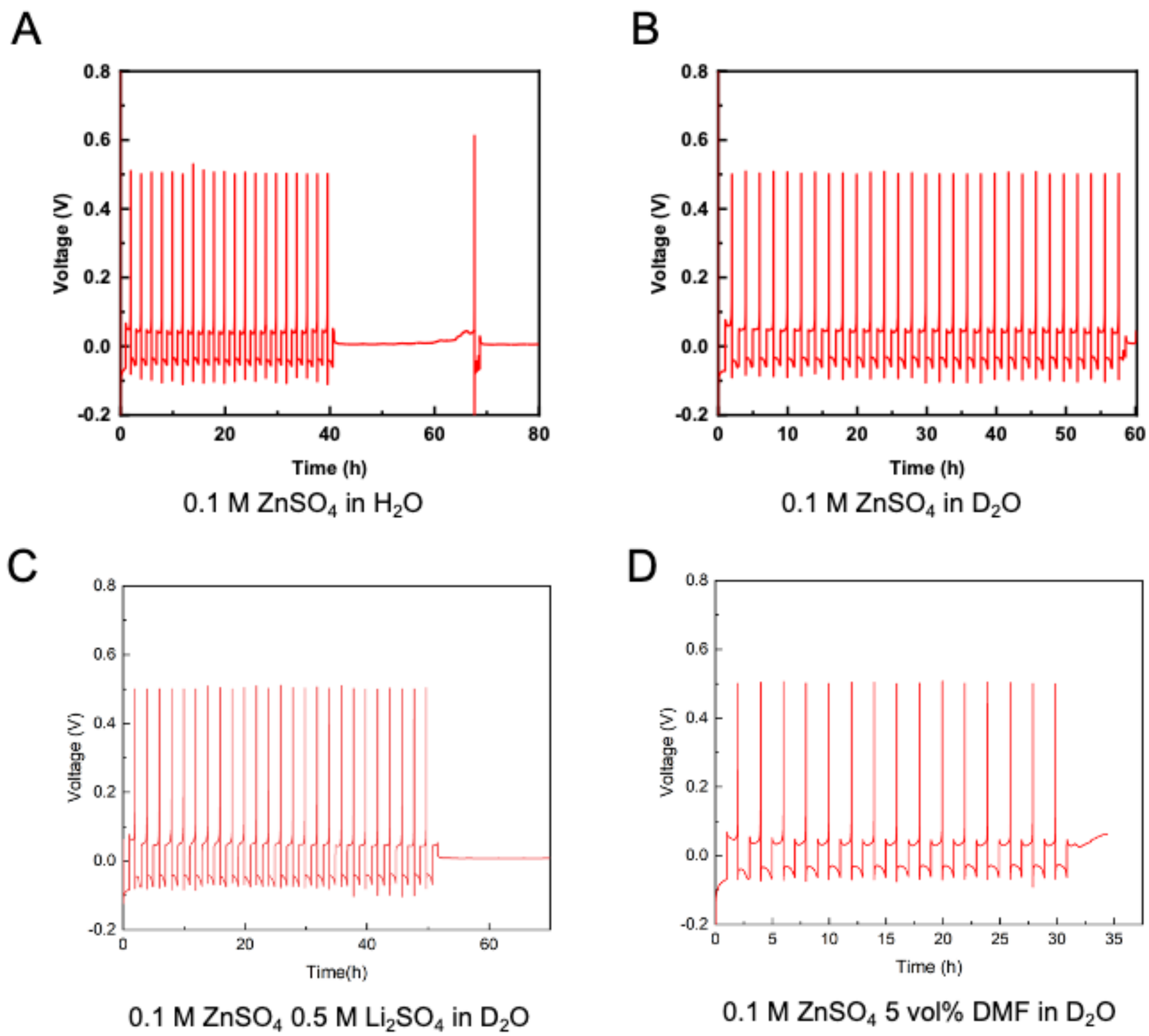

Supplementary Fig. 5. Long-term stability in electrolytes with low Zn^{2+} concentration. Voltage profiles of Zn stripping and plating in (A) 0.1 M $ZnSO_4$ in H_2O, (B) 0.1 M $ZnSO_4$ in D_2O, (C) 0.1 M $ZnSO_4$ + 0.5 M Li_2SO_4 in D_2O, and (D) 0.1 M $ZnSO_4$ + 5 vol% DMF in D_2O at 1 mA cm^{-2} and 1 mAh cm^{-2}. Source data are provided as a Source Data file.

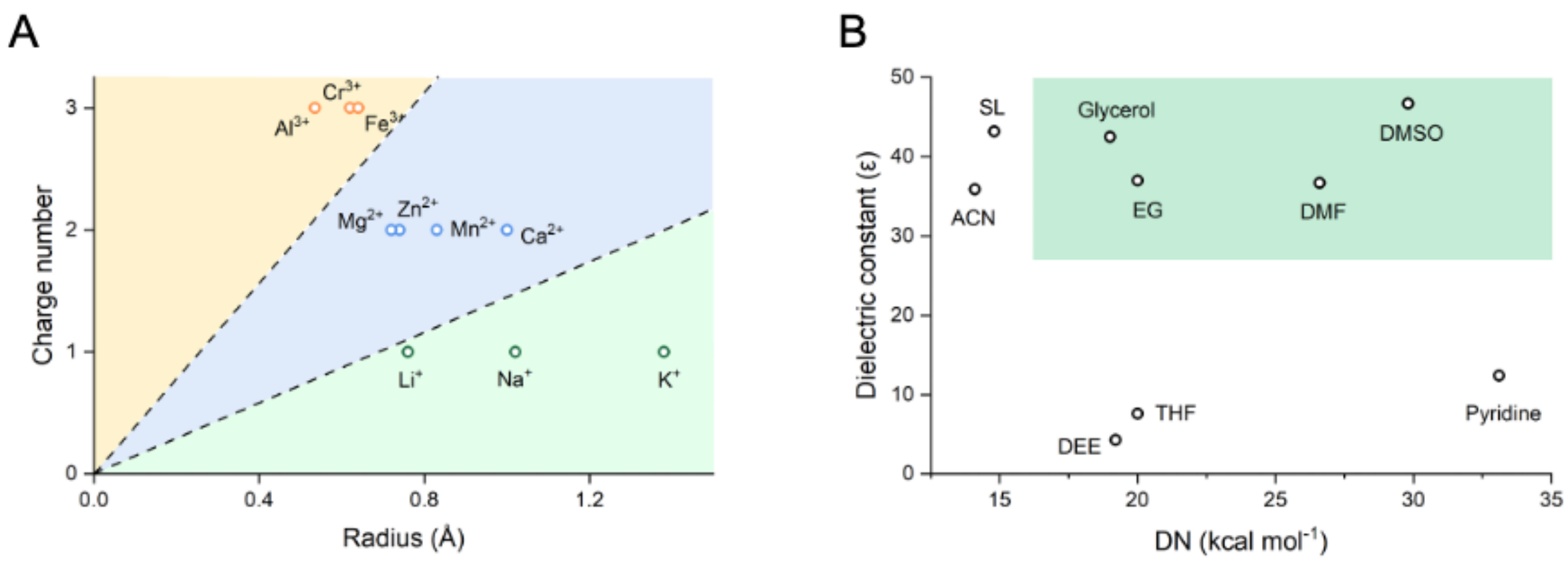

Supplementary Fig. 6. Selection of cations and organic additives. (A) Different cations with their ionic potentials. (B) Organic molecules with different dielectric constant and DN.[3] Source data are provided as a Source Data file.

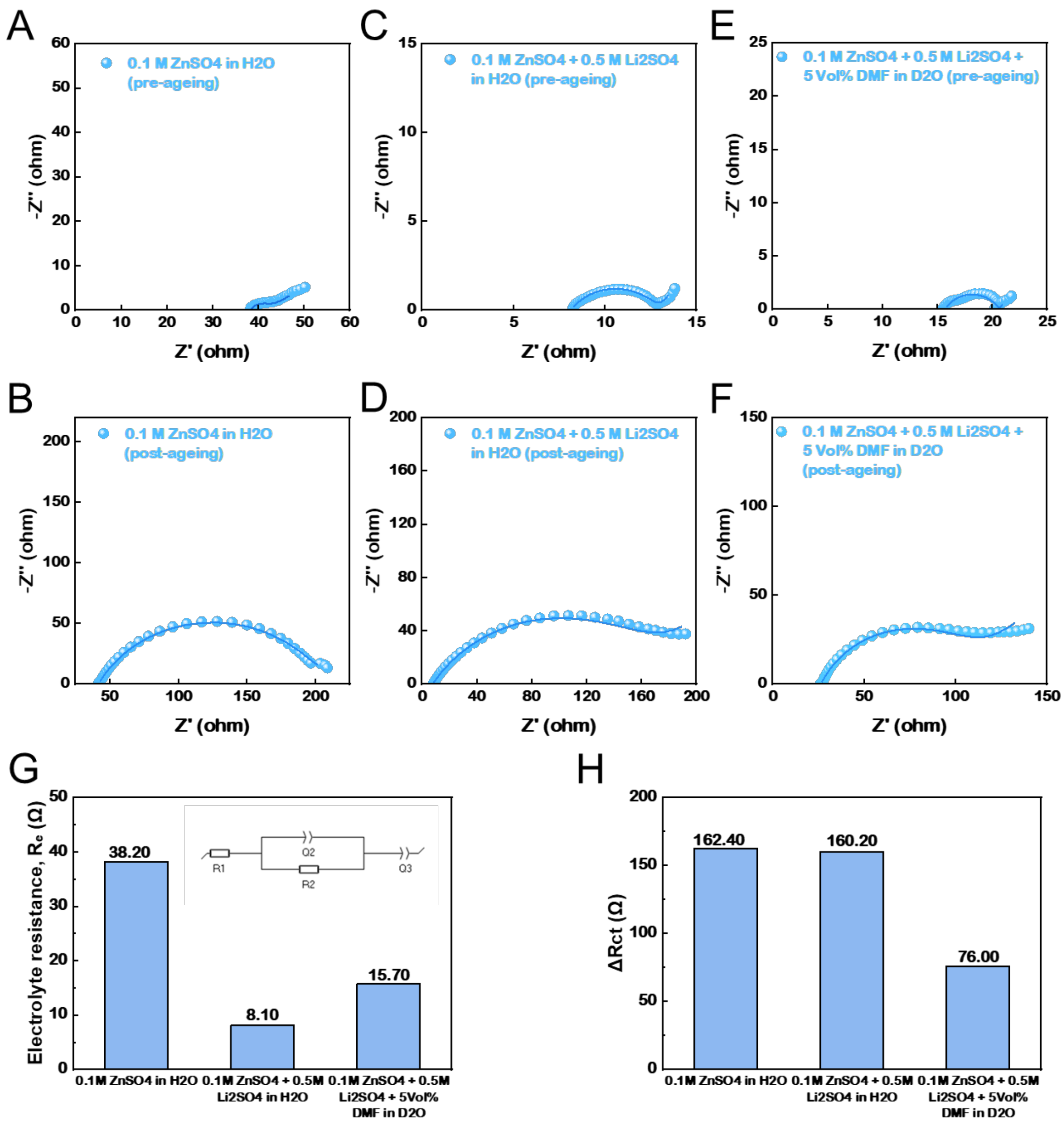

Supplementary Fig. 7. Cation concentration effects on electrolyte ionic conductivity. EIS profile before and after aging in (A-B) 0.1 M $ZnSO_4$ in H_2O, (C-D) 0.1 M $ZnSO_4$ + 0.5 M Li_2SO_4 in H_2O, and (E-F) 0.1 M $ZnSO_4$ + 0.5 M Li_2SO_4 + 5 vol.% DMF in D_2O with equivalent circuit for fitting inset in (G). Summarized (G) electrolyte resistance and (H) aging induced charge transfer resistance growth. Source data are provided as a Source Data file.

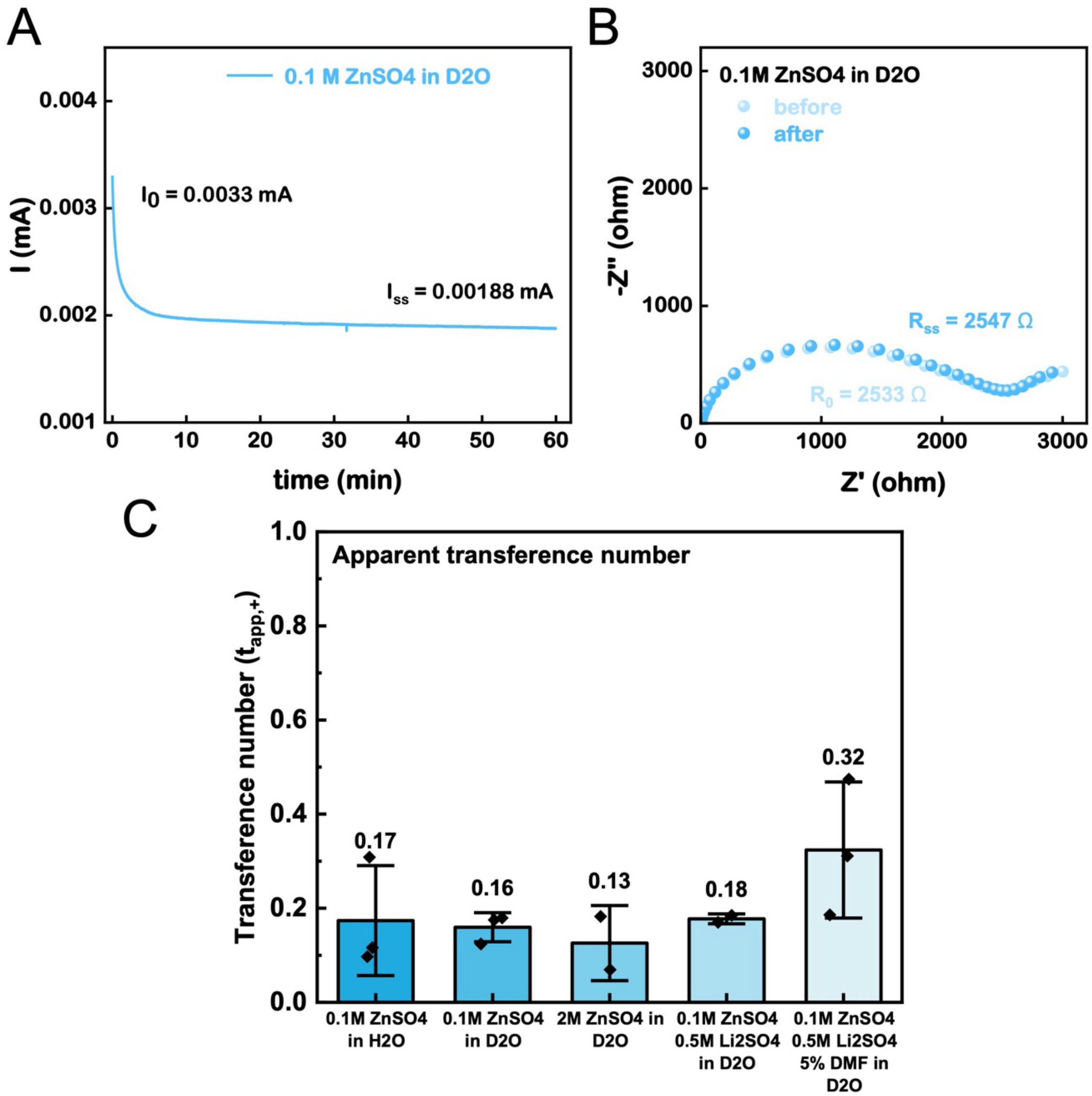

Supplementary Fig. 8. (A-B) Bruce Vincent method and (C) apparent transference number. Error bars on plots show the standard deviation of 3 independent samples. Source data are provided as a Source Data file.

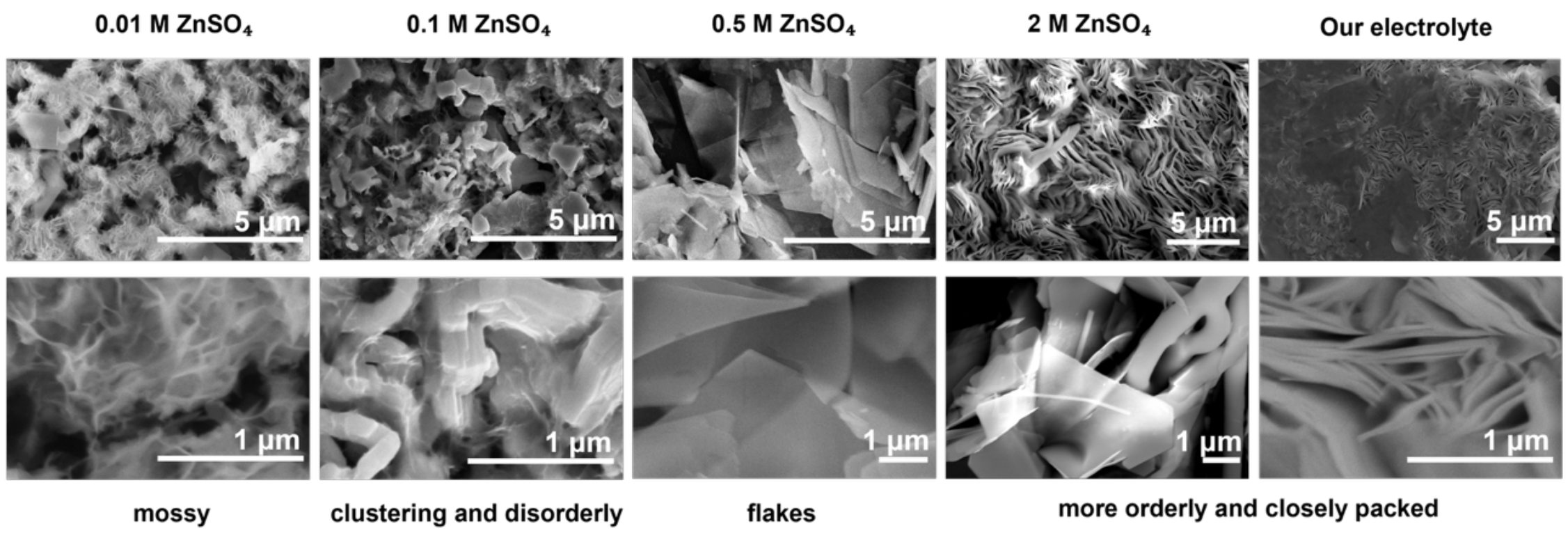

Supplementary Fig. 9. Zn deposition morphology at 1 mA cm^{-2} and 1 mAh cm^{-2} across different electrolytes.

In classical electrodeposition theory, lowering the salt concentration shortens the transition time to interfacial ion depletion, often discussed in terms of Sand's time, thereby increasing the propensity for dendritic growth.[4, 5] Consistent with classic analyses and prior Zn electrodeposition studies,[4, 6] we also observe a clear concentration-dependent morphological evolution, where higher concentrations yield orderly and closely packed deposits, whereas lower concentrations lead to increasingly clustered, and ultimately mossy and dendritic features, indicative of stronger mass-transport control and interfacial depletion (Supplementary Fig. 9). We clarify that low $ZnSO_4$ concentration alone does not guarantee dendrite-free Zn deposition. Instead, our central design principle is to reduce Zn^{2+} concentration, thereby minimizing corrosive solvated water (which drives calendar-aging stability), while dendrite suppression during cycling requires a complementary interfacial/morphological-regulation strategy. Samples for TEM were prepared by drop-casting after the half-cell had been cycled for 3 cycles at an areal capacity of 1 mAh cm^{-2} and a current density 1 mA cm^{-2}.

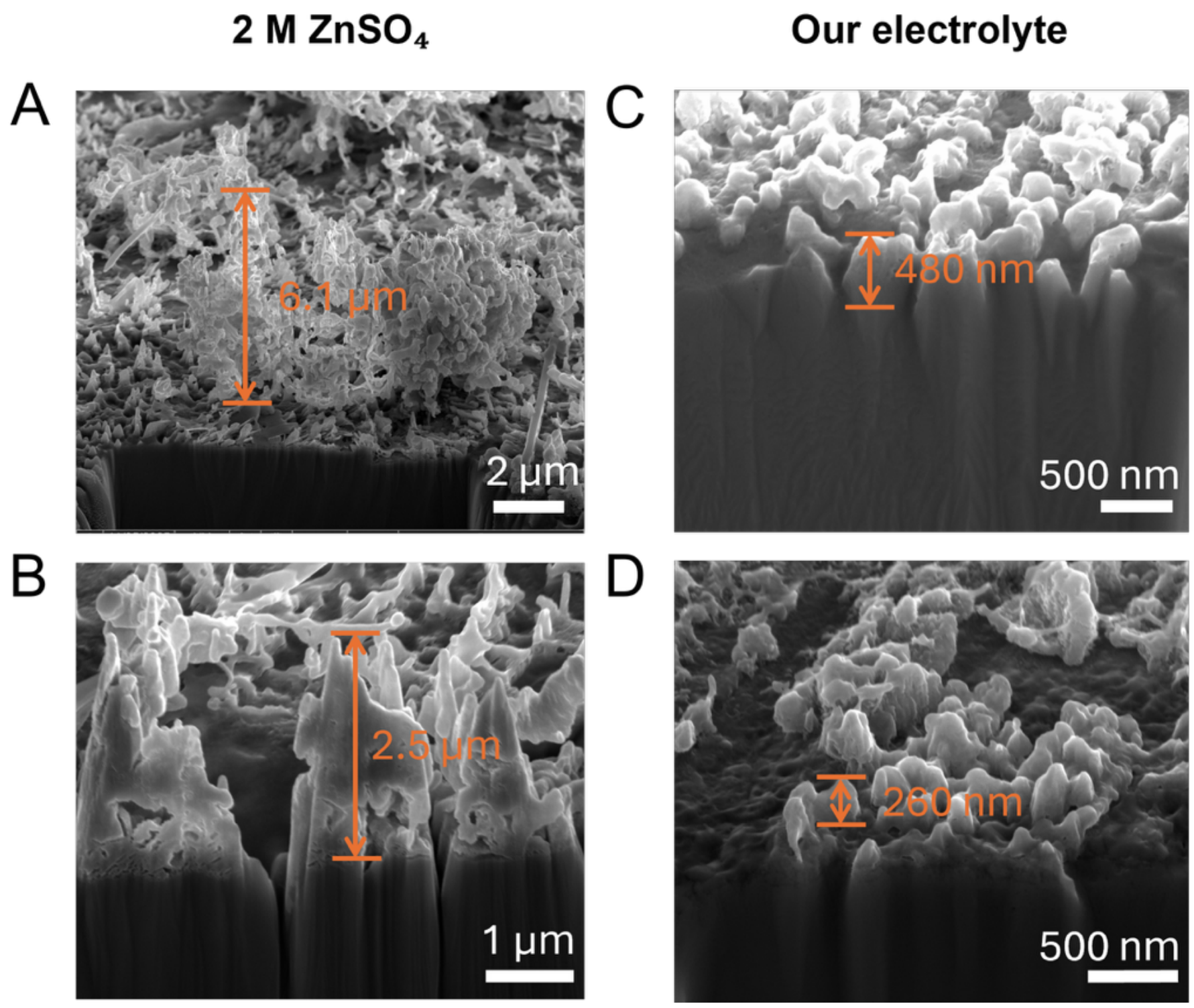

Supplementary Fig. 10. FIB-SEM cross-sectional images. (A-B) 2 M $ZnSO_4$ and (C-D) our optimized electrolyte. All samples were prepared by depositing 3 mAh cm^{-2} Zn metal on graphite paper substrate at 20 mA cm^{-2}.

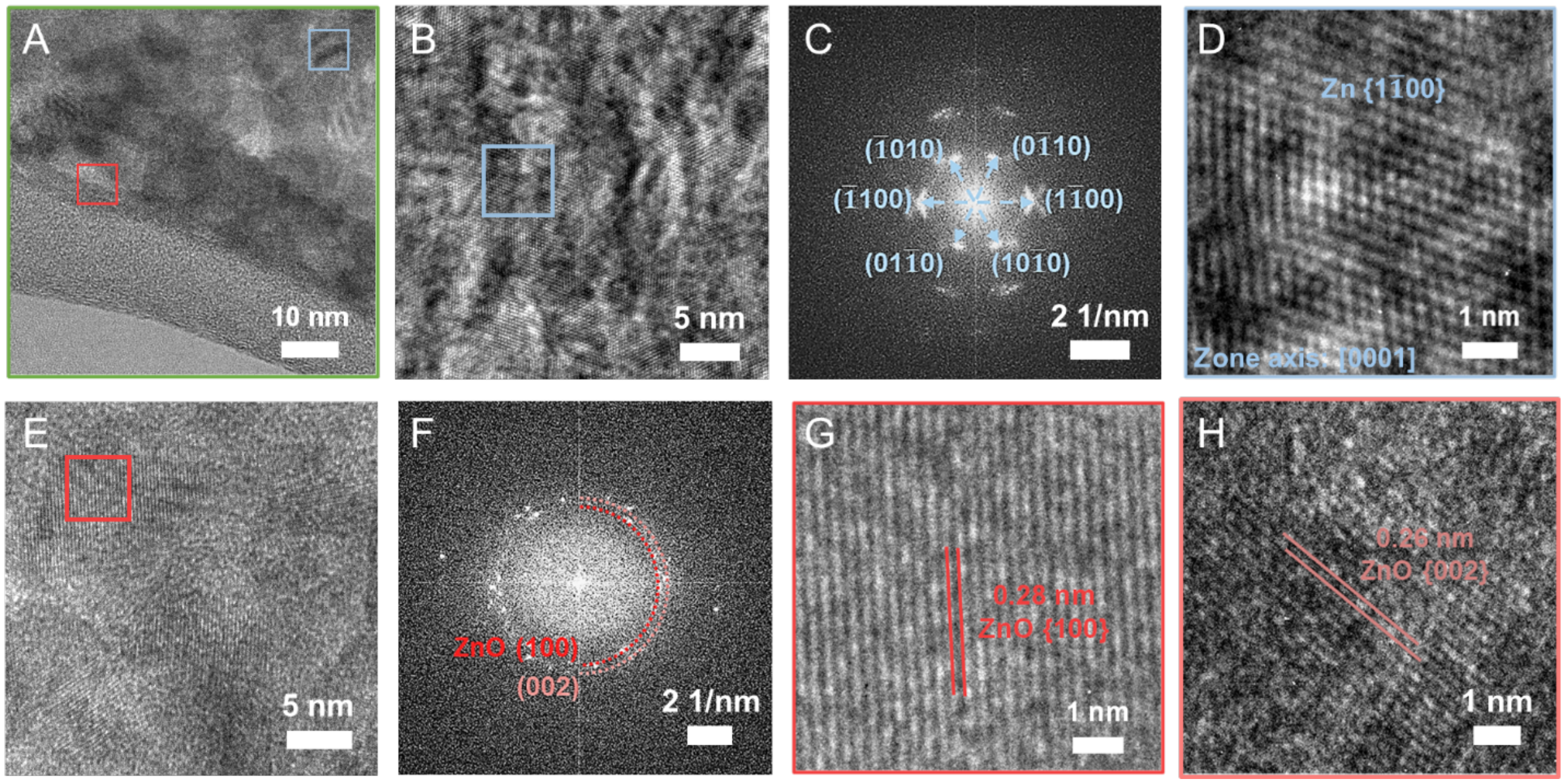

Supplementary Fig. 11. FFTs and zone-axis identification. FFTs and HETEM images from the representative (A) HRTEM ROIs (Fig. 3B). The indexed reflections in (B-C) and (E-H) are consistent with wurtzite ZnO ($P6_3mc$) and hcp Zn ($P6_3/mmc$), respectively, with zone-axis identification added in (D). Electrodes for TEM analysis were collected from half-cells after three cycles at 1 mAh cm^{-2} and 1 mA cm^{-2}.

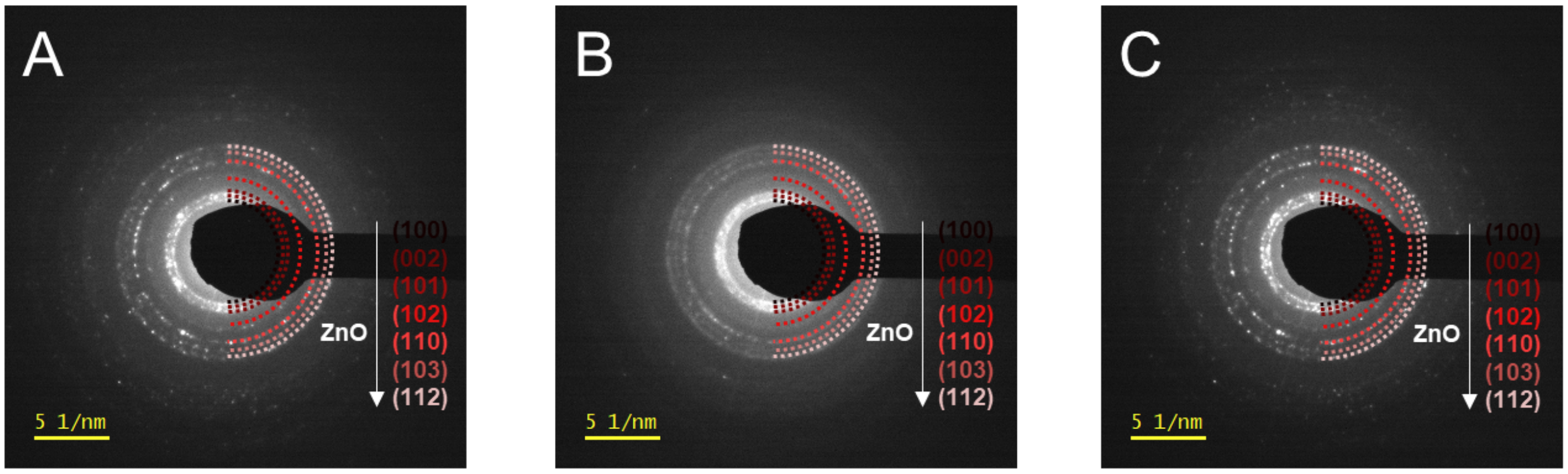

Supplementary Fig. 12. (A-C) Three SAED patterns collected from different regions and overlaid the expected ring positions for ZnO with lattice planes labeled in the overlays. Sample preparation method is same as Supplementary Fig. 11.

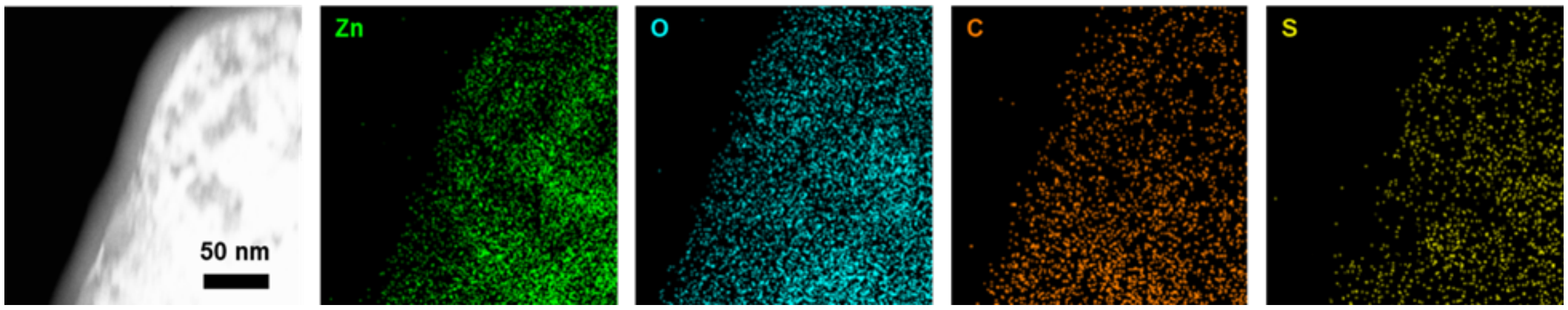

Supplementary Fig. 13. Incorporating DMF to facilitate stable SEI formation. TEM-EDS images displaying Zn, O, C, and S distributions. Sample preparation method is same as Supplementary Fig. 11.

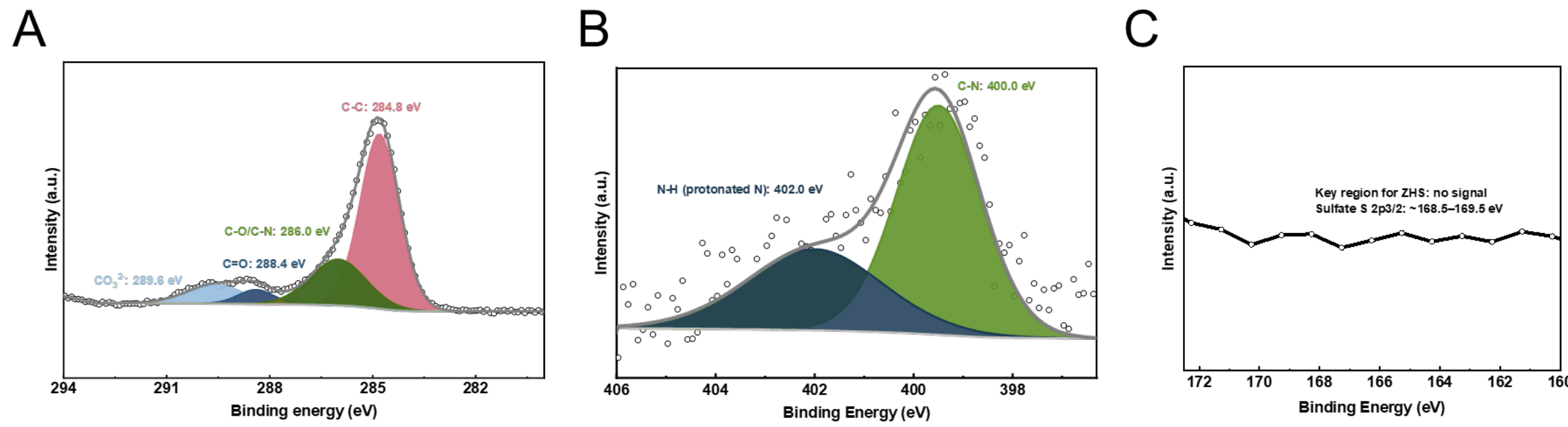

Supplementary Fig. 14. Post-cycling XPS analysis on the surface. (A) C 1s, (B) N 1s, and (C) S 2p. Samples were obtained from electrodes after 3 cycles at 1 mAh cm^{-2} and 1 mA cm^{-2}. Source data are provided as a Source Data file.

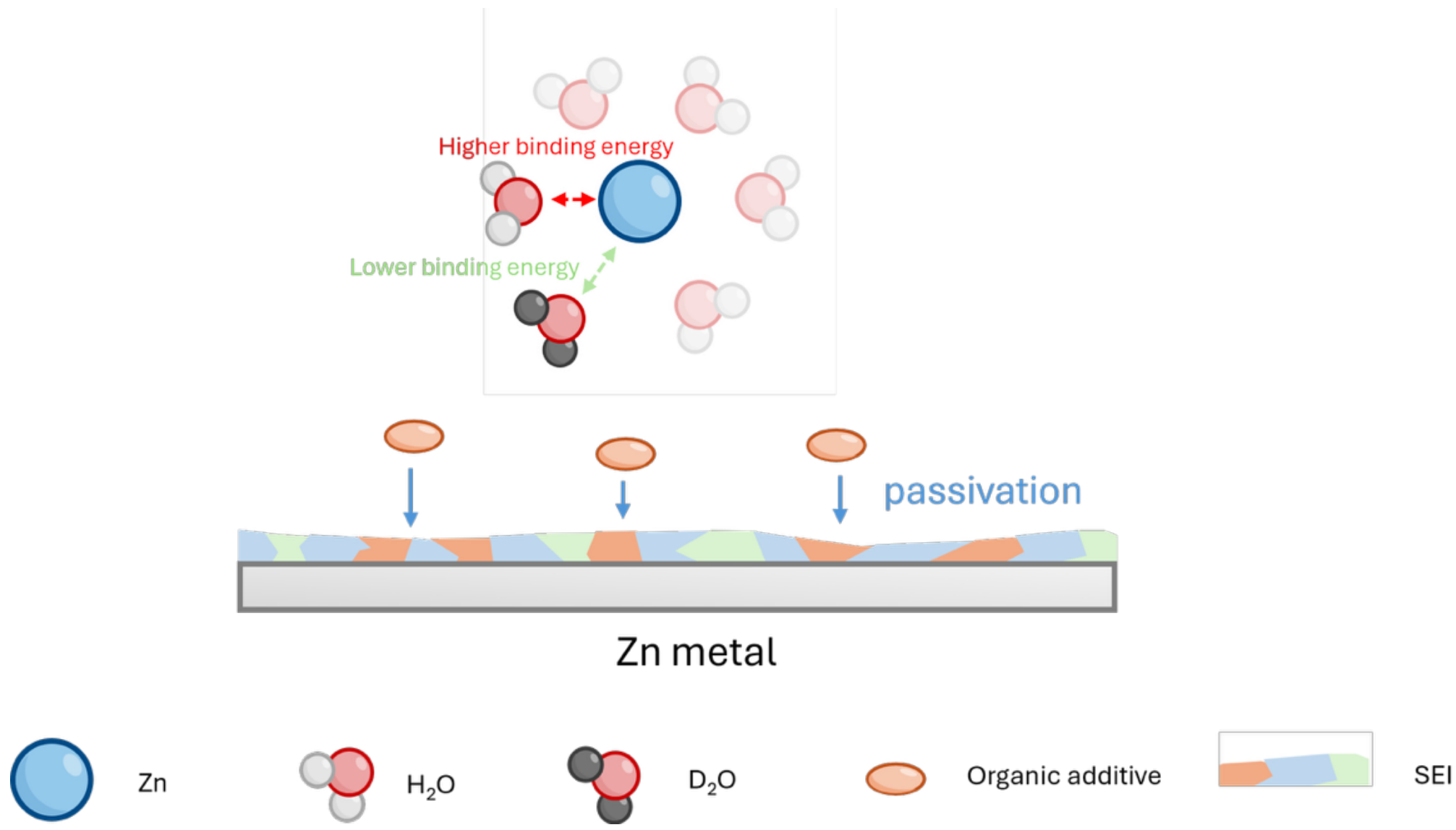

Supplementary Fig. 15. A schematic summarizing how concentration, isotopic substitution, and additive choice synergistically suppress solvated-water activity.

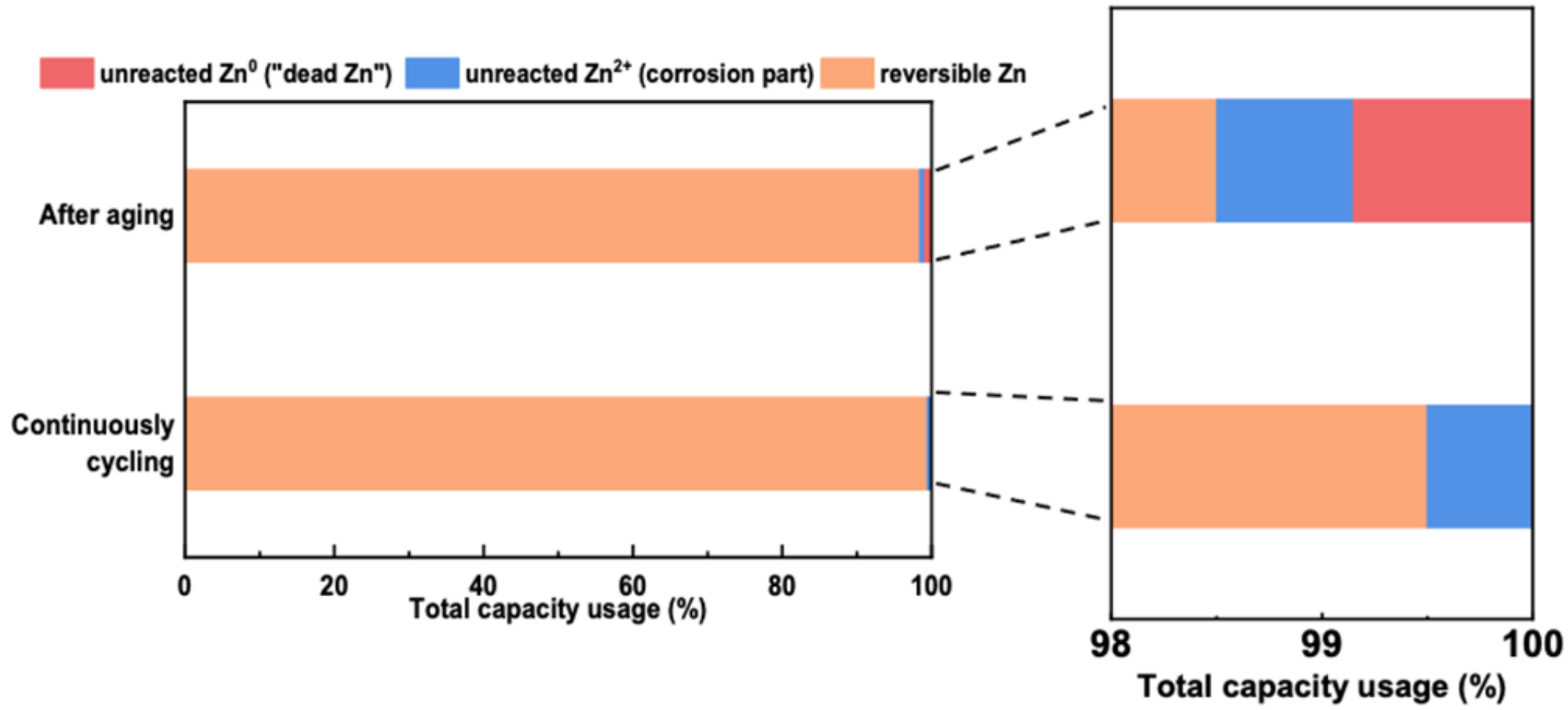

Supplementary Fig. 16. TGC Quantification of inactive Zn species left after stripping by the TGC method with 1Z5L electrolyte under 1 mA cm^{-2} and 1 mAh cm^{-2} on graphite paper substrate. The percentage of unreacted Zn^0, unreacted Zn^{2+}, and reversible Zn. Source data are provided as a Source Data file.

The aging sample was collected after undergoing one cycle followed by a 24-hour aging protocol. The continuously cycled sample was obtained after completing two consecutive cycles.

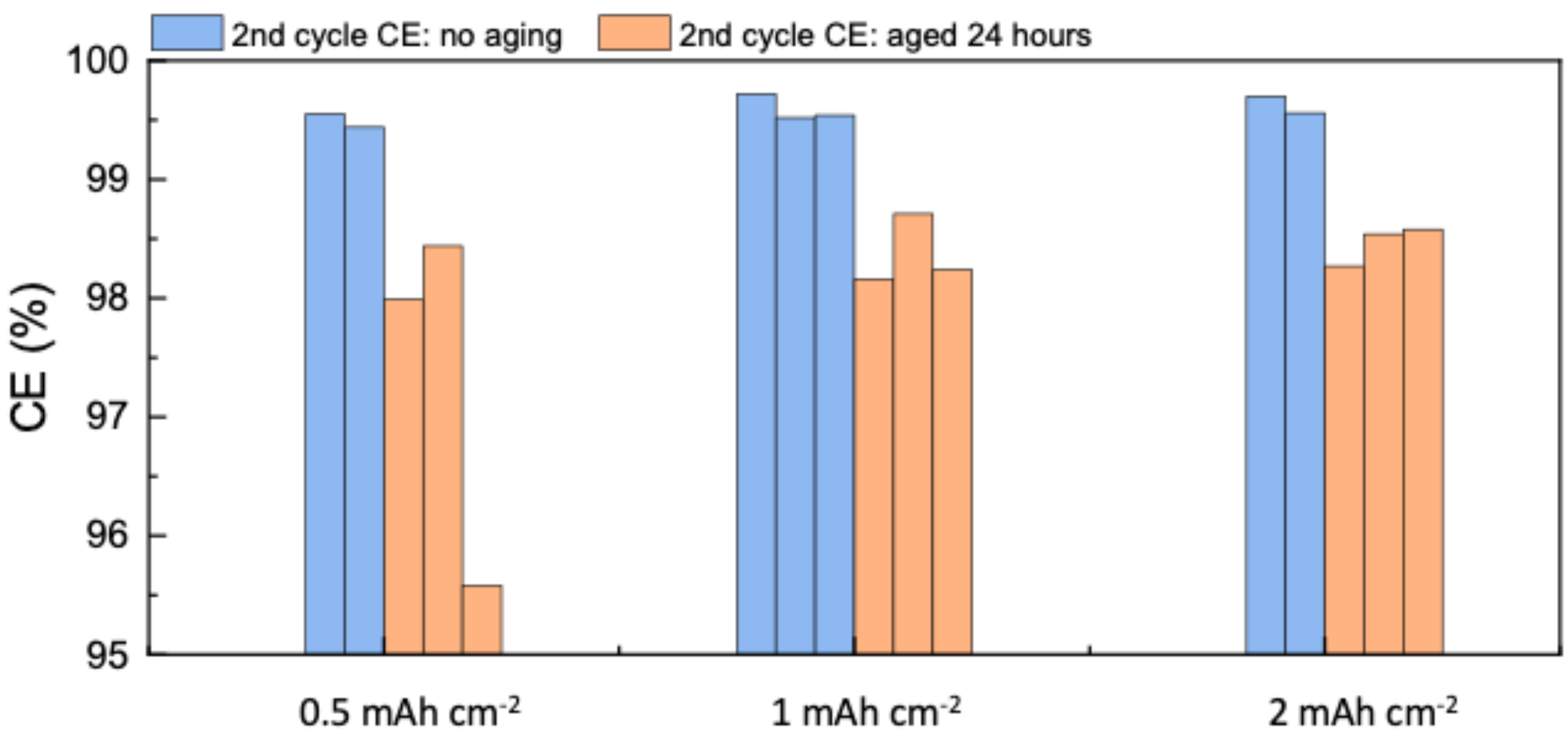

Supplementary Fig. 17. Comprehensive analysis of calendar aging performance. CE without aging (blue bar) and CE after 24 h aging (orange bar) under varying Zn deposition capacities. Source data are provided as a Source Data file.

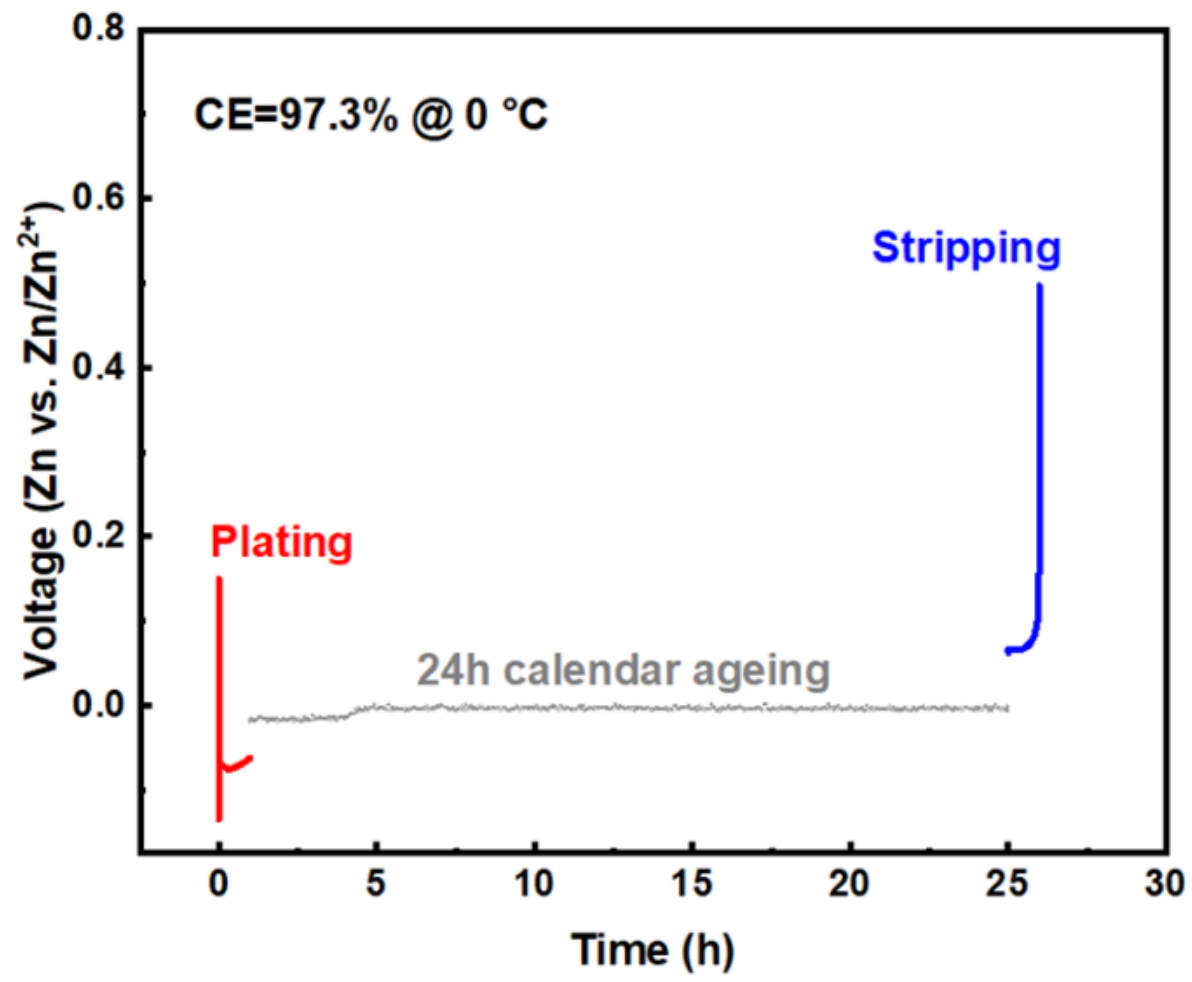

Supplementary Fig. 18. Voltage profile and capacity retention at 0 ºC at 1 mA cm^{-2}. Source data are provided as a Source Data file.

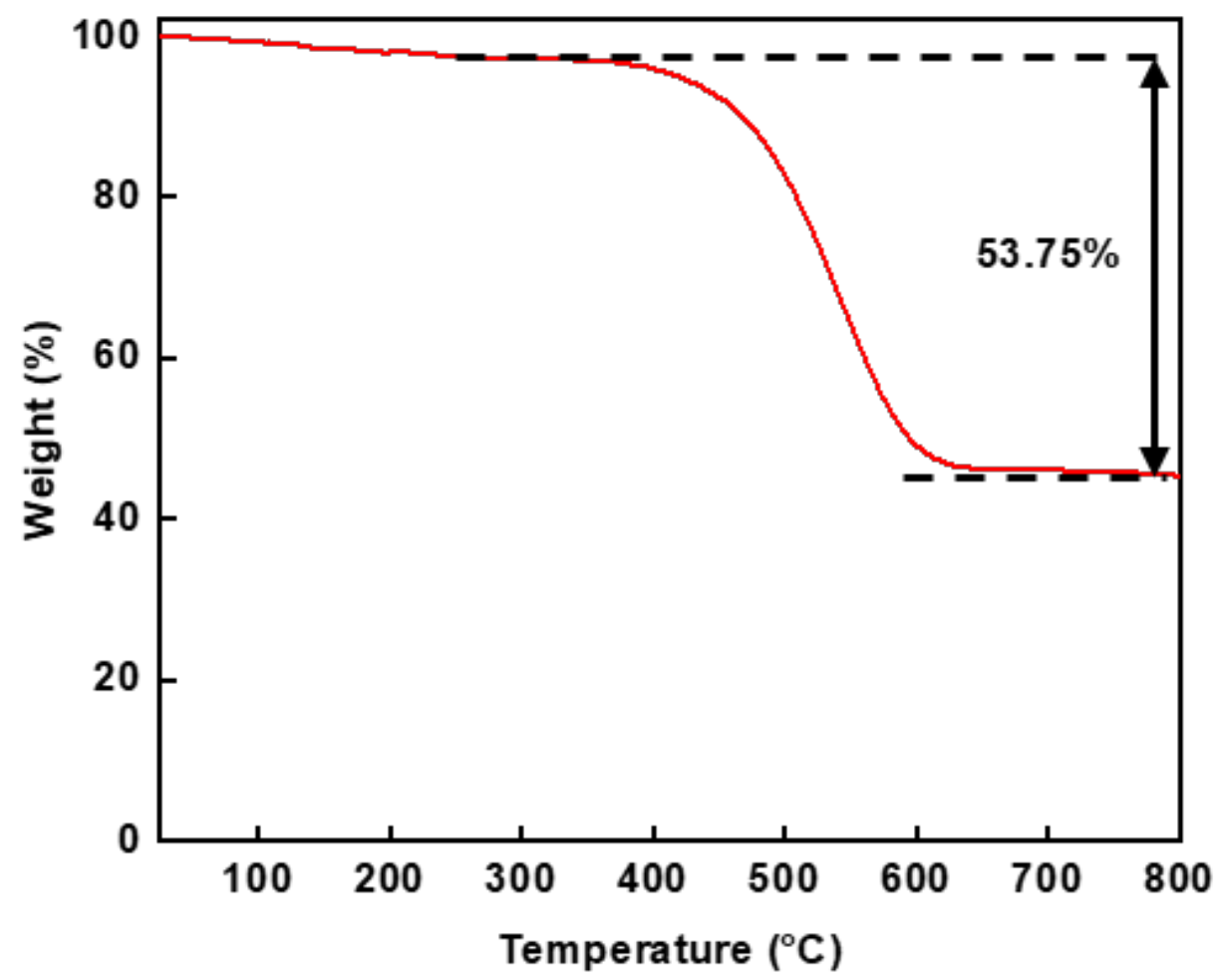

Supplementary Fig. 19. Characterizations of the as-prepared ZMO/C cathode material. TG analysis. Source data are provided as a Source Data file.

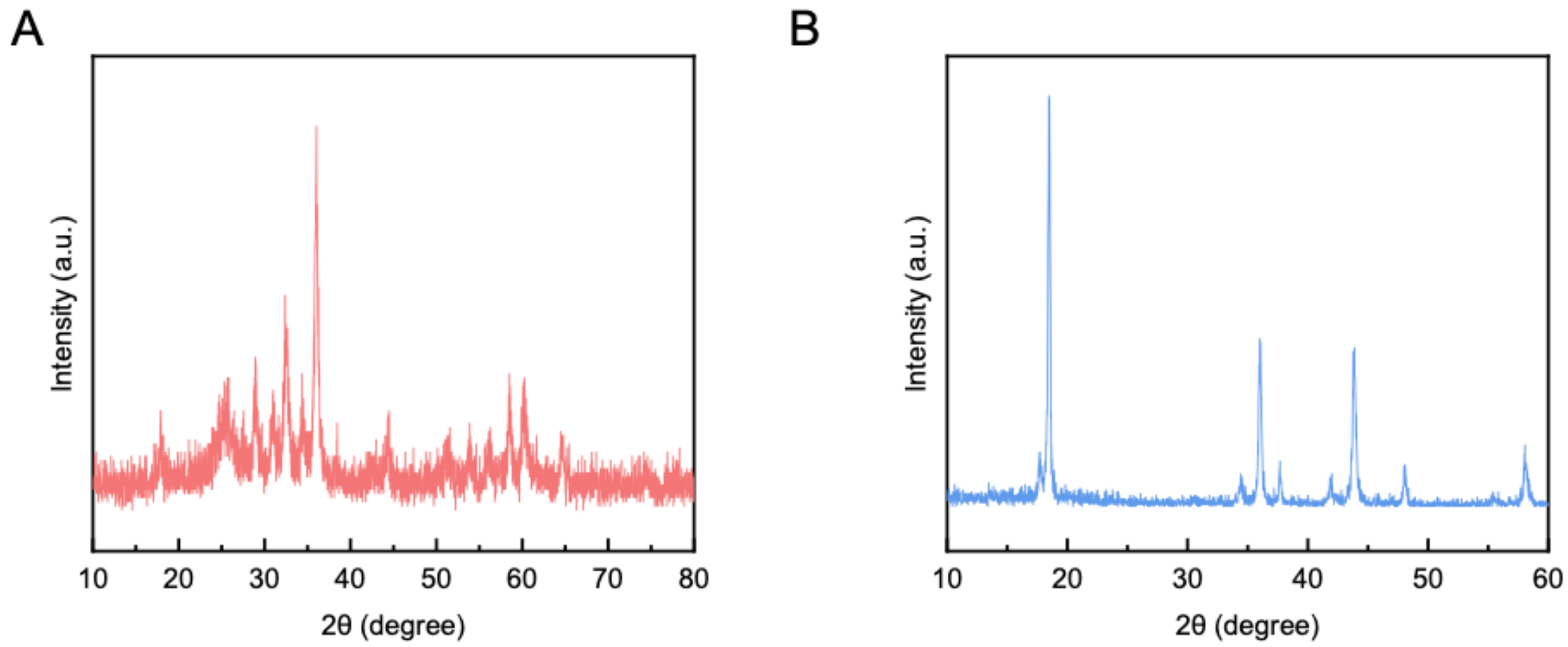

Supplementary Fig. 20. XRD patterns of different cathode materials. (A) $ZnMn_2O_4$ (ZMO). (B) $LiMn_2O_4$ (LMO). Source data are provided as a Source Data file.

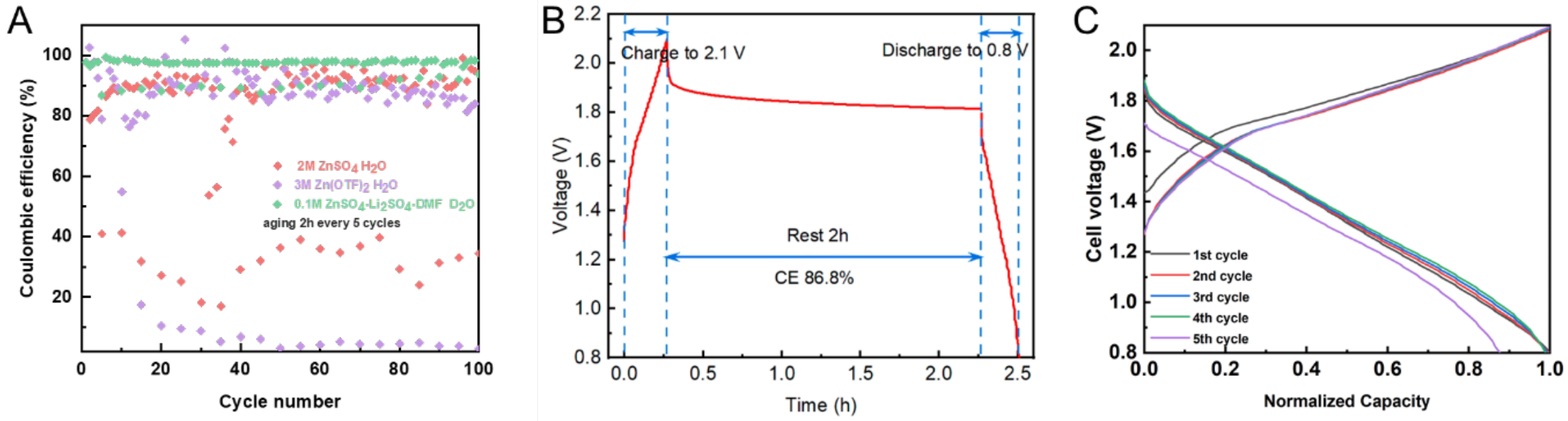

Supplementary Fig. 21. (A) CE of Graphite paper||ZMO anode-free cell in various electrolytes, (B) Voltage vs. time profile, and (C) voltage vs. capacity profile of Graphite paper||ZMO anode-free cell in our optimized electrolyte during calendar aging. Source data are provided as a Source Data file. Batteries are cycled at 0.5 mA cm^{-2}.

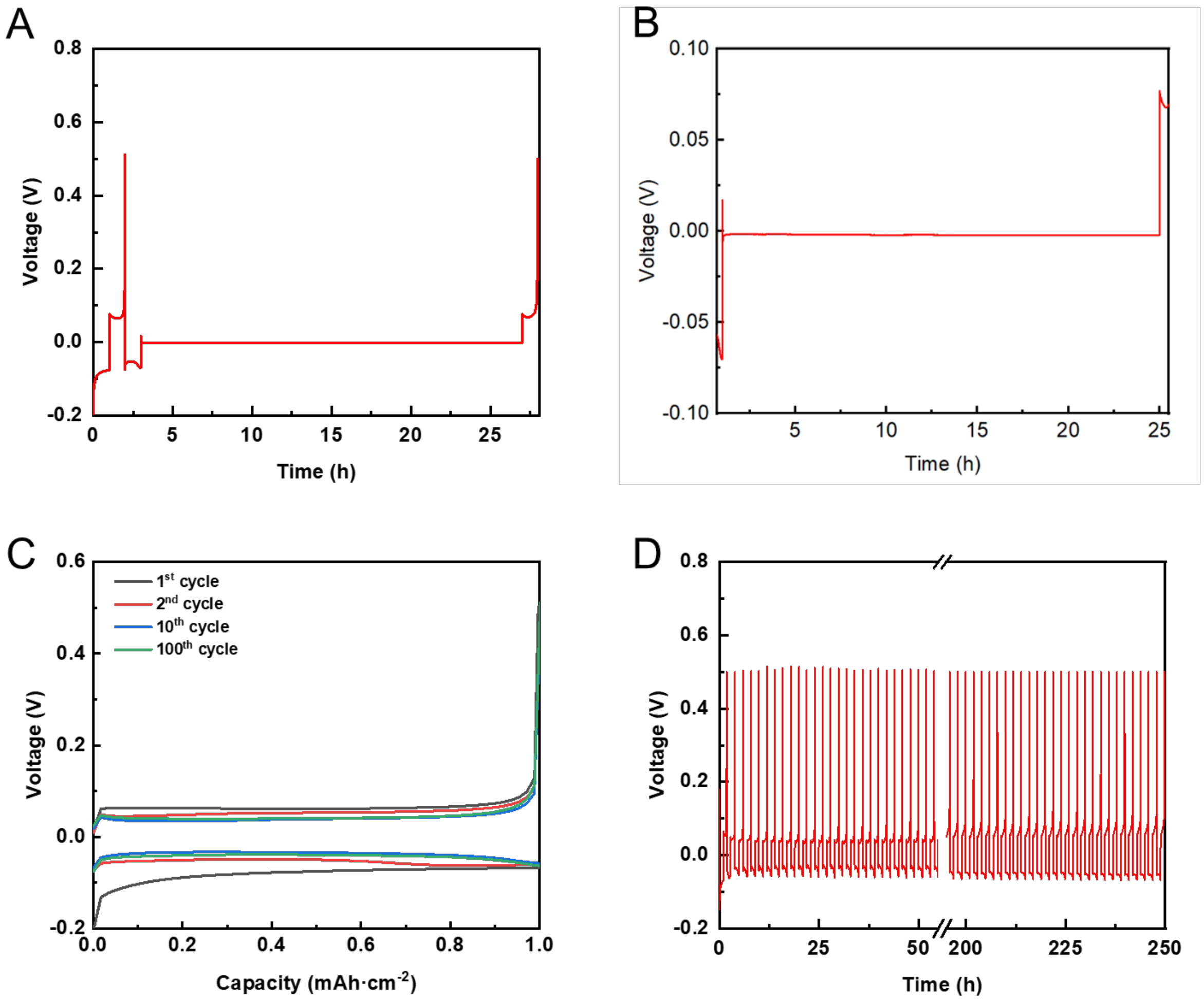

Supplementary Fig. 22. Voltage profiles of 1 mAh cm^{-2} plated Zn metal on graphite paper substrate with current density 1 mA cm^{-2}. (A-B) Voltage profile with 24 h calendar aging. (C) Voltage profile without calendar aging. (D) Overall voltage profile. Source data are provided as a Source Data file.

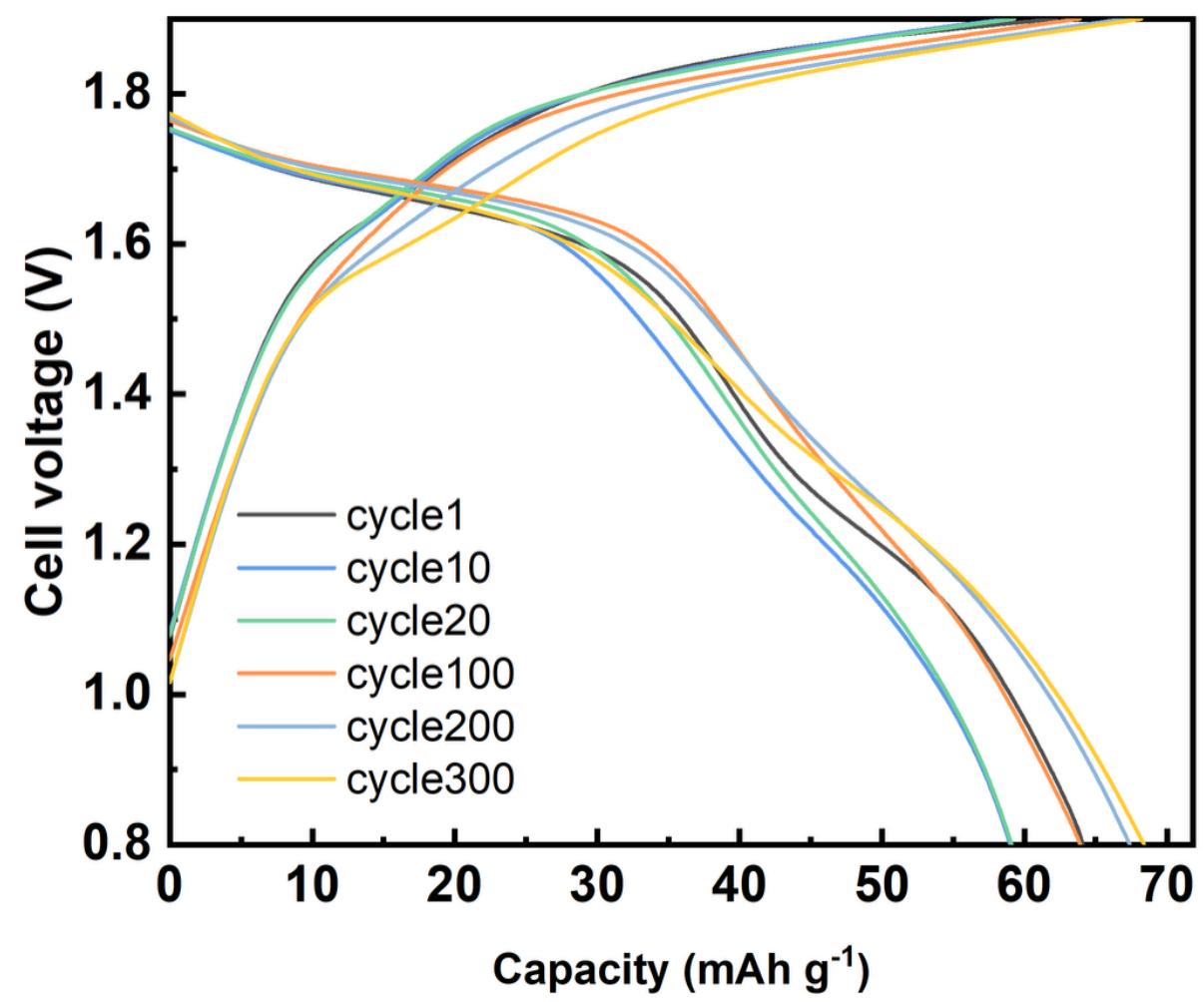

Supplementary Fig. 23. Voltage profiles of Zn||LMO full cell at a 4 C charge/discharge rate. Source data are provided as a Source Data file.

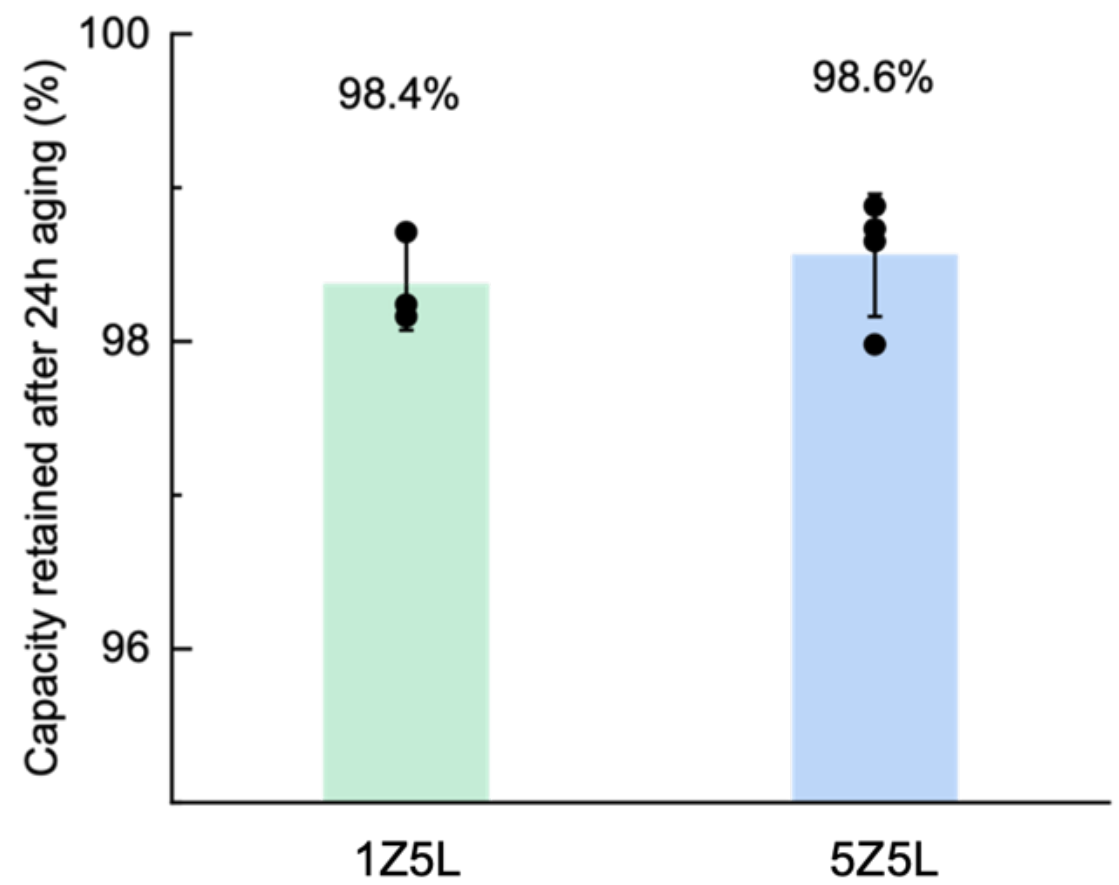

Supplementary Fig. 24. Capacity retained comparison after 24 h calendar aging with 1Z5L, where 0.5 M $ZnSO_4$ + 0.5 M Li_2SO_4 + 5 vol% DMF is denoted as 5Z5L. Error bars on plots show the standard deviation of 3 independent samples. Source data are provided as a Source Data file.

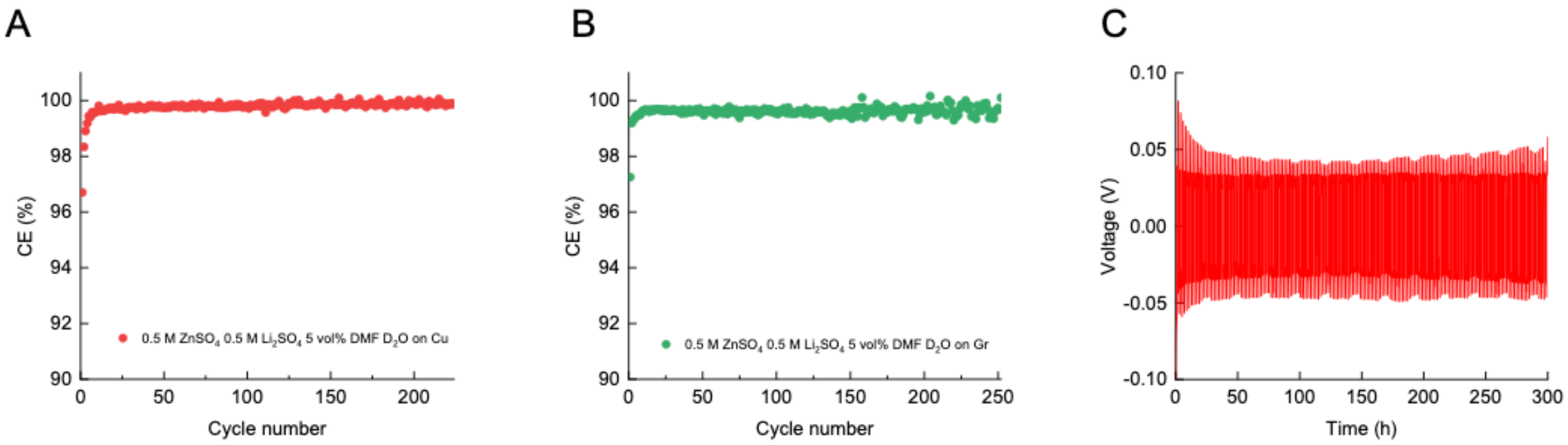

Supplementary Fig. 25. Comprehensive analysis of 5Z5L. (A) CE performance at 1 mA cm^{-2} and 1 mAh cm^{-2} using Cu foil as substrate. (B) CE performance at 1 mA cm^{-2} and 1 mAh cm^{-2} using graphite paper as substrate. (C) Zn plating/stripping stability test at 1 mA cm^{-2} and 1 mAh cm^{-2}. Source data are provided as a Source Data file.

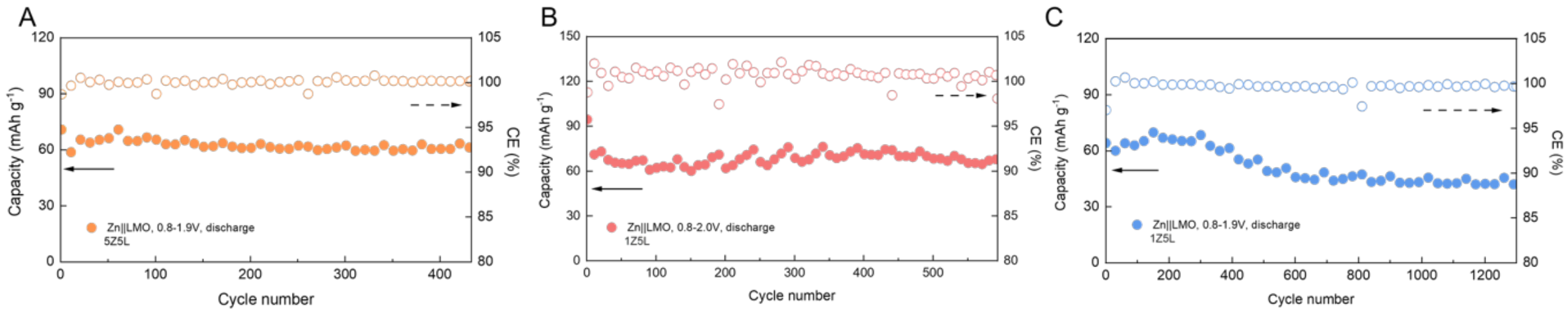

Supplementary Fig. 26. Zn||LMO hybrid cell cycling performance under different conditions. (A) Cycling performance of the Zn||LMO full cell using 5Z5L with cut-off voltage 0.8-1.9 V at a 2 C charge/discharge rate. (B) Cycling performance of the Zn||LMO full cell using 1Z5L electrolyte with cut-off voltage 0.8-2.0 V at a 2.8 C charge/discharge rate. (C) Cycling performance of the Zn||LMO full cell using with cut-off voltage 0.8-1.9 V at a 4 C charge/discharge rate. Source data are provided as a Source Data file.

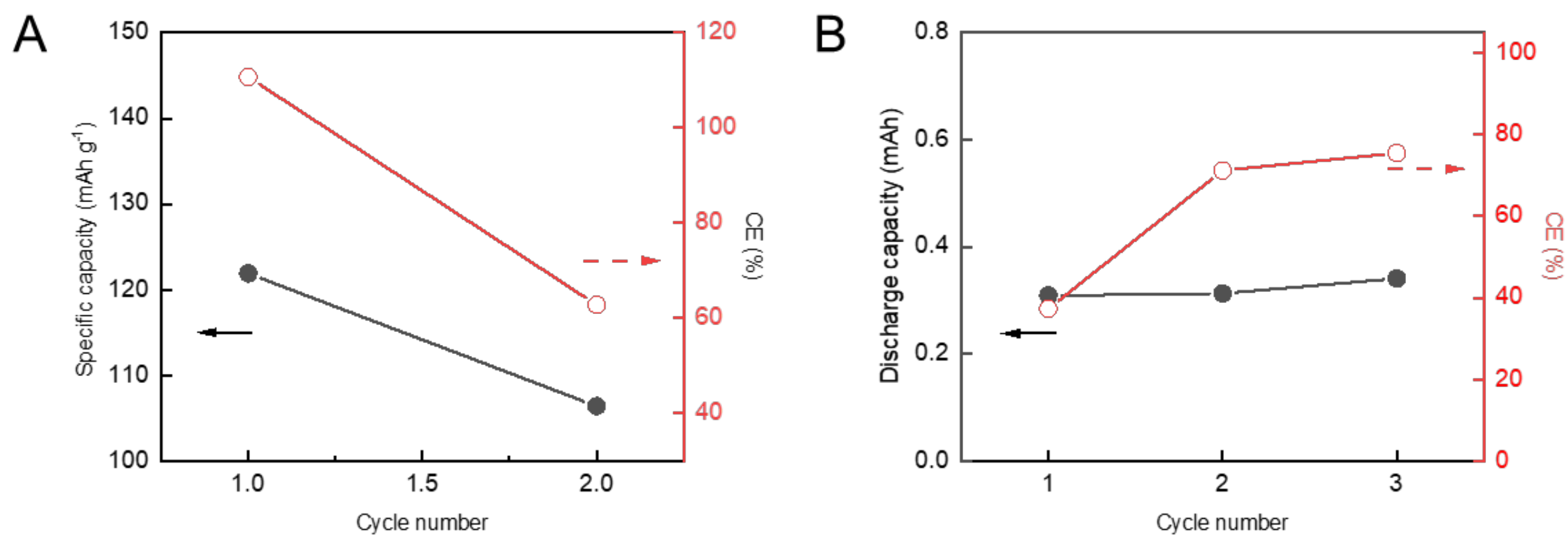

Supplementary Fig. 27. Batteries' formation cycles performance. (A) 2 formation cycles of Zn||LMO full cell using 1Z5L electrolyte with cut-off voltage 0.8-1.9 V at a 0.3 C charge/discharge rate. (B) 3 formation cycles of anode free cell using ZMO cathode with cut-off voltage 0.8-2.1 V at 0.05 mA cm^{-2} current density. Source data are provided as a Source Data file.

Supplementary Tables

Supplementary Table 1. Free energy of complexes in water solvent relative to $Zn(H_2O)_6$ and $Li(H_2O)_4$ respectively.[7]

Complex	ΔG (kcal/mol)
$Zn(H_2O)_6$	0
$Zn(H_2O)_5(H_2O)$	-0.724
$Li(H_2O)_4$	0
$Li(H_2O)_3(H_2O)$	-0.543

Supplementary Table 2. All fitted slopes and the calculated transfer coefficients from Tafel plots.

	Anodic reaction ($Zn \rightarrow Zn^{2+} + 2e^-$)	Cathodic reaction ($2H^+ + 2e^- \rightarrow H_2$)
Tafel slope (mV dec^{-1})	227	111
Transfer coefficient	0.13	0.53

The transfer coefficients (α) were calculated using the Tafel form derived from Butler–Volmer kinetics as follows:

$$\alpha = \frac{2.303RT}{nFb}$$

Where R is gas constant (8.314 J mol^{-1}), T=298 K at 25 °C, n is number of electrons involved in the reaction step associated with the respective Tafel branch, F is the Faraday constant (F=96485 C mol^{-1}), and b is Tafel plot derived by fitting the linear Tafel regions of the anodic and cathodic branches.

Supplementary Table 3. d-spacings with uncertainty measured from three SAED patterns in Supplementary Fig. 11 and the standard-referenced values.

Lattice plane (h k l)	d-spacing (Å, with uncertainties)	Standard d-spacing (Å)
**(100)**	**2.86 ± 0.01**	**2.81**
**(002)**	**2.63 ± 0.02**	**2.60**
**(101)**	**2.44 ± 0.03**	**2.48**
**(102)**	**1.87 ± 0.02**	**1.91**
**(110)**	**1.58 ± 0.01**	**1.63**
**(103)**	**1.45 ± 0.01**	**1.48**
**(112)**	**1.34 ± 0.01**	**1.38**

Supplementary Table 4. The error between the raw and fitted data using the equivalent circuit inset in Supplementary Fig. 7G.

Electrolytes	State	R1 ± Std. error (Ω)	R2 ± Std. error (Ω)	ΔRct (Ω)
0.1 M $ZnSO_4$ in H_2O	Pre-aging	38.20 ± 0.19	3.50 ± 0.16	—
	Post-aging	41.90 ± 0.23	165.90 ± 1.33	162.4
0.1 M $ZnSO_4$ + 0.5 M Li_2SO_4 in H_2O	Pre-aging	8.10 ± 0.06	4.70 ± 0.07	—
	Post-aging	8.00 ± 0.03	164.90 ± 2.23	160.2
0.1 M $ZnSO_4$ + 0.5 M Li_2SO_4 + 5 vol% DMF in D_2O	Pre-aging	15.70 ± 0.10	4.77 ± 0.14	—
	Post-aging	26.40 ± 0.09	80.77 ± 1.78	76.0

References

1. Blanc, L.E., Kundu, D. & Nazar, L.F. Scientific Challenges for the Implementation of Zn-Ion Batteries. *Joule* **4**, 771-799 (2020).

2. Ma, L. et al. Realizing high zinc reversibility in rechargeable batteries. *Nat. Energy* **5**, 743-749 (2020).

3. Zhao, C.-X., Li, Z., Chen, B., Chen, F. & Wang, C. Self-adaptive electrolytes for fast-charging batteries. *Nature Energy* (2025).

4. Davies, D.M. et al. Combined economic and technological evaluation of battery energy storage for grid applications. *Nat. Energy* **4**, 42-50 (2018).

5. Chazalviel, J. Electrochemical aspects of the generation of ramified metallic electrodeposits. *Phys Rev A* **42**, 7355-7367 (1990).

6. Chaba, N., Neramittagapong, S., Neramittagapong, A. & Eua-Anant, N. Morphology study of zinc anode prepared by electroplating method for rechargeable Zn-MnO(2) battery. *Heliyon* **5**, e02681 (2019).

7. Liu, Y. et al. Rechargeable aqueous Zn-based energy storage devices. *Joule* **5**, 2845-2903 (2021).
