# Supplementary material for: Dilute electrolytes for suppressing metal anode corrosion during calendar aging and cycling in aqueous zinc batteries
Source: Nat Commun. 2026 Aug 6;17:9463. doi: 10.1038/s41467-026-75100-x (PMC13542140; doi:10.1038/s41467-026-75100-x)
Supplement: Supplementary file 2 [file 41467_2026_75100_MOESM2_ESM.pdf]

Description of Additional Supplementary Files

File Name: Supplementary Data 1
Description: Optimized structures are in structure.zip
