# Supplementary material for: Dilute electrolytes for suppressing metal anode corrosion during calendar aging and cycling in aqueous zinc batteries
Source: Nat Commun. 2026 Aug 6;17:9463. doi: 10.1038/s41467-026-75100-x (PMC13542140; doi:10.1038/s41467-026-75100-x)
Supplement: Supplementary file 3 [file 41467_2026_75100_MOESM4_ESM.pdf]

nature portfolio

Peer Review File

Dilute electrolytes for suppressing metal anode corrosion during calendar aging and cycling in aqueous zinc batteries

Corresponding Author: Professor Yuzhang Li

This file contains all reviewer reports in order by version, followed by all author rebuttals in order by version.

Attachments originally included by the reviewers as part of their assessment can be found at the end of this file.

Version 0:

Reviewer comments:

Reviewer #1

(Remarks to the Author)
This paper addresses a compelling and significant topic concerning the use of ultra-low concentration electrolytes to mitigate the corrosion of Zn metal. While these findings may appear to contradict certain previous reports, they are consistent with authentic experimental evidence and are further supported by our recent results (https://doi.org/10.1016/j.ensm.2025.104632). Therefore, I recommend its publication in Nature Communications. Below are several points for consideration:
1. How should the corrosion behavior of Zn at different salt concentrations be understood? Conventional understanding suggests that super-high concentrations suppress corrosion by reducing water activity. Do the proposed mechanisms in this study challenge established theories?
2. There is a prevailing view that Zn metal forms a solid electrolyte interphase (SEI), analogous to Li-metal or graphite anodes. However, a concentration of 0.1 M appears insufficient to facilitate a stable SEI on Zn. What is the author's opinion regarding the existence and nature of an SEI on Zn, and what evidence supports this view?
3. While the optimized 0.1 M concentration may suppress corrosion, how does it influence the electrochemical kinetics of the Zn metal anode, particularly under high current density conditions?
4. Ionic diffusion gradients are generally recognized as a key factor driving metallic dendrite formation. How do the morphological evolution and dominant mechanisms of Zn deposition vary with electrolyte concentration?
5. It would be insightful to evaluate the performance of a full Zn battery configuration, including various cathode chemistries, when employing the 0.1 M electrolyte concentration.

Reviewer #2

(Remarks to the Author)
The manuscript addresses a timely and practically significant problem in aqueous zinc ion battery research—the severe capacity loss of metal anodes during calendar aging. The authors propose an unconventional "ultra-dilute" electrolyte strategy (0.1 M $ZnSO_4$ + Li_2SO_4 + DMF in D_2O) as an alternative to the widely adopted high-concentration "water-in-salt" concept, claiming that corrosion is mainly driven by solvated rather than free water molecules. The study combines density-functional analysis, spectroscopy, and electrochemical testing and reports markedly improved capacity retention and cycling stability.
While the topic is relevant and potentially influential, the current work can be improved to meet the standard expected for publication in Nature Communications. The central mechanistic claim—that solvated water dominates corrosion—is not experimentally verified, the stability of the complex electrolyte are insufficiently characterized. The study's conceptual framework is appealing but currently more correlative than causal. Before acceptance, the authors should address the additional validation and mechanistic clarifications outlined below.

1. Mechanistic basis: "solvated water drives corrosion" lacks direct proof
The central hypothesis that solvated rather than free water molecules govern Zn corrosion is the conceptual foundation of the paper, yet it is supported solely by static DFT/COHP calculations and correlative electrochemistry. Actually, no experimental discrimination between solvated and free water species is provided. Without spectroscopic validation (e.g.,

operando Raman, 17O NMR, isotopic exchange kinetics, or neutron scattering), the claim remains speculative.
The assertion that "free water is not reactive" is overstated. Even if free water has higher HER overpotential, the corrosion process in Zn involves mixed chemical-electrochemical steps ($Zn + 2H_2O \rightarrow Zn(OH)_2 + H_2$), in which both solvated and bulk species participate.
The authors should consider complementing the DFT results with characterization (for example, Raman or FTIR) to track coordinated vs. free-water vibrational modes under potential control.

2. Validation of electrolyte composition and stability
The multi-component electrolyte ($ZnSO_4$ + Li_2SO_4 + DMF + D_2O) introduces several reactive species. Stability of each under operating conditions is not demonstrated. DMF is known to decompose reductively on Zn and Cu surfaces, forming amides, formates, and carbonate species. The hypothesized reaction leading to $ZnCO_3$ + ZnO lacks evidence.
Li_2SO_4 can alter ionic transport but may also precipitate as Li_2CO_3 or $Li_2Zn(SO_4)_2·xH_2O$ during extended cycling. In the manuscript, no post-cycling XRD or ICP-OES analysis of electrolyte residues is shown.
Therefore, I suggest that the authors consider incorporating additional technologies (such as XPS, ToF-SIMS, or ICP) to support the conclusion.

3. Electrochemical testing details should be provided
First, the aging protocol ("24 h rest after plating") lacks procedural details: OCV conditions, temperature control, and self-discharge should be monitored. Besides, electrolyte volume-to-electrode-area ratio is critical for ultra-dilute systems; it must be reported.

4. Absence of impedance and transport analysis
The claim that Li_2SO_4 addition "substantially decreases ionic transport resistance" is only qualitatively described. So, the authors should provide full Nyquist plots and fitted equivalent-circuit parameters before and after aging; Zn^{2+} transference numbers and apparent diffusion coefficients from GITT or EIS to quantify transport effects of dilution and isotopic substitution.
In addition, discussion of how 0.1 M Zn^{2+} maintains dendrite-free deposition without severe concentration polarization—contradictory to classical analysis, should also be included.

5. Terminology and claims
"Ultra-dilute" is an overstatement for 0.1 M $ZnSO_4$; typical aqueous Zn batteries operate in the 0.5–3 M range, so "low-concentration regime" would be more accurate.
Besides, the concluding statement that the design is "broadly applicable to Fe metal and Na-ion batteries" is speculative and should be deleted unless supporting data exist.

6. Besides, there are also some small problems:
(1)For example, The authors should clarify transport-limiting regime: Since ion conductivity is a critical trade-off in ultra-dilute systems, a brief discussion or table comparing the measured ionic conductivities of various electrolyte formulations (possibly from Supplementary Figure 3) would help contextualize the practical feasibility without new experiments.
(2)Add reference to broader aqueous systems: As mentioned above, there is no experimental evidence about the Na- or Fe-based batteries in the manuscript. If the authors want to prove the conceptual generality, they should consider mentioning analogous solvation-corrosion relationships in Na- or Fe-based aqueous chemistries in the Discussion (combined experimental results would be better). This would underscore the broad applicability of the 'solvated-water reactivity' principle.
(3)Optional visualization suggestion: A schematic summarizing how concentration, isotopic substitution, and additive choice synergistically suppress solvated-water activity could be added to the main text or Supplementary Information for readers' clarity. No additional experiments are needed.

Reviewer #3

(Remarks to the Author)
- Title: Ultra-dilute electrolytes for suppressing metal anode corrosion during calendar aging and cycling
- Overall comments: The study addresses calendar aging in Zn-anode batteries. The authors attribute Zn corrosion during rest to solvated water. They introduce an ultra-dilute electrolyte (0.1 M $ZnSO_4$), with optional isotopic solvent substitution and targeted additives. They report >10× lower calendar fade (<1.5% capacity loss after 24 h) while maintaining cycling durability (>3,300 cycles) and high average Coulombic efficiency (~99.8%). If validated, the work suggests electrolyte concentration reduction as a general, mechanistically grounded route to improve both shelf-life and cycling stability in metal-anode chemistries. However, major revisions are required to substantiate the mechanistic claims according to the following detailed comments:
1. More detailed analysis is required for the Tafel plots in Figure 2F. Mechanism (rds), transfer coefficient, etc.
2. Quantitative analysis of the ionic conductivity is required for the developed electrolytes in comparison with other conventional ones.
3. Regarding Figure 2D, more thorough 3-electrode cell analysis must be done for the cyclic voltammetry of reversible zinc deposition and dissolution, especially with respect to the Coulombic efficiency and overpotential.
4. While the concentration of Zn is reduced, the pH is raised. Both factors seems to contribute to the H_2O stability in the opposite ways. Quantitative comparison is necessary to determine the effects of O-H bond instability and the raise of HER onset potential by the lower Zn concentration.
5. It has been suggested that free water activity is an important factor for the HER and OER, as evidenced by the widening of the voltage stability window in the water-in-salt electrolytes. In this work, theoretical DFT calculation suggested weakened O-H bond by the Zn-coordination. How to reconcile the two contridictory factors? Quantification of the instabilization is

necessary to determine which is more important for diminishing HER.
6. Voltage profiles are to be represented in the main figures for the full cell operation of Fig. 4G and 5E.
7. To further substantiate Zn/ZnO coexistence, please include FFTs from the HRTEM ROIs, zone-axis identification, or simulated/standard FFT/diffraction overlays for wurtzite ZnO (P63mc) and hcp Zn (P63/mmc). Report the calibrated _d_-spacings with associated uncertainties used for phase indexing. Additionally, several figure panels and their captions appear mismatched in Figure 3, please reconcile panel labels and descriptions for consistency. Also, standardize the chemical notation by rewriting Li2SO4 with true subscripts (rather than pasted Unicode numerals) and applied this formatting consistently across the manuscript.
8. The significant S signal in the EDS maps (Fig. S9) suggests the possible formation of basic zinc sulfate (ZHS; Zn4SO4(OH)6×_x_H2O). To substantiate the hypothesis, DMF decomposes to (HCON(CH3)2) reacts initially with the Zn to form ZnCO3 and ZnO on the electrode surface, please provide surface-analytical evidence (XPS/ToF-SIMS/Raman) to distinguish ZHS from ZnCO3/ZnO and to confirm DMF-derived fragments within the interphase. What is the chemical identity, thickness evolution, and ion-transport property (Zn2+ transference, conductivity) of the DMF-derived SEI during long rest?
9. Does the SEI mechanically survive storage? In-situ impedance during rest and post-rest morphology would clarify.
10. The pH rises as concentration falls, and capacity retention tracks pH. Is corrosion suppression pH-driven or solvation-driven? Please quantify how does the pH (bulk and interfacial) influence Zn2+ primary/secondary solvation structure, _e.g._, H-bonding signatures, and link to corrosion/HER metrics.
11. Because ultra-dilute electrolytes are prone to Zn2+ depletion and dendrite formation, it would strengthen the case to include operando imaging confirming uniform deposition at application-relevant current densities/areal capacities. Please show how Li2SO4 and the SEI shift these metrics.
12. Fig. 1A contains a typographical error: the term should read ‘water-in-salt’ electrolyte.
13. Regarding TGC analysis, more detailed information must be provided on the assumptions and formula to assign the measured signal to Zn0 and Zn2+ proportions.

Reviewer #4

(Remarks to the Author)
I co-reviewed this manuscript with one of the reviewers who provided the listed reports. This is part of the Nature Communications initiative to facilitate training in peer review and to provide appropriate recognition for Early Career Researchers who co-review manuscripts.

Reviewer #5

(Remarks to the Author)
This manuscript presents a strategy to significantly suppress capacity fade of Zn metal during static storage through the design of an ultra-low concentration ZnSO4 electrolyte (0.1 M), while maintaining excellent cycling performance. The work identifies solvated water molecules as the primary driver of corrosion and demonstrates that a combination of reduced salt concentration, isotopic solvent substitution, and targeted additives can simultaneously mitigate static decay and enhance cycling stability, offering a promising approach for long-lived metal-anode batteries. However, in view of the construction of electrolyte, there are still some shortcomings in the study of the mechanism of improving the performance of the manuscript in the comparison group and the specific components, and a major revision is required.
1. In order to clarify the enhancement of each component in the modified electrolyte system for corrosion resistance and aging mitigation, respectively, the reviewers suggested the authors to supplement the corresponding test results of the 2 M $ZnSO_4$+(0.5 M Li_2SO_4/5 vol% DMF in D_2O)-H_2O system.
2. The authors tested the capacity retention of the modified electrolyte system at 4 °C, 25 °C & 45 °C respectively. It is worth noting that due to the differences in inter/intramolecular bonding behaviour within the electrolyte at different temperatures, different undesirable phenomena affecting ion transport can occur. The reviewer suggests adding the possible anti-aging behaviour of electrolyte systems below 4°C under conditions of ice crystal appearance. At the same time, a specific analysis of the modification of the electrolyte for the modulation of hydrogen bonding behaviour and dissolution sheaths at different temperatures should be added.
3. Undoubtedly, higher concentrations of metal salts are beneficial to enhance the ionic conductivity and ion mobility number of the system. Very low concentration of modified electrolyte system can slow down the negative electrode corrosion by reducing the hydrated H2O in the dissolved sheath, but it may also lead to a decrease in the number and kinetic performance of the transported Zn ions (as shown in Fig. 2F). The authors should address this issue by giving a basis for screening appropriate concentrations.
4. The authors concluded that Li2SO4 could further reduce Zn ion transport resistance through solvent sheath modulation, and notably the addition of a high concentration of heterogeneous cationic support salts was shown to construct a high-quality SEI layer and avoid dead Zn formation through, for example, electrostatic interactions, in addition to modulating solvation sheath behaviour (10.1038/s41893-025-01646-1; 10.1002/ adfm.202514585). The reviewers suggest further testing and analysis for possible differences in the mechanism of action of this work at very low addition concentrations compared to traditional high concentration double salts. Specifically, a variety of tests including zinc ion mobility and reaction activation energies for different systems would help to elucidate the mechanism of modification.
5. The manuscript suggests that an ultra-low concentration electrolyte system depleted of solubilised water would be beneficial in preventing negative electrode corrosion, however this needs to be demonstrated more. Compositional distribution and morphological analysis of fully aged electrodes would be more effective in confirming this view. In addition, the reviewer believes that the ultra-low concentration electrolyte system may have a special effect on the transport and de-embedding of zinc ions at the electrode-electrolyte interface, and suggests that further analysis of the interfacial behaviour should be carried out.

6. “This nanostructured SEI, rich in … Zn deposition by minimizing uncontrolled side reactions.” Are there any specific data, such as ion transference numbers or diffusion coefficients, that demonstrate the SEI film actively promotes Zn2+ migration?

Version 1:

Reviewer comments:

Reviewer #1

(Remarks to the Author)
The revised manuscript has well addressed the concerns and I have no extra question.

Reviewer #2

(Remarks to the Author)
The manuscript is well-revised and can be accepted now.

Reviewer #3

(Remarks to the Author)
The authors addressed all my review comments. I think it is suitable for publication. However, in my opinion, the authors may target to increase CE for the future direction.

Reviewer #4

(Remarks to the Author)
I co-reviewed this manuscript with one of the reviewers who provided the listed reports. This is part of the Nature Communications initiative to facilitate training in peer review and to provide appropriate recognition for Early Career Researchers who co-review manuscripts.

Reviewer #5

(Remarks to the Author)
The authors have properly addressed all my comments. I believe the manuscript is ready for publication.

Open Access This Peer Review File is licensed under a Creative Commons Attribution 4.0 International License, which permits use, sharing, adaptation, distribution and reproduction in any medium or format, as long as you give appropriate credit to the original author(s) and the source, provide a link to the Creative Commons license, and indicate if changes were made.
In cases where reviewers are anonymous, credit should be given to 'Anonymous Referee' and the source.
The images or other third party material in this Peer Review File are included in the article’s Creative Commons license,

unless indicated otherwise in a credit line to the material. If material is not included in the article's Creative Commons license and your intended use is not permitted by statutory regulation or exceeds the permitted use, you will need to obtain permission directly from the copyright holder.
To view a copy of this license, visit https://creativecommons.org/licenses/by/4.0/

Response Letter to Reviewers' Comments

Manuscript ID: NCOMMS-25-74602

Title: Ultra-dilute electrolytes for suppressing metal anode corrosion during calendar aging and cycling

Authors: Haoyang Wu, Bo Liu, Dingyi Zhao, Dongfang Cheng, Keyue Liang, Xintong Yuan, Kaixi Chen, Min-Ho Kim, Kaiyan Liang, Jung Tae Kim, Jiayi Yu, Tian-Yu Wang, Philippe Sautet, and Yuzhang Li

Table R1. New additional experiments performed during the revision process.

New experiments	Implications	Corresponding comments
Zn‖Zn symmetric-cell measurements at 5 mA cm^{-2}	Zn anode kinetics at high current densities	R1-3
SEM characterization of Zn deposited in 2 M, 0.5 M, 0.1 M, and 0.01 M $ZnSO_4$ electrolytes	Morphological evolution of Zn deposition with different electrolyte concentrations	R1-4, R2-4
Full cells using a vanadium oxide cathode chemistry	Feasibility of our optimized electrolyte in a full Zn battery configuration	R1-5
Post-cycling XPS analysis on the surface	DMF-enabled interphase formation and decomposition products analysis	R2-2, R3-8
Post-cycling XRD analysis on the surface	Li_2SO_4 precipitation of Li_2CO_3 / $Li_2Zn(SO_4)_2 \cdot xH_2O$ concerns	R2-2, R3-8
EIS measurements with equivalent circuit fitting for different representative electrolytes before and after 24 h calendar aging	Quantification of transport effects of dilution and isotopic substitution	R2-4, R3-8, R3-9, R5-1, R5-5

Apparent transference number ($t_{+,app}$) measurement in Zn‖Zn symmetric cells across different electrolyte formulations	Quantification of transport effects of dilution and isotopic substitution	R2-4, R3-8, R5-6
Ionic conductivities of various electrolyte formulations with co-solvent	Contextualization of the practical feasibility	R2-6
Ionic conductivity of other conventional ones (2 M $ZnSO_4$ and 3 M $Zn(OTf)_2$)	General comparison	R3-2
SEM, EDS, and TEM before and after aging	Chemical identity and SEI thickness evolution during long rest	R3-8, R3-9, R5-5
FIB-SEM after deposition at 20 mA/cm^2 and 3 mAh/cm^2 at different electrolytes	Operando imaging confirming uniform deposition at application-relevant current densities/areal capacities to show how Li_2SO_4 and the SEI shift these metrics	R3-11
Aging performance of different electrolytes	Clarification of each component enhancement in the modified electrolyte system for corrosion resistance and aging mitigation	R5-1
Aging performance at different temperatures	Anti-aging behavior below 4°C under conditions of ice crystal appearance to study the hydrogen bonding behavior and dissolution sheaths	R5-2
Ionic conductivity of 0.01 M $ZnSO_4$ in H_2O	Giving a basis for screening appropriate concentrations	R5-3

Ionic conductivity of different electrolytes at different temperatures	Zinc ion mobility and reaction activation energies analysis for different systems	R5-4

Response to Reviewer #1:

This paper addresses a compelling and significant topic concerning the use of ultra-low concentration electrolytes to mitigate the corrosion of Zn metal. While these findings may appear to contradict certain previous reports, they are consistent with authentic experimental evidence and are further supported by our recent results (https://doi.org/10.1016/j.ensm.2025.104632). Therefore, I recommend its publication in Nature Communications. Below are several points for consideration:

Authors' Response: We sincerely thank Reviewer #1 for the positive assessment of our work and for highlighting the importance of using ultra-low-concentration electrolytes to mitigate Zn corrosion, as supported by prior work *[Energy Storage Mater., 2025, 82, 104632]*. We greatly appreciate the opportunity to revise our manuscript and address the concerns raised.

1. How should the corrosion behavior of Zn at different salt concentrations be understood? Conventional understanding suggests that super-high concentrations suppress corrosion by reducing water activity. Do the proposed mechanisms in this study challenge established theories?

Authors' Response: Our proposed mechanism does not challenge the established concept that reducing water activity and weakening Zn–H_2O interactions can suppress corrosion. Instead, **it complements it by clarifying that the key corrosive population is the water molecules coordinated to Zn^{2+} (i.e., "solvated water"), which can be minimized via two seemingly opposite but mechanistically aligned electrolyte-design routes**.

First, in high-concentration electrolytes including "water-in-salt" regimes, increasing salt content both reduces the amount of available water and promotes anion-rich Zn^{2+} solvation, thereby displacing water from the primary Zn^{2+} solvation shell and reducing water-driven parasitic reactions. This framework is consistent with prior mechanistic analyses showing suppressed water reactivity at high salt concentrations due to reduced water activity and decreased Zn^{2+} hydration, as well as broader solvation-regulation strategies in which replacing water in the Zn^{2+} solvation shell mitigates side reactions *[Science 2015, 350, 938-943, Chem. Mater. 2025, 37, 20, 8364-8382; Nat. Commun. 2024, 15, 6249]*.

Second, our ultra-low-concentration electrolyte mitigates corrosion by targeting the same mechanism, thereby minimizing the absolute population of Zn^{2+}-coordinated water by lowering Zn^{2+} concentration, so that most water remains uncoordinated (unsolvated) rather than bound in Zn^{2+} hydration shells. Importantly, our DFT results further support this unified picture by indicating that solvated water is intrinsically more corrosive than free water, consistent with prior fundamental studies showing that the local solvation environment and the strength of cation–water interactions can strongly modulate water interfacial reactivity *[J. Am. Chem. Soc. 2022, 144, 4, 1589-1602; Sci. Adv. 2025, 11, 30, eadx8413]*.

2. There is a prevailing view that Zn metal forms a solid electrolyte interphase (SEI), analogous to Li-metal or graphite anodes. However, a concentration of 0.1 M appears insufficient to facilitate a stable SEI on Zn. What is the author's opinion regarding the existence and nature of an SEI on Zn, and what evidence supports this view?

Authors' Response: We thank the reviewer for raising this important point about the SEI concept in aqueous Zn systems. We agree that, in the absence of functional additives, a conventional, stable SEI analogous to Li metal or graphite is generally not expected on Zn in mild aqueous electrolytes (e.g, 2 M $ZnSO_4$). Instead, Zn typically forms non-ideal passivation byproducts (e.g., $ZnO/Zn(OH)_2$ and basic zinc sulfate), which are often inhomogeneous and do not fully suppress ongoing parasitic reactions.

In our study, we therefore introduced a small amount of sacrificial additive (5 vol. % DMF) specifically to promote formation of a thin, conformal SEI-like interphase that improves interfacial robustness and Zn deposition morphology *[J. Alloys Compd. 2024, 972, 172773; Natl. Sci. Rev. 2022, 9, nwac051]*. We keep DMF at low concentration to minimize excessive decomposition while still enabling a beneficial interphase. Experimentally, we provide direct evidence that an interphase forms when DMF is included. TEM reveals a ~15 nm conformal layer on deposited Zn (Fig. 3). This additive-enabled interphase concept aligns with the broader aqueous-Zn literature, where stable cycling is often achieved via in situ-formed or artificially induced interphases induced by electrolyte additives (e.g., carbonaceous/organic–inorganic hybrids, F-rich layers, sulfide-rich layers) *[Nat. Commun. 2022, 13, 5348; ACS Energy Lett. 2024, 9, 3, 1000–1007]*.

3. While the optimized 0.1 M concentration may suppress corrosion, how does it influence the electrochemical kinetics of the Zn metal anode, particularly under high current density conditions?

<u>Authors' Response:</u> We thank the reviewer for raising the point regarding Zn anode kinetics at high current densities. While our study primarily focuses on corrosion suppression and calendar-aging mitigation enabled by an ultralow Zn-salt concentration, we agree that low Zn^{2+} concentration can introduce kinetic limitations (e.g., increased ohmic/concentration polarization) at high current densities due to reduced ionic conductivity and a greater propensity for cation depletion near the interface. Mechanistically, we emphasize that the potential kinetic penalty associated with dilute Zn^{2+} is mitigated in our formulation by incorporating a supporting salt, Li_2SO_4, to enhance ionic transport.

To directly evaluate Zn plating/stripping behavior under a higher current density, we performed additional Zn‖Zn symmetric-cell measurements at 5 mA cm^{-2} (Fig. R1). Under these harsher conditions, our ultralow-concentration electrolyte exhibits stable voltage profiles, whereas the 2 M $ZnSO_4$–H_2O electrolyte shows greater polarization and direct failure during cycling. These results indicate that our electrolyte design, despite being diluted in Zn^{2+}, can still sustain Zn plating/stripping at an acceptable current density. However, we also note that further increasing the rate (e.g., to 10 mA cm^{-2} at 1 mAh cm^{-2}) would likely require combining our concept with other established high-rate enabling strategies *[Nat. Commun. 2023, 14, 641; Adv. Mater. 2023, 35, 2300620; Nat. Commun. 2016, 7, 11801]*, which will be an important direction for future work.

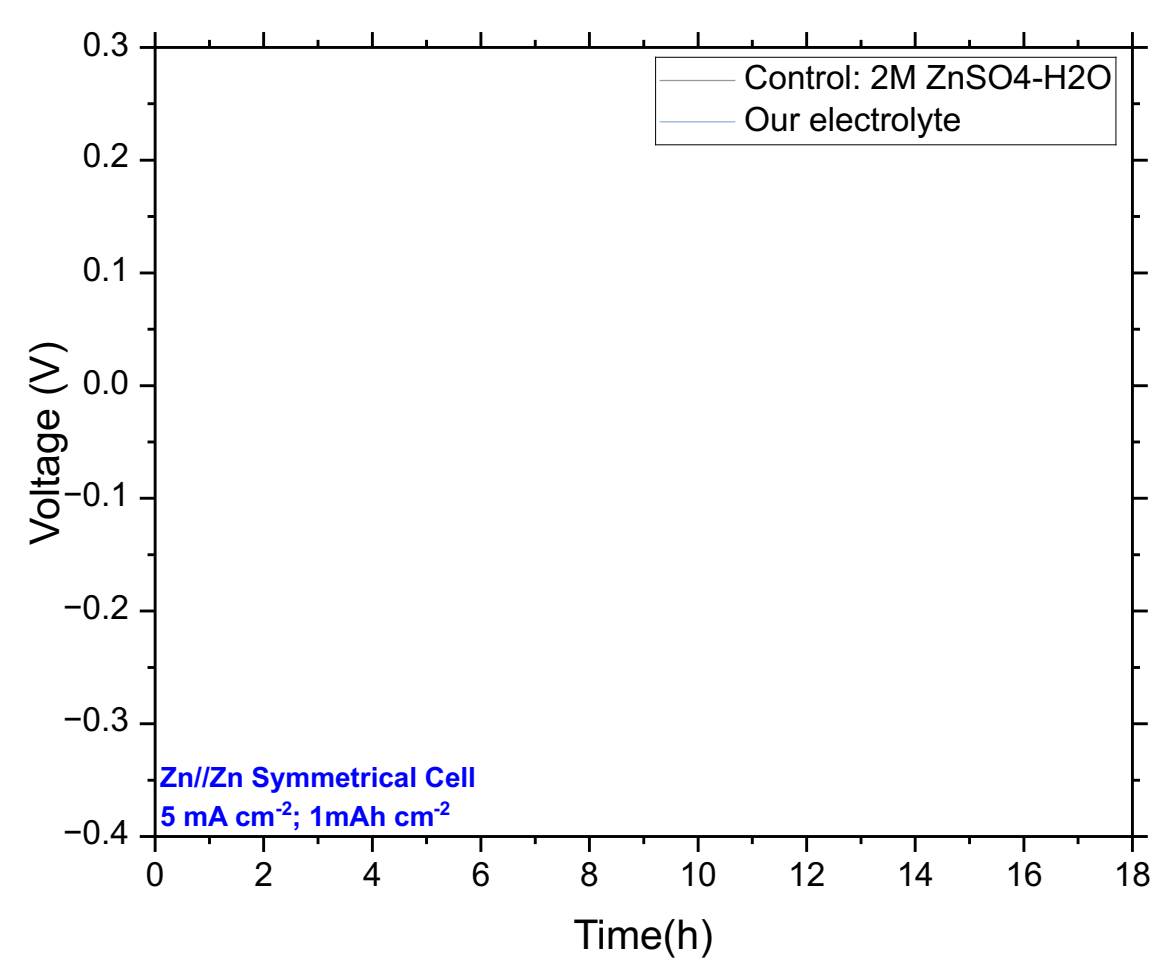

Fig. R1 Zn‖Zn symmetric-cell measurements at 5 mA cm^{-2}.

4. Ionic diffusion gradients are generally recognized as a key factor driving metallic dendrite formation. How do the morphological evolution and dominant mechanisms of Zn deposition vary with electrolyte concentration?

Authors' Response: We agree that ion-transport limitations and the associated concentration polarization are key factors for non-uniform metal growth with mossy/dendritic morphologies. In classical electrodeposition theory, lowering the salt concentration shortens the transition time to interfacial ion depletion, often discussed in terms of Sand's time, thereby increasing the propensity for dendritic growth *[Phys. Rev. A 1990, 42, 12, 7355-7367; J. Electrochem. Soc. 2006, 153, 5, C357-C364]*.

To address the reviewer's comment, we performed additional SEM characterization of Zn deposited at 1 mA cm^{-2} and 1 mAh cm^{-2} in 2 M, 0.5 M, 0.1 M, and 0.01 M $ZnSO_4$ in H_2O electrolytes. Consistent with classic analyses and prior Zn electrodeposition studies *[Heliyon 2019, 5, 10, e02681; J. Electrochem. Soc. 2006, 153, 5, C357-C364]*, we observe a clear concentration-dependent morphological evolution, where higher concentrations yield orderly, closely packed deposits, whereas lower concentrations lead to increasingly clustered and ultimately mossy and dendritic features, indicative of stronger mass-transport control and interfacial depletion (Supplementary Fig. 9).

Importantly, we do not claim that low $ZnSO_4$ concentration alone guarantees dendrite-free Zn deposition. Instead, our study shows that reducing Zn^{2+} concentration minimizes the formation of *corrosive solvated water*, which drives calendar-aging stability, while dendrite suppression during cycling requires a complementary interfacial and morphology-regulation strategy. In our optimized electrolyte, this is achieved by combining:

- Supporting salt (Li_2SO_4) to improve ionic conductivity and mitigate concentration polarization.
- DMF additive to promote in situ formation of a conformal interphase that regulates Zn nucleation/growth toward compact morphologies *[Angew. Chem. Int. Ed. 2025, 64, 16, e202501359; Nat. Commun. 2024, 15, 3914; Joule 2022, 6, 5, 1103-1120; ACS Energy Lett. 2023, 8, 3, 1613-1625]*.

- Isotope substitution (D_2O) to reduce solvent activity or proton-donor reactivity (kinetic isotope effect), suppressing parasitic reactions that otherwise roughen the interface *[Angew. Chem. Int. Ed. 2023, 62, 16, e202300608; Angew. Chem. Int. Ed. 2022, 61, 25, e202203137]*.

Manuscript Edits:

Page S14:

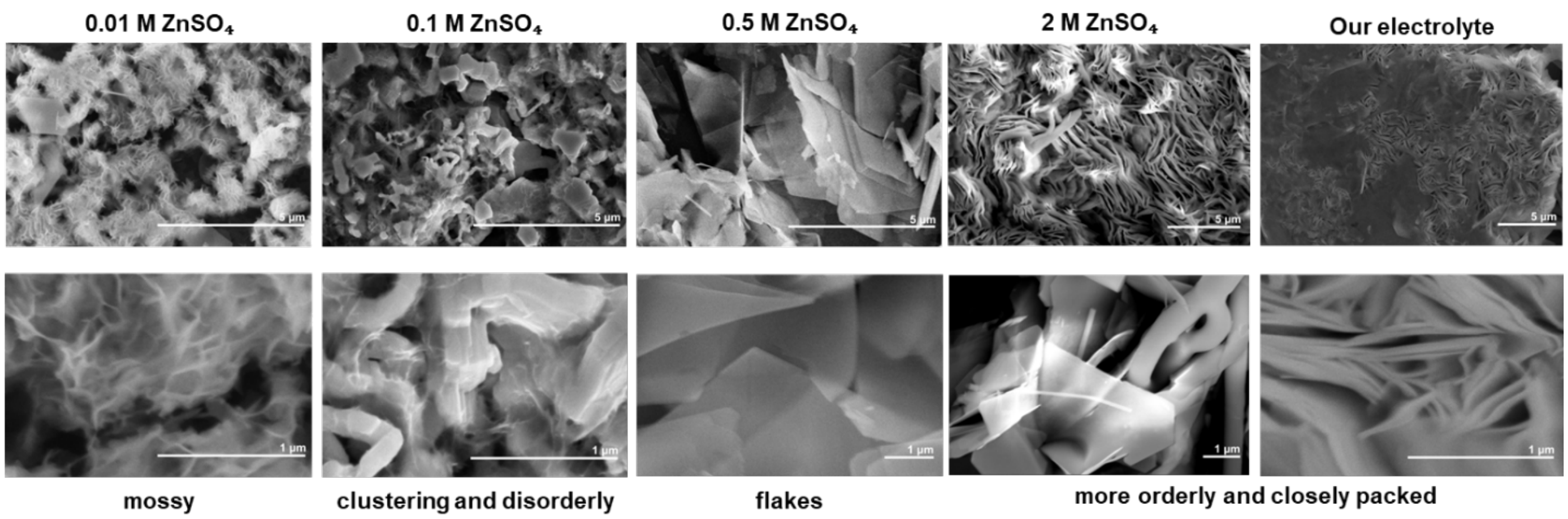

Supplementary Fig. 9. Zn deposition morphology at 1 mA cm^{-2} and 1 mAh cm^{-2} across different electrolytes.

In classical electrodeposition theory, lowering the salt concentration shortens the transition time to interfacial ion depletion, often discussed in terms of Sand's time, thereby increasing the propensity for dendritic growth.[3, 4] Consistent with classic analyses and prior Zn electrodeposition studies,[3, 5] we also observe a clear concentration-dependent morphological evolution, where higher concentrations yield orderly and closely packed deposits, whereas lower concentrations lead to increasingly clustered, and ultimately mossy and dendritic features, indicative of stronger mass-transport control and interfacial depletion (Supplementary Fig. 9). We clarify that low $ZnSO_4$ concentration alone does not guarantee dendrite-free Zn deposition. Instead, our central design principle is to reduce Zn^{2+} concentration, thereby minimizing corrosive solvated water (which drives calendar-aging stability), while dendrite suppression during cycling requires a complementary interfacial/morphological-regulation strategy.

5. It would be insightful to evaluate the performance of a full Zn battery configuration, including various cathode chemistries, when employing the 0.1 M electrolyte concentration.

Authors' Response: We assembled full cells using a vanadium oxide cathode chemistry (laser-scribed nano-vanadium oxide, LNVO) that we previously reported and mechanistically established to operate via mixed Zn^{2+}/H_3O^+ co-insertion in conventional aqueous 2 M $ZnSO_4$ electrolytes *[Adv. Mater. 2024, 36, 32, 2404796]*. With our optimized electrolyte, the LNVO/Zn full cell delivers a reversible capacity, approximately half that obtained in 2 M $ZnSO_4$, while maintaining ~80% capacity retention over 100 cycles (Fig. R2). The reduced capacity is primarily a consequence of suppressing H_3O^+-facilitated charge storage in the ultra-dilute formulation where D_2O is used as the solvent, consistent with our prior mechanistic understanding that hydronium co-insertion can contribute a fraction of capacity and enable faster kinetics in many vanadium oxide cathodes *[Adv. Mater. 2024, 36, 32, 2404796; ACS Energy Lett. 2020, 5, 9, 2979-2986; Acc. Chem. Res. 2024, 57, 19, 2887-2900; Nat. Commun. 2023, 14, 3067]*. Importantly, these full-cell data support the *feasibility* of our optimized electrolyte in a full Zn battery configuration, while a comprehensive screening study across additional cathode families (e.g., MnO_2 polymorphs, Prussian blue analogs, organic cathodes) to clarify which cathode classes are most likely to be compatible with ultra-dilute electrolytes would still be valuable for our future work.

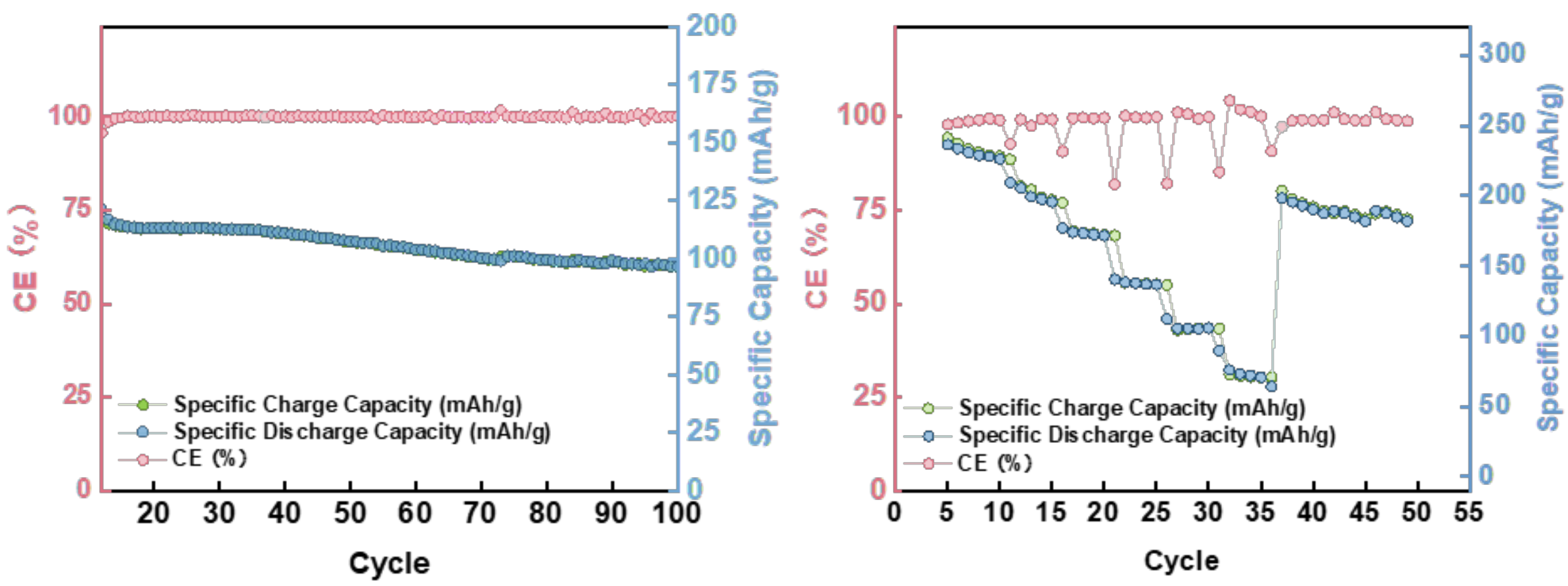

Fig. R2 Full cell performance using vanadium oxide cathode chemistry.

Response to Reviewer #2:

The manuscript addresses a timely and practically significant problem in aqueous zinc ion battery research—the severe capacity loss of metal anodes during calendar aging. The authors propose an unconventional "ultra-dilute" electrolyte strategy (0.1 M ZnSO4 + Li2SO4 + DMF in D2O) as an alternative to the widely adopted high-concentration "water-in-salt" concept, claiming that corrosion is mainly driven by solvated rather than free water molecules. The study combines density-functional analysis, spectroscopy, and electrochemical testing and reports markedly improved capacity retention and cycling stability.

While the topic is relevant and potentially influential, the current work can be improved to meet the standard expected for publication in Nature Communications. The central mechanistic claim—that solvated water dominates corrosion—is not experimentally verified, the stability of the complex electrolyte are insufficiently characterized. The study's conceptual framework is appealing but currently more correlative than causal. Before acceptance, the authors should address the additional validation and mechanistic clarifications outlined below.

<u>Authors' Response:</u> We sincerely thank Reviewer #2 for the constructive evaluation of our manuscript. We take this opportunity to revise our manuscript and address the concerns raised by adding targeted experiments and expanded analyses as detailed below.

1. Mechanistic basis: "solvated water drives corrosion" lacks direct proof

The central hypothesis that solvated rather than free water molecules govern Zn corrosion is the conceptual foundation of the paper, yet it is supported solely by static DFT/COHP calculations and correlative electrochemistry. Actually, no experimental discrimination between solvated and free water species is provided. Without spectroscopic validation (e.g., operando Raman, 17O NMR, isotopic exchange kinetics, or neutron scattering), the claim remains speculative.

The assertion that "free water is not reactive" is overstated. Even if free water has higher HER overpotential, the corrosion process in Zn involves mixed chemical-electrochemical steps (Zn + 2H2O → Zn(OH)2 + H2), in which both solvated and bulk species participate.

The authors should consider complementing the DFT results with characterization (for example, Raman or FTIR) to track coordinated vs. free-water vibrational modes under potential control.

<u>Authors' Response:</u> We thank the reviewer for emphasizing the need for stronger experimental validation of our mechanistic framework, which has been detailed in (i) experimental support from Tafel analysis with corrosion kinetics trend beyond DFT and (ii) expanded spectroscopic FTIR validation below.

We also agree that Zn corrosion in aqueous media proceeds through mixed chemical–electrochemical steps and that both "bulk" (free) and "coordinated" (solvated) water can participate in the overall process. Accordingly, we have revised the manuscript to avoid the overstated phrasing "free water is not reactive," and we now consistently describe our conclusion as **free water being less reactive than solvated water** in driving parasitic proton-donation/HER at the Zn surface.

In addition, regarding measurements under potential control, **we clarify that the main phenomenon we target, calendar aging, occurs under open-circuit conditions without an externally applied bias.** The half-cell voltage during the 24 h aging period remains near ~0 V (Supplementary Fig. 22A), consistent with spontaneous corrosion processes occurring under OCV. Therefore, it is not required to interpret the OCV-driven aging behavior emphasized here.

(i) **Experimental support (Tafel analysis) with corrosion kinetics trend beyond DFT.** Our electrochemical kinetics data provide direct experimental support for the hypothesis that corrosion and HER kinetics correlate more strongly with the population of solvated water. Specifically, the Tafel analysis shows a systematic reduction in parasitic reaction kinetics as the Zn^{2+} concentration is decreased. In the near-corrosion-potential region, the polarization curves shift to lower current densities as $ZnSO_4$ concentration decreases, yielding a smaller fitted corrosion current density and thus slower parasitic reaction kinetics (Fig. 2E). This trend is consistent with the expectation that higher Zn concentration promotes Zn^{2+}–H_2O coordination, which weakens O–H bonds and enhances proton donation, thereby accelerating HER/corrosion. Therefore, we conclude that dilution reduces the population of Zn^{2+}-H_2O and suppresses HER/corrosion, which aligns with prior mechanistic studies showing that Zn^{2+}–H_2O interactions can substantially weaken the O–H bond strength of solvated water, increasing its propensity to act as a proton donor relative to bulk water *[Adv. Mater. 2022, 34, 45, 2206754]*.

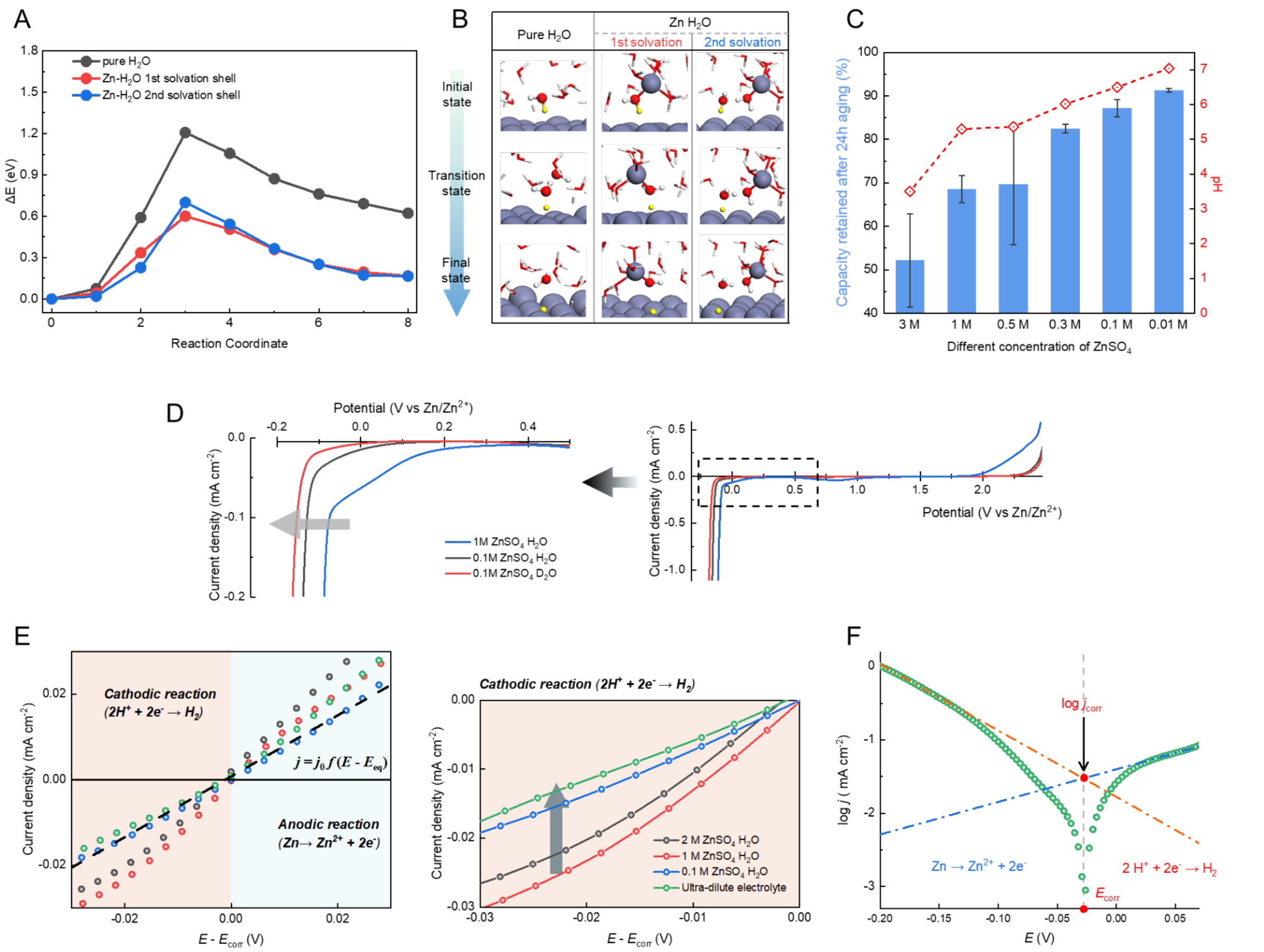

Fig. 2: Reducing salt concentration and substituting D_2O to decrease water reactivity. (A) Reaction profile of proton-donating process at -0.8 V vs SHE with different proton sources and reaction pathways. (B) Atomic structures of initial state, transition state, and final state with different H_2O source. (C) Relationship between capacity retention after 24 hours of aging and various $ZnSO_4$ concentrations using H_2O as solvent, along with the corresponding pH values. (D) Overall electrochemical stability windows of the electrolytes at 0.1 M $ZnSO_4$ with and without D_2O, including magnified views of the cathodic regions. (E) Representative LSV data. The dashed line indicates the fitted response of the ultra-dilute electrolyte at equilibrium potential ($E = E_{eq}$), including magnified views of the cathodic reaction. (F) Tafel plot of ultra-dilute electrolyte derived by LSV measurements.

(ii) Expanded spectroscopic validation (FTIR)

We have expanded the discussion of our FTIR results (Supplementary Fig. 2A, C) to more explicitly link dilution to changes in the water vibrational signatures and to provide a clearer spectroscopic proxy for coordination effects across concentrations.

Supplementary Fig. 2C compares FTIR spectra for multiple $ZnSO_4$ concentrations with pure water as baselines and shows a clear trend that dilution reduces the fraction of solvated water relative to free water estimated from the intensity, consistent with our mechanistic picture.

Manuscript Edits:

Page 9:

Notably, pure H_2O and D_2O show extremely low j_{corr} values and negative E_{corr} (Supplementary Fig. 4A-C), suggesting that free water molecules alone are less aggressive to Zn compared to solvated water.

Page 17:

We emphasize that despite the presence of free water molecules in the bulk electrolyte, excellent cycling performance is retained, suggesting that free water is less reactive than solvated water to Zn anode corrosion.

Page S7

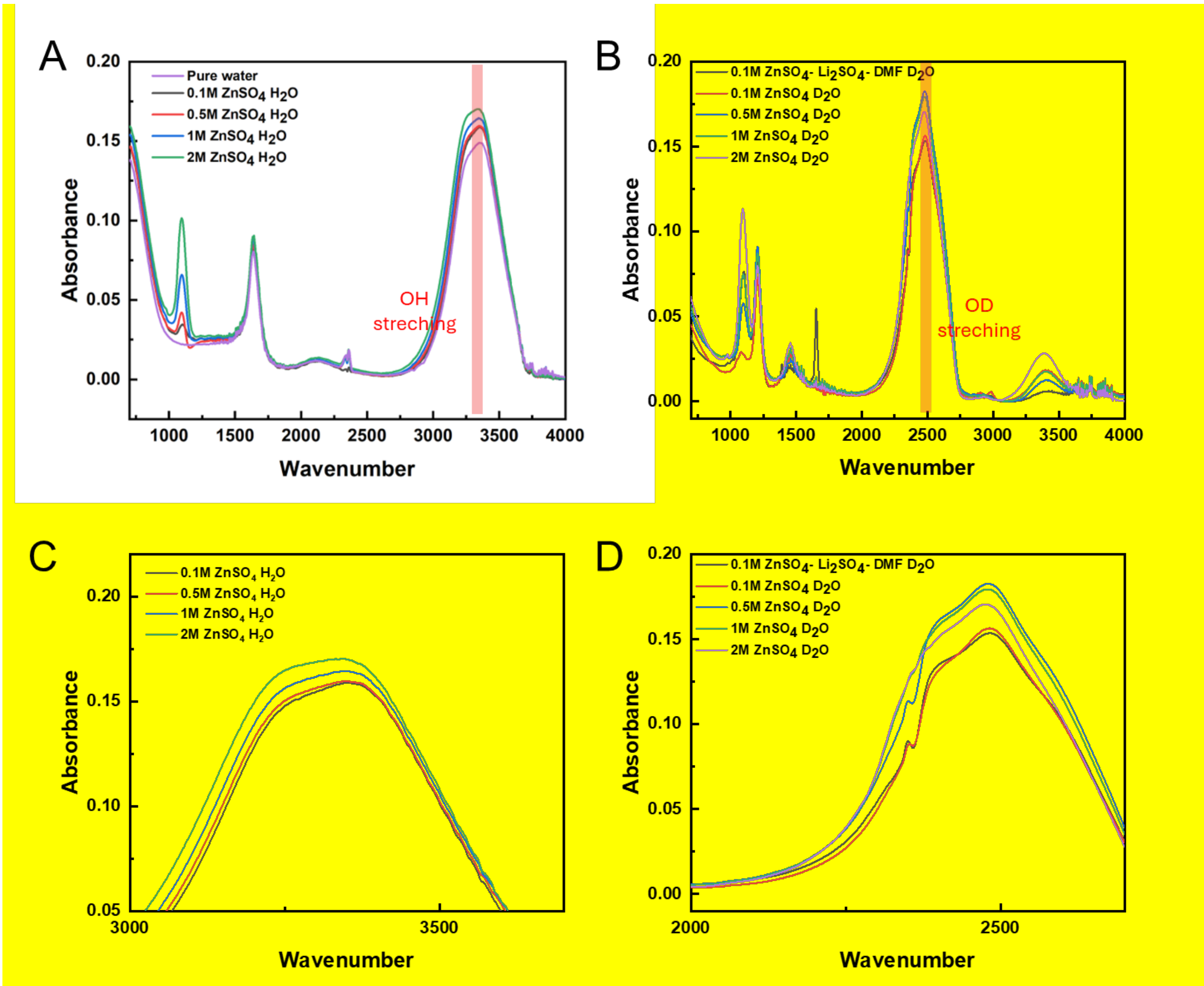

Supplementary Fig. 2. The effect of Zn^{2+} concentration on the water reactivity. Fourier Transform Infrared Spectroscopy (FTIR) spectra between 700 and 4,000 cm^{-1} with different salt concentrations in (A, C) H_2O-based electrolyte and (B, D) D_2O-based electrolyte.[2]

2. Validation of electrolyte composition and stability

The multi-component electrolyte ($ZnSO_4$ + Li_2SO_4 + DMF + D_2O) introduces several reactive species. Stability of each under operating conditions is not demonstrated. DMF is known to decompose reductively on Zn and Cu surfaces, forming amides, formates, and carbonate species. The hypothesized reaction leading to $ZnCO_3$ + ZnO lacks evidence.

Li_2SO_4 can alter ionic transport but may also precipitate as Li_2CO_3 or $Li_2Zn(SO_4)_2{\cdot}xH_2O$ during extended cycling. In the manuscript, no post-cycling XRD or ICP-OES analysis of electrolyte residues is shown.

Therefore, I suggest that the authors consider incorporating additional technologies (such as XPS, ToF-SIMS, or ICP) to support the conclusion.

Authors' Response: In response to the reviewer's request for electrolyte stability validation, we now explicitly include expanded discussion of interphase species supported by (i) post-cycling XPS analysis and (ii) post-cycling XRD. The mechanistic claims have been clarified as follows.

(i) **DMF-enabled interphase formation and decomposition products.**

We agree that DMF can undergo reductive decomposition on Zn metal surfaces and contribute to interphase chemistry. To better support our proposed interphase chemistry,

$$Zn + Zn^{2+} + OH^{-} + HCON(CH_3)_2 + O_2 \rightarrow ZnCO_3 + ZnO + H_2N(CH_3)_2^{+}$$

We added post-cycling XPS analysis on the surface. In addition to ZnO evidence already shown by HRTEM in the manuscript (Fig. 3D-F), XPS reveals a carbonate-associated C 1s component consistent with carbonate species formation at 289.6 eV *[Surf. Interface Anal. 1991, 17, 3, 165-167; J. Spectrosc. 2018, 2018, 1, 2079278.]*, supporting the presence of $ZnCO_3$ products (Supplementary Fig. 14A). We also detect an N-H bonding at 402.0 eV in the N 1s spectrum *[Polymers 2025, 17, 24, 3306; J. Phys. Chem. C 2014, 118, 1, 376-383]*, which is consistent with protonated amine-type species and supports the plausibility of DMF-derived nitrogenous products (i.e., $H_2N(CH_3)_2^{+}$) at the interface (Supplementary Fig. 14B). We revised our manuscript to present the interface analysis more carefully with evidence for carbonate- and amine-containing products.

(ii) **Li_2SO_4 precipitation of Li_2CO_3 / $Li_2Zn(SO_4)_2 \cdot xH_2O$ concerns**

In principle, supporting salts can participate in secondary precipitation. However, sulfate-rich environments are generally reported to hinder Li_2CO_3 precipitation by favoring strong ionic association with SO_4^{2-} and increasing the effective formation barrier for Li_2CO_3 in sulfate-containing media, as reported in previous studies *[Ind. Eng. Chem. Res. 2024, 63, 11, 4918-4933; Cryst. Growth Des. 2019, 19, 12, 6994-7006]*. In addition, we noted that post-cycling XRD does not show detectable Li_2CO_3 or $Li_2Zn(SO_4)_2 \cdot xH_2O$ peaks (Fig. R3), supporting the conclusion that Li_2CO_3 or $Li_2Zn(SO_4)_2 \cdot xH_2O$ precipitation does not happen under our conditions.

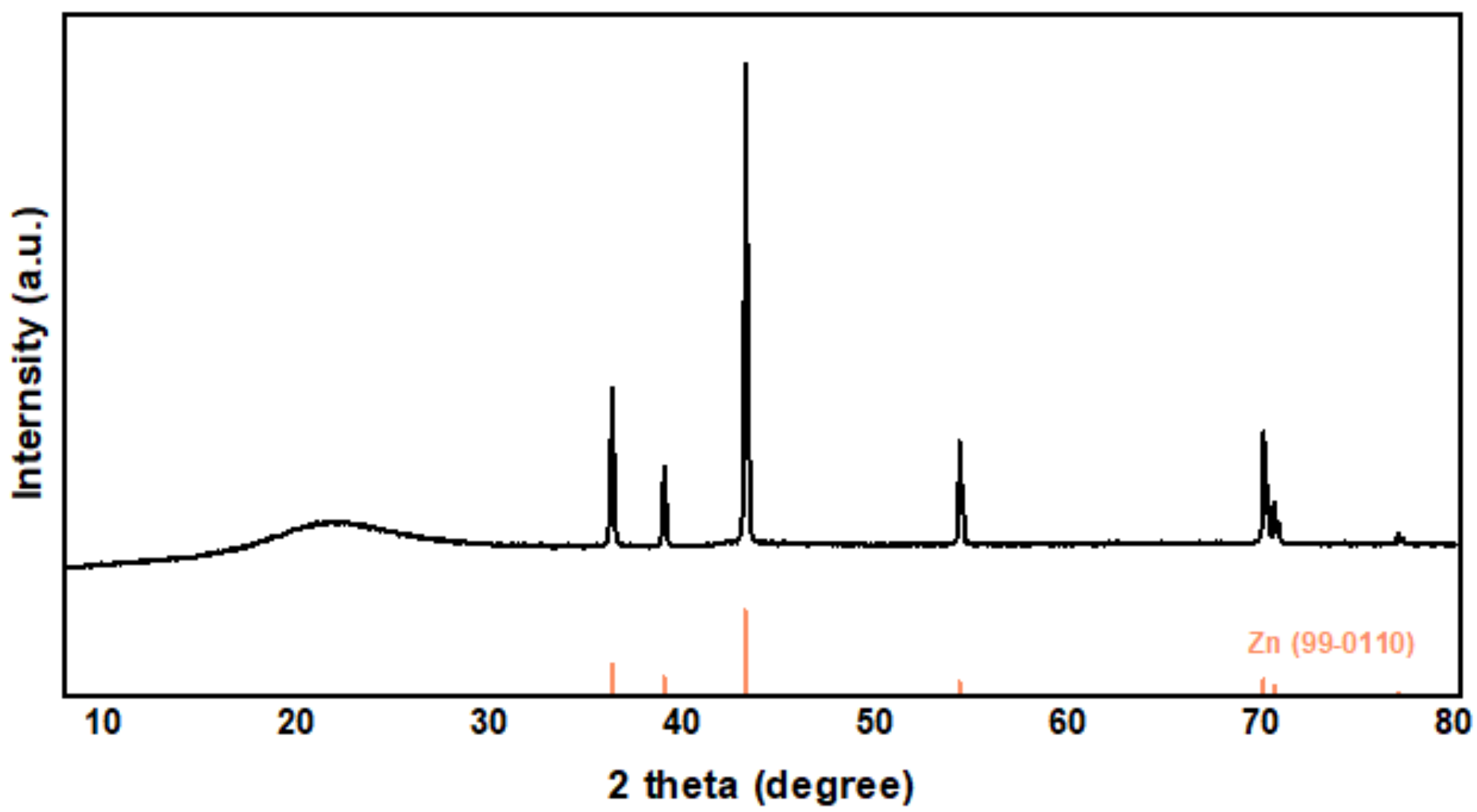

Fig. R3 Post-cycling XRD analysis on the surface.

Manuscript edits:

Page 11-12:

XPS reveals a carbonate-associated C 1s component consistent with carbonate species formation at 289.6 eV,[38, 39] supporting the presence of $ZnCO_3$ products (Supplementary Fig. 14A). We also detect an N-H bonding at 402.0 eV in the N 1s spectrum,[40, 41] which is consistent with protonated amine-type species and supports the plausibility of DMF-derived nitrogenous products (i.e., $H_2N(CH_3)_2^+$) at the interface (Supplementary Fig. 14B).

Page S19:

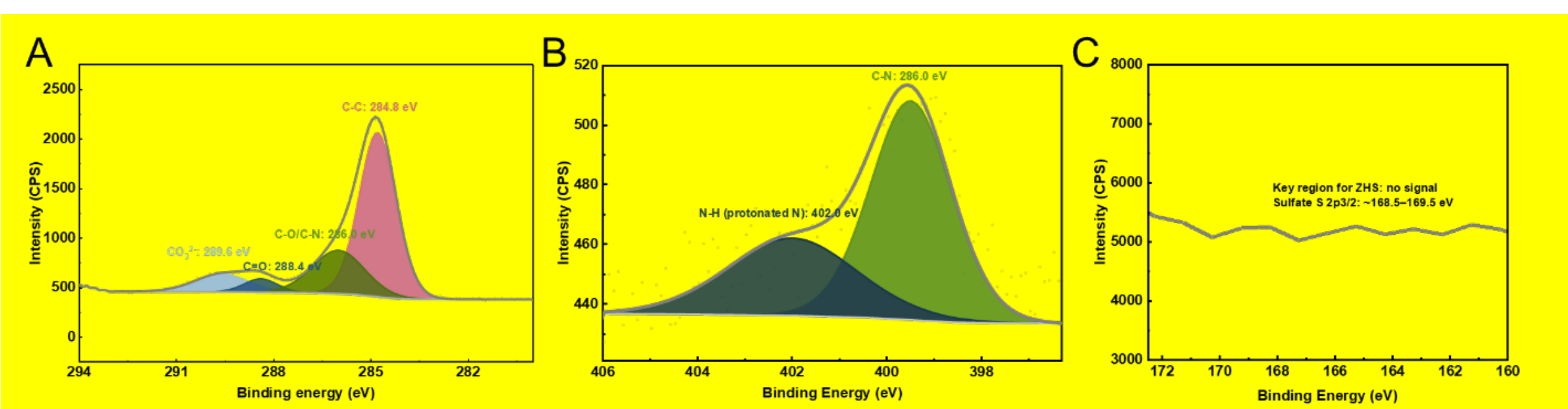

Supplementary Fig. 14. Post-cycling XPS analysis on the surface. (A) C 1s, (B) N 1s, and (C) S 2p.

3. Electrochemical testing details should be provided

First, the aging protocol ("24 h rest after plating") lacks procedural details: OCV conditions, temperature control, and self-discharge should be monitored. Besides, electrolyte volume-to-electrode-area ratio is critical for ultra-dilute systems; it must be reported.

<u>Authors' Response:</u> In the revised manuscript, we provide a zoomed-in voltage–time profile during the 24 h rest period (Supplementary Fig. 22A-B) to show the OCV conditions, confirming the calendar aging was performed under open-circuit conditions without external bias. We also monitored the self-discharge behavior of anode-free full cells (Supplementary Fig. 21B), which exhibited a CE of 86.8% after 2 h of rest. All aging experiments were conducted at 25 °C, and the electrolyte volume-to-electrode-area ratio was reported to be 30 μL cm^{-2} for both half-cell calendar-aging tests and anode-free full-cell tests. This number falls within typical coin-cell electrolyte loadings reported in the literature and provides a practical balance between ensuring adequate wetting and avoiding unrealistically flooded conditions *[Nat. Energy 2022, 7, 2, 186-193; Nat. Commun. 2024, 15, 7771; ACS Energy Letters 8 (9), 3820-3828]*. This additional information has now been explicitly added in the Methods section of our revised manuscript.

Manuscript edits:

Page 21:

All aging experiments were conducted at 25 °C, and the electrolyte volume-to-electrode-area ratio was reported to be 30 μL cm^{-2} for both half-cell calendar-aging tests and anode-free full-cell tests. This number falls within typical coin-cell electrolyte loadings reported in the literature and provides a practical balance between ensuring adequate wetting and avoiding unrealistically flooded conditions.[8, 47, 48]

Page S26:

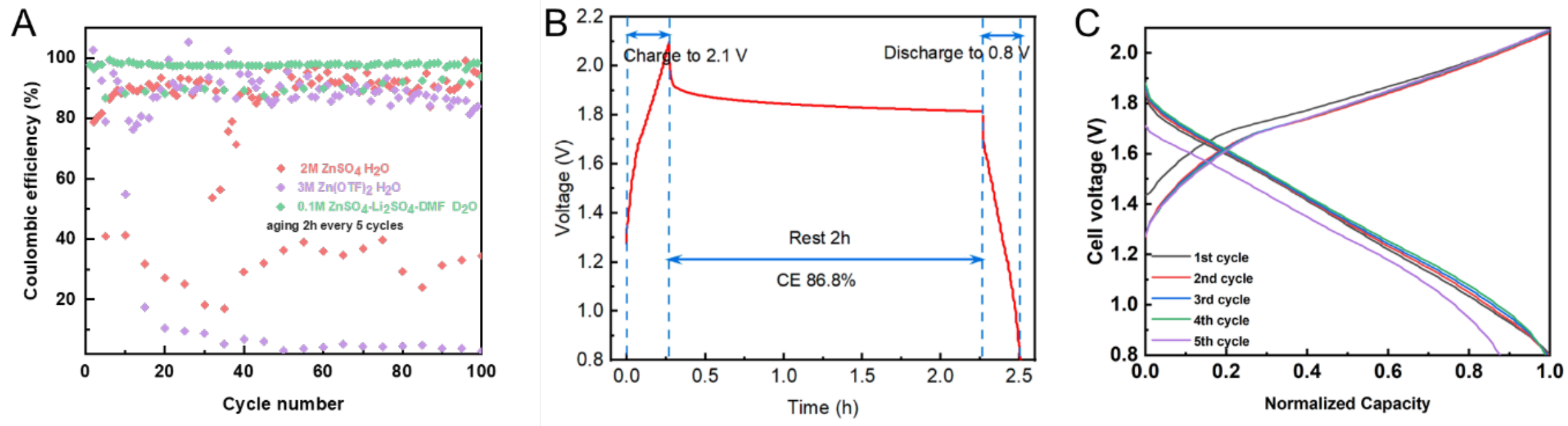

Supplementary Fig. 21. (A) CE of Graphite paper|ZMO anode-free cell in various electrolytes, (B) Voltage vs. time profile, and (C) voltage vs. capacity profile of Graphite paper|ZMO anode-free cell in our optimized electrolyte during calendar aging.

Page S27:

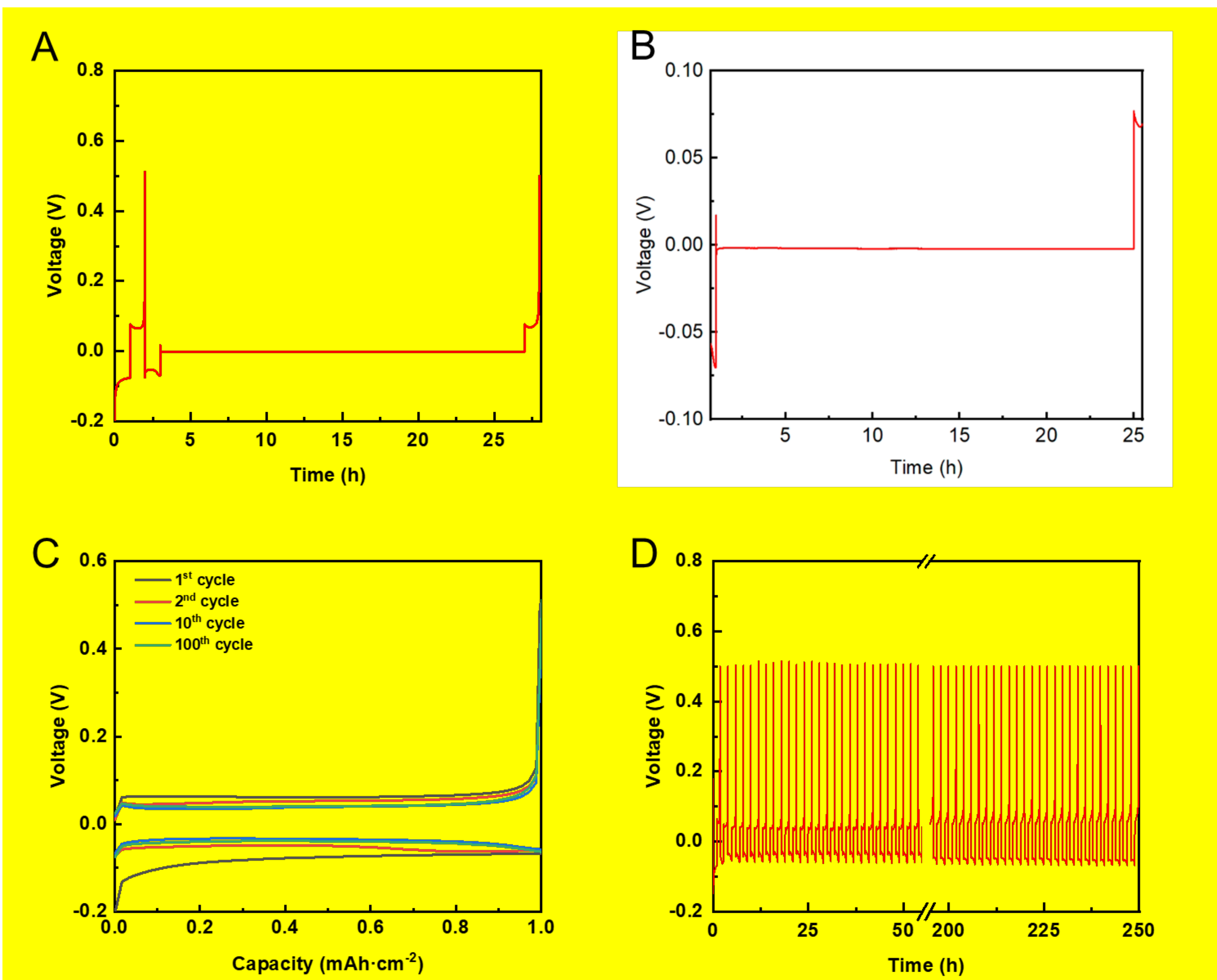

Supplementary Fig. 22. Voltage profiles of 1 mAh cm^{-2} plated Zn metal on graphite paper

substrate. (A-B) Voltage profile with 24 h calendar aging. (B) Voltage profile without calendar aging. (C) Overall voltage profile.

4. Absence of impedance and transport analysis

The claim that Li2SO4 addition "substantially decreases ionic transport resistance" is only qualitatively described. So, the authors should provide full Nyquist plots and fitted equivalent-circuit parameters before and after aging; Zn2+ transference numbers and apparent diffusion coefficients from GITT or EIS to quantify transport effects of dilution and isotopic substitution. In addition, discussion of how 0.1 M Zn2+ maintains dendrite-free deposition without severe concentration polarization—contradictory to classical analysis, should also be included.

Authors' Response: We thank the reviewer for this constructive suggestion. We agree that impedance and transport quantification are important to support our statements, and that concentration polarization must be discussed when interpreting Zn deposition behavior in dilute electrolytes. In the revised manuscript, we therefore added (i) full Nyquist plots with equivalent-circuit fitting before and after 24 h aging, (ii) Bruce–Vincent–type apparent transference number measurements as a consistent comparative transport metric across different formulations, and (iii) an expanded discussion with new SEM evidence reconciling classical concentration-polarization theory with the dendrite-free performance of our *optimized* electrolyte.

(i) Full Nyquist plots with equivalent-circuit fitting before and after 24 h aging.

We performed EIS measurements (Supplementary Fig. 7) for three representative electrolytes before and after 24 h calendar aging: (a) 0.1 M $ZnSO_4$ in H_2O, (b) 0.1 M $ZnSO_4$ + 0.5 M Li_2SO_4 in H_2O, and (c) 0.1 M $ZnSO_4$ + 0.5 M Li_2SO_4 + 5 vol% DMF in D_2O (our optimized electrolyte). Nyquist plots were fitted with a standard equivalent circuit (Supplementary Fig. 7A), consistent with prior Zn battery EIS practice *[Nat. Commun. 2023, 14, 3067]*. From the fitting, we quantified:

- **Electrolyte resistance R_e (high-frequency intercept)** as a transport-related metric (Supplementary Fig. 7G). Adding Li_2SO_4 to 0.1 M $ZnSO_4$ in H_2O reduces R_e from 38.20 Ω to 8.10 Ω (~79% decrease), providing direct quantitative evidence that Li_2SO_4 substantially decreases ionic transport resistance, consistent with our

original qualitative statement. Our optimized electrolyte also shows a lower R_e (15.70 Ω) than 0.1 M $ZnSO_4$ in H_2O (38.20 Ω).

- **Aging-induced charge-transfer resistance growth**, quantified as ΔRct = Rct(24 h) − Rct(0 h) (Supplementary Fig. 7H). The baseline 0.1 M $ZnSO_4$ in H_2O shows a large ΔRct (162.4 Ω), while 0.1 M $ZnSO_4$ + 0.5 M Li_2SO_4 in H_2O remains similarly large (160.2 Ω), indicating that improving bulk transport alone does not sufficiently stabilize the interface during storage. In contrast, our optimized electrolyte shows a much smaller ΔRct (76.0 Ω), consistent with reduced interfacial degradation enabled by DMF-assisted interphase formation and the deuterated solvent environment.

(ii) Bruce–Vincent–type apparent transference number measurements.

We further measured the apparent transference number ($t^{+}{}_{,app}$) in Zn||Zn symmetric cells using the protocol originally developed by Bruce and Vincent and widely used as a comparative transport metric (Supplementary Fig. 8) *[J. Am. Chem. Soc. 2022, 144, 17, 7583-7587; Polymer 1987, 28, 13, 2324-2328]*. Because our electrolytes contain multiple ionic species (Zn^{2+}/Li^{+}/SO_4^{2-} and potentially solvated complexes), extracting a strict single-ion Zn^{2+} transference number would require more specialized treatments. Therefore, we report $t^{+}{}_{,app}$ as a consistent comparative descriptor across different formulations (Supplementary Fig. 8), whose trends support our transport claims:

- **Isotopic substitution alone:** minimal change at fixed $ZnSO_4$ concentration (0.17 in H_2O vs 0.16 in D_2O at 0.1 M $ZnSO_4$), indicating D_2O substitution does not substantially alter the cationic transport fraction.
- **Concentration increase:** decreased $t_{+,app}$ at high concentration (0.13 for 2 M $ZnSO_4$ in D_2O), consistent with stronger ion–ion correlations at higher salt concentrations *[J. Chem. Phys. 2023, 158, 16, 161104; J. Am. Chem. Soc. 2022, 144, 17, 7583-7587]*.
- **Supporting salt and interphase regulation:** our optimized electrolyte shows a substantially higher $t_{+,app}$ = 0.32, approximately doubling the baseline value, indicating that DMF enables an interphase that supports ion transport and stabilizes deposition.

(iii) A discussion with new SEM evidence reconciling classical concentration-polarization theory with the dendrite-free performance of our optimized electrolyte.

We agree that in classical electrodeposition theory, lowering the salt concentration shortens the transition time to interfacial ion depletion, often discussed in terms of Sand's time, thereby increasing the propensity for dendritic growth *[Phys. Rev. A 1990, 42, 12, 7355-7367; J. Electrochem. Soc. 2006, 153, 5, C357-C364]*. To directly address this point, we added new SEM characterizations of Zn deposited at 1 mA cm^{-2} and 1 mAh cm^{-2} across 2 M, 0.5 M, 0.1 M, and 0.01 M $ZnSO_4$ electrolytes. Consistent with classic analyses and prior Zn electrodeposition studies *[Heliyon 2019, 5, 10, e02681; J. Electrochem. Soc. 2006, 153, 5, C357-C364]*, we observe a clear concentration-dependent morphological evolution, where higher concentrations yield orderly, closely packed deposits, whereas lower concentrations lead to increasingly clustered and ultimately mossy and dendritic features, indicative of stronger mass-transport control and interfacial depletion (Supplementary Fig. 9).

Importantly, we now explicitly clarify that low $ZnSO_4$ concentration alone does *not* guarantee dendrite-free Zn deposition. Instead, our central design principle is to reduce Zn^{2+} concentration, thereby minimizing corrosive solvated water (which drives calendar-aging stability), while dendrite suppression during cycling requires a complementary interfacial/morphological-regulation strategy. In our optimized electrolyte, this is achieved by combining:

- Supporting salt (Li_2SO_4) to improve ionic conductivity and mitigate concentration polarization.
- DMF additive to promote in situ formation of a conformal interphase that regulates Zn nucleation/growth toward compact morphologies *[Angew. Chem. Int. Ed. 2025, 64, 16, e202501359; Nat. Commun. 2024, 15, 3914; Joule 2022, 6, 5, 1103-1120; ACS Energy Lett. 2023, 8, 3, 1613-1625]*.
- Isotope substitution (D_2O) to reduce solvent activity or proton-donor reactivity (kinetic isotope effect), suppressing parasitic reactions that otherwise roughen the interface *[Angew. Chem. Int. Ed. 2023, 62, 16, e202300608; Angew. Chem. Int. Ed. 2022, 61, 25, e202203137]*.

Manuscript Edits

Page 10-11:

As shown in Supplementary Fig. 7, adding Li_2SO_4 substantially decreases the overall ionic transport resistance, offsetting the drawbacks of using an ultra-dilute Zn^{2+} without notably altering the reactivity of solvated water in the electrolyte.

Page 22:

The apparent transference number (t^{+}_{app}) was tested in Zn||Zn symmetric cells using the protocol originally developed by Bruce and Vincent and widely used as a comparative transport metric. Briefly, cells were rested for 12 h, then polarized at ΔV = 10 mV for 1 h to reach steady state. Then, EIS was collected before/after polarization to obtain R_0 and R_{ss}, and t^{+}_{app} was calculated following the Bruce-Vincent framework:

$$t_{+}=\frac{I_{ss}(\Delta V-I_0R_0)}{I_0(\Delta V-I_{ss}R_{ss})}$$

Page S12:

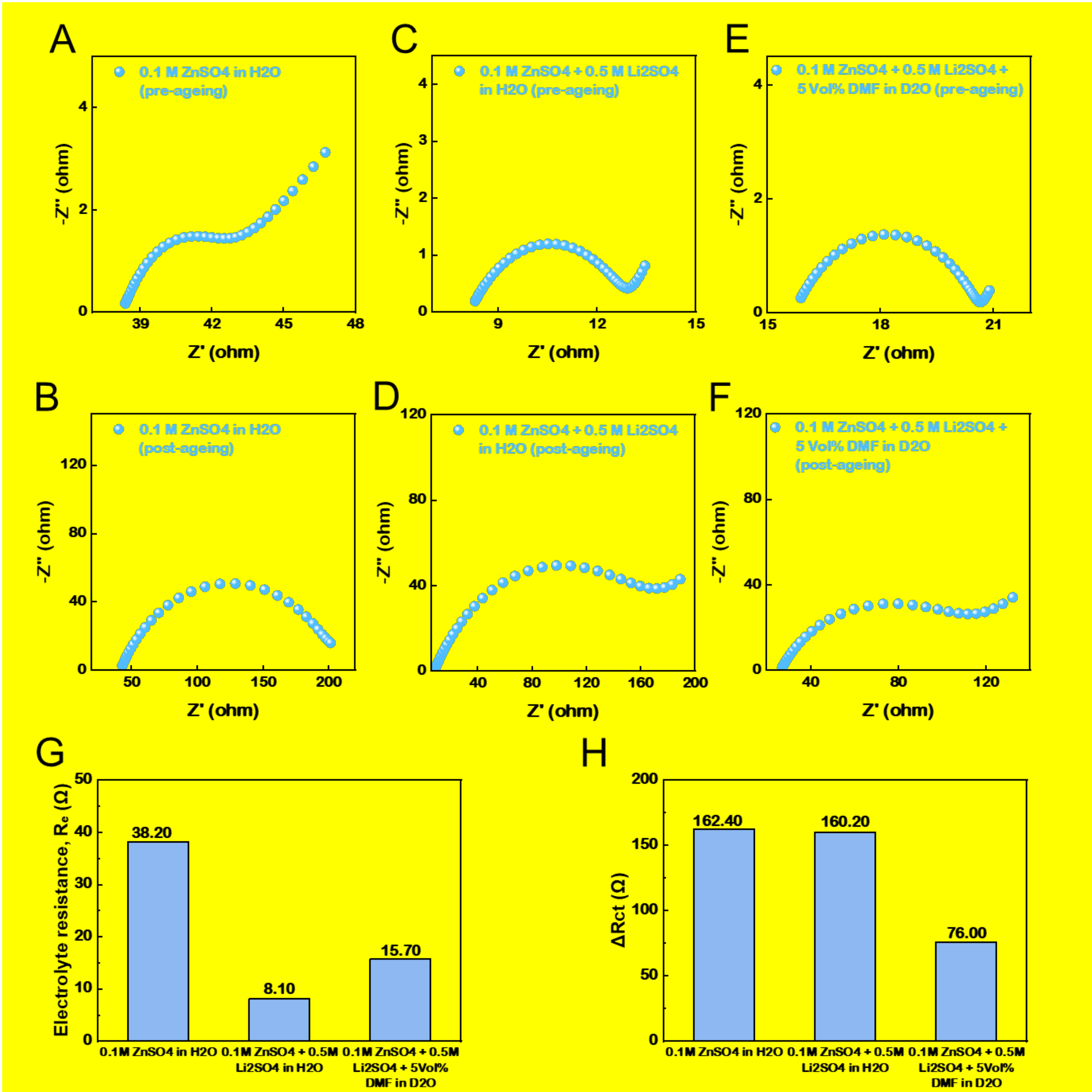

Supplementary Fig. 7. Cation concentration effects on electrolyte ionic conductivity. EIS profile before and after aging in (A-B) 0.1 M $ZnSO_4$ in H2O, (C-D) 0.1 M $ZnSO_4$ + 0.5 M Li_2SO_4 in H_2O, and (E-F) 0.1 M $ZnSO_4$ + 0.5 M Li_2SO_4 + 5 vol.% DMF in D_2O with equivalent circuit for fitting inset in (A). Summarized (G) electrolyte resistance and (H) aging induced charge transfer resistance growth.

Page S13:

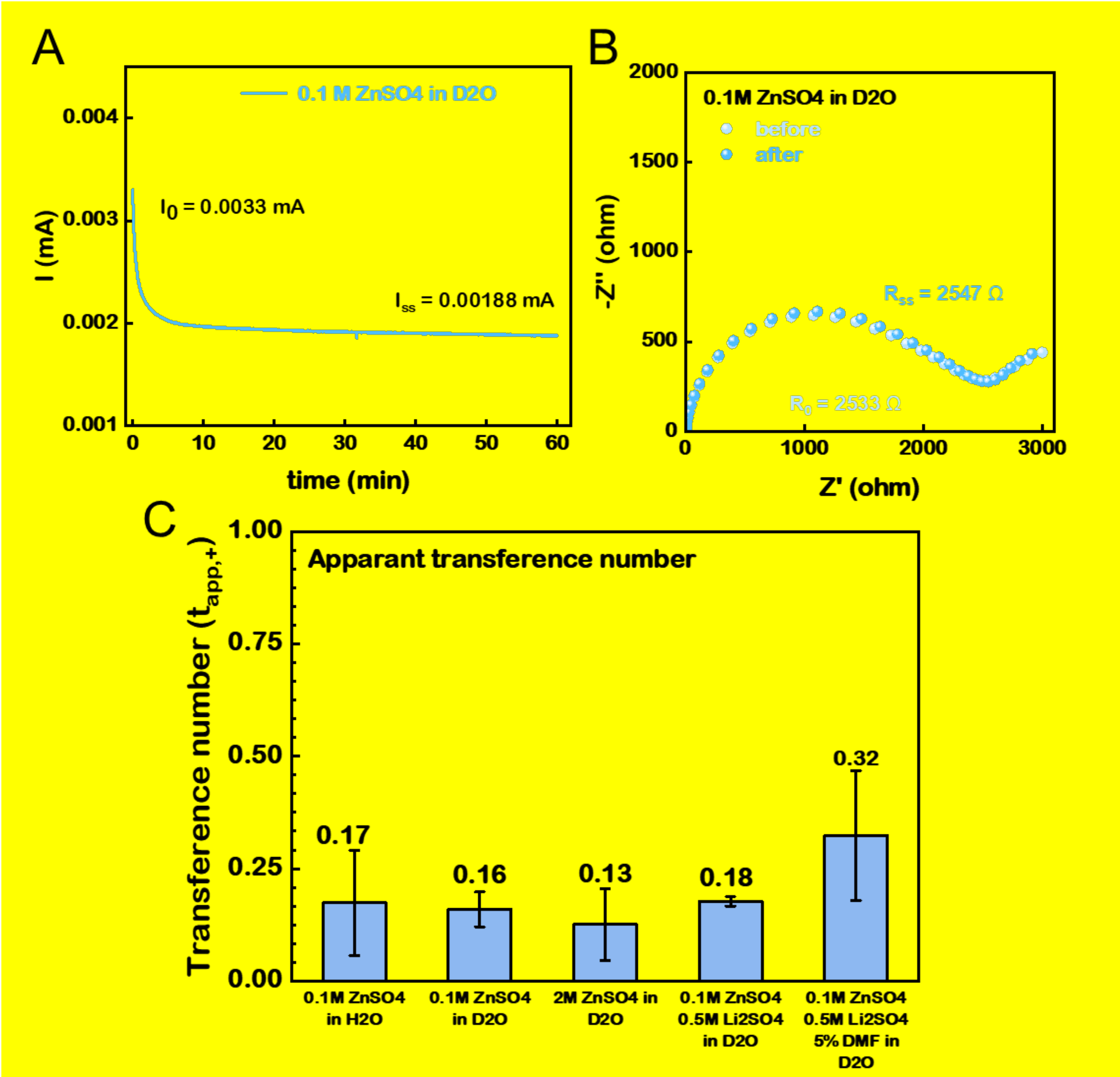

Supplementary Fig. 8. (A-B) Bruce Vincent method and (C) apparent transference number.

Page S14:

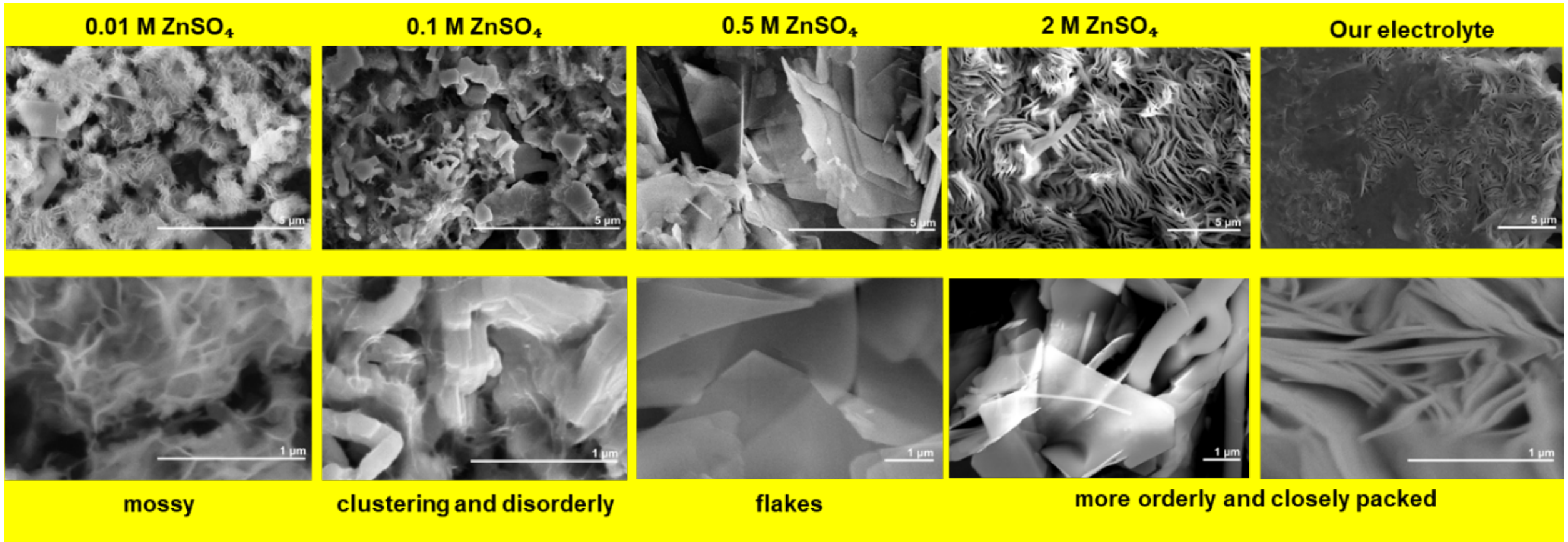

Supplementary Fig. 9. Zn deposition morphology at 1 mA cm^{-2} and 1 mAh cm^{-2} across different electrolytes.

In classical electrodeposition theory, lowering the salt concentration shortens the transition time to interfacial ion depletion, often discussed in terms of Sand's time, thereby increasing the propensity for dendritic growth.[3, 4] Consistent with classic analyses and prior Zn electrodeposition studies,[3, 5] we also observe a clear concentration-dependent morphological evolution, where higher concentrations yield orderly and closely packed deposits, whereas lower concentrations lead to increasingly clustered, and ultimately mossy and dendritic features, indicative of stronger mass-transport control and interfacial depletion (Supplementary Fig. 9). We clarify that low $ZnSO_4$ concentration alone does not guarantee dendrite-free Zn deposition. Instead, our central design principle is to reduce Zn^{2+} concentration, thereby minimizing corrosive solvated water (which drives calendar-aging stability), while dendrite suppression during cycling requires a complementary interfacial/morphological-regulation strategy.

5. Terminology and claims

"Ultra-dilute" is an overstatement for 0.1 M ZnSO4; typical aqueous Zn batteries operate in the 0.5–3 M range, so "low-concentration regime" would be more accurate.

Besides, the concluding statement that the design is "broadly applicable to Fe metal and Na-ion batteries" is speculative and should be deleted unless supporting data exist.

Authors' Response: We thank the reviewer for the wording suggestions. We agree that in conventional aqueous Zn batteries, Zn-salt concentrations are typically in the ~0.5–3 M range. Therefore, we retain the term "ultra-dilute" but now explicitly clarify it as an electrolyte regime

that is about one order of magnitude lower in Zn-salt concentration than conventional practice (here, 0.1 M $ZnSO_4$) at the first mention of the term in the revised manuscript. In addition, we have removed the statement that "The concepts could be extended to other aqueous systems such as Fe metal and Na-ion batteries" to restrict our claims to the Zn systems studied here. However, we acknowledge the importance of the broad applicability of the "solvated-water reactivity" principle. Our group keeps pushing forward, expanding the solvation-corrosion relationships in other aqueous chemistries, and looks forward to seeing progress on this key direction in this field in the near future.

Manuscript edits:

Page 4:

Here, we present an ultra-dilute $ZnSO_4$-based electrolyte (defined here as ~10× lower Zn-salt concentration than conventional aqueous Zn electrolytes (0.5–3 M)) engineered to address the challenge of maintaining calendar aging stability without compromising cycling stability.

6. Besides, there are also some small problems:

(1)For example, The authors should clarify transport-limiting regime: Since ion conductivity is a critical trade-off in ultra-dilute systems, a brief discussion or table comparing the measured ionic conductivities of various electrolyte formulations (possibly from Supplementary Figure 3) would help contextualize the practical feasibility without new experiments.

(2)Add reference to broader aqueous systems: As mentioned above, there is no experimental evidence about the Na- or Fe-based batteries in the manuscript. If the authors want to prove the conceptual generality, they should consider mentioning analogous solvation-corrosion relationships in Na- or Fe-based aqueous chemistries in the Discussion (combined experimental results would be better). This would underscore the broad applicability of the 'solvated-water reactivity' principle.

(3)Optional visualization suggestion: A schematic summarizing how concentration, isotopic substitution, and additive choice synergistically suppress solvated-water activity could be added to the main text or Supplementary Information for readers' clarity. No additional experiments are needed.

<u>Authors' Response</u>: (1) To quantitatively contextualize feasibility and clarify the transport trade-off in the low-concentration regime, we have now measured the ionic conductivity for every electrolyte formulation (see Methods) tested in Supplementary Fig. 3A using 0.1 M $ZnSO_4$ with H_2O/co-solvent mixtures (v/v = 8:2) and summarized the results in Supplementary Fig. 3B. The results show that co-solvents decrease ionic conductivity by increasing viscosity and/or altering ion association, potentially pushing the system into a transport-limited regime.

(2) We thank the reviewer for the suggestions and have removed the statement that "The concepts could be extended to other aqueous systems such as Fe metal and Na-ion batteries" to restrict our claims to the Zn systems studied here. However, we acknowledge the importance of the broad applicability of the "solvated-water reactivity" principle. Our group keeps pushing forward, expanding the solvation-corrosion relationships in other aqueous chemistries, and looks forward to seeing progress on this key direction in this field in the near future.

(3) For clarity, a schematic (Supplementary Fig. 15) has been provided to summarize how concentration, isotopic substitution, and additive choice synergistically suppress solvated-water activity in the revised Supplementary Information.

Manuscript edits:

Page 22-23:

Ionic conductivity (σ) was measured using a symmetric stainless-steel blocking-electrode configuration (SS|electrolyte|SS) assembled in CR2032 coin cells. A donut-shaped Celgard 2325 separator, saturated with electrolyte, was sandwiched between two stainless steel electrodes. The bulk ionic resistance (R_b) was extracted from the high-frequency intercept of the Nyquist plot obtained via EIS. Ionic conductivity was calculated as

$$\sigma = \frac{L}{R_b \times A}$$

Where L is the compressed separator thickness and A is the cross-sectional area defined by the separator geometry. While the absolute conductivity values are subject to uncertainty arising from this geometric approximation, all measurements were performed under identical conditions, ensuring internally consistent comparisons across the electrolyte formulations.

Page S8:

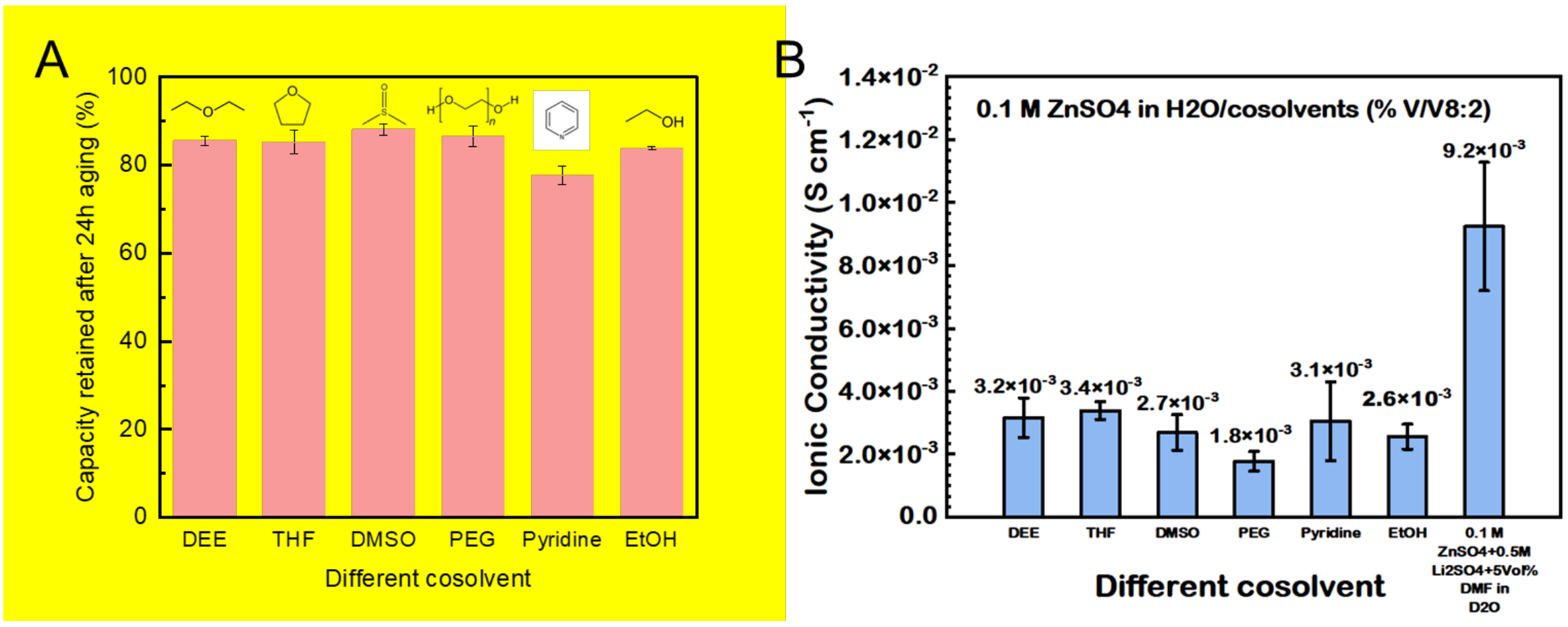

Supplementary Fig. 3. (A) Calendar aging performance and (B) ionic conductivity of different cosolvent electrolytes: 0.1 M $ZnSO_4$ in H_2O/cosolvent mixture (with volume ratio 8:2). Various organice cosolvents include Diethyle ether (DEE), Tetrahydrofuran (THF), Dimethyl sulfoxide (DMSO), Polyethylene glycol (PEG 400), Pyridine, Ethanol (EtOH) (with a Zn deposition capacity of 1 mAh·cm^{-2} at 25 °C under 1mA·cm^{-2} current density, using graphite paper as substrate).

To quantitatively contextualize feasibility and clarify the transport trade-off in the low-concentration regime, we measured the ionic conductivity of each 0.1 M $ZnSO_4$ electrolyte used in the co-solvent screening (H_2O/co-solvent = 8:2, v/v) and summarized the results in Supplementary Fig. 3B. Ionic conductivities show that co-solvents decrease the ionic conductivities, indicating a shift toward transport-limited behavior.

Page S20:

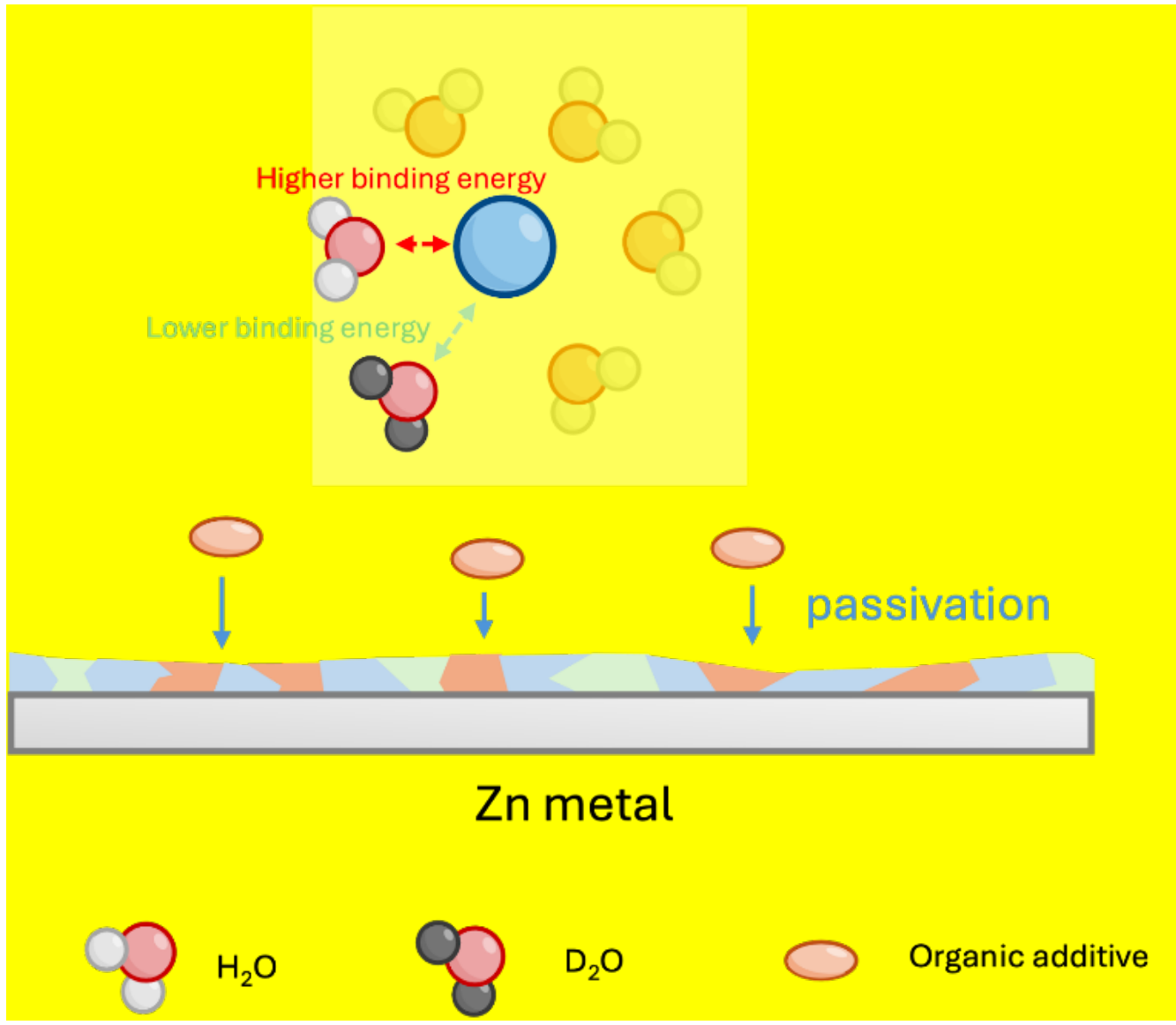

Supplementary Fig. 15. A schematic summarizing how concentration, isotopic substitution, and additive choice synergistically suppress solvated-water activity.

Response to Reviewer #3:

- Title: Ultra-dilute electrolytes for suppressing metal anode corrosion during calendar aging and cycling

- Overall comments: The study addresses calendar aging in Zn-anode batteries. The authors attribute Zn corrosion during rest to solvated water. They introduce an ultra-dilute electrolyte (0.1 M ZnSO4), with optional isotopic solvent substitution and targeted additives. They report >10× lower calendar fade (<1.5% capacity loss after 24 h) while maintaining cycling durability (>3,300 cycles) and high average Coulombic efficiency (~99.8%). If validated, the work suggests electrolyte concentration reduction as a general, mechanistically grounded route to improve both shelf-life and cycling stability in metal-anode chemistries. However, major revisions are required to substantiate the mechanistic claims according to the following detailed comments:

<u>Authors' Response</u>: We appreciate the reviewer's acknowledgement of the strong electrochemical outcomes. Below, we address each of the reviewer's detailed comments point by point.

1. More detailed analysis is required for the Tafel plots in Figure 2F. Mechanism (rds), transfer coefficient, etc.

<u>Authors' Response:</u> We have expanded the Tafel plots analysis to extract separate anodic/cathodic Tafel slopes (α) and quantify transfer coefficients following the standard electrochemical kinetics *[Bard & Faulkner 2001, Electrochemical Methods (2nd ed.), Wiley; Corros. Sci. 2000, 42, 7, 1235-1248; ACS Cent. Sci. 2024, 10, 3, 658–665]* in the revised manuscript. By fitting the linear Tafel regions of the anodic ($Zn \rightarrow Zn^{2+} + 2e^-$) and cathodic branches (HER: $2H^+ + 2e^- \rightarrow H_2$), we can get the Tafel slopes of 227 mV dec^{-1} and 111 mV dec^{-1}, respectively. Using the Tafel form derived from Butler–Volmer kinetics,

$$\alpha = \frac{2.303RT}{nFb}$$

Where R is gas constant (8.314 J mol^{-1}), T=298 K at 25 °C, n is number of electrons involved in the reaction step associated with the respective Tafel branch, F is the Faraday constant (F=96485 C mol^{-1}), and b is Tafel plot derived by fitting the linear Tafel regions of the anodic and cathodic

branches. We estimate the corresponding transfer coefficients (at 298 K) of the anodic and cathodic reactions to be 0.13 and 0.53, respectively. All fitted slopes and the calculated transfer coefficients are now summarized in Supplementary Table 2. Mechanistically, the large anodic slope together with a small transfer coefficient indicates that Zn dissolution in our corrosion-relevant regime is kinetically hindered, representative of an electron-transfer–limited anodic process. On the cathodic side, the ~111 mV dec^{-1} slope and transfer coefficient are close to the canonical ~120 mV dec^{-1} behavior commonly associated with a Volmer-limited HER pathway, where rate limitation is the initial proton–water activation step under near-neutral aqueous conditions. These quantitative descriptors support the conclusion that HER/corrosion kinetics are suppressed in the optimized electrolyte.

Manuscript edits:

Page 8:

To further evaluate the corrosion behavior, we performed Tafel analysis (Fig. 2E-F, Supplementary Table 2).

Page S33:

Supplementary Table 2. All fitted slopes and the calculated transfer coefficients from Tafel plots.

	Anodic reaction ($Zn \rightarrow Zn^{2+} + 2e^-$)	Cathodic reaction ($2H^+ + 2e^- \rightarrow H_2$)
Tafel slope (mV dec^{-1})	227	111
Transfer coefficient	0.13	0.53

The transfer coefficients (α) were calculated using the Tafel form derived from Butler–Volmer kinetics as follows:

$$\alpha = \frac{2.303RT}{nFb}$$

Where R is gas constant (8.314 J mol^{-1}), T=298 K at 25 °C, n is number of electrons involved in the reaction step associated with the respective Tafel branch, F is the Faraday constant (F=96485 C mol^{-1}), and b is Tafel plot derived by fitting the linear Tafel regions of the anodic and cathodic branches.

2. Quantitative analysis of the ionic conductivity is required for the developed electrolytes in comparison with other conventional ones.

Authors' Response: We measured the ionic conductivity of our optimized electrolyte (0.1 M $ZnSO_4$ + 0.5 M Li_2SO_4 + 5 vol% DMF in D_2O) and two representative conventional aqueous Zn electrolytes (2 M $ZnSO_4$ in H_2O and 3 M $Zn(OTf)_2$) under identical conditions (see Methods) as shown in Fig. R4. Our optimized electrolyte exhibits an ionic conductivity of 9.2×10^{-3} S cm^{-1}, which is comparable to 2 M $ZnSO_4$ in H_2O (8.9×10^{-3} S cm^{-1}) and slightly higher than 3 M $Zn(OTf)_2$ in H_2O (6.9×10^{-3} S cm^{-1}). Overall, this quantitative benchmark indicates that, despite the extremely dilute $ZnSO_4$ concentration, our optimized electrolyte maintains practical bulk ionic transport, supporting its feasibility for Zn battery operation.

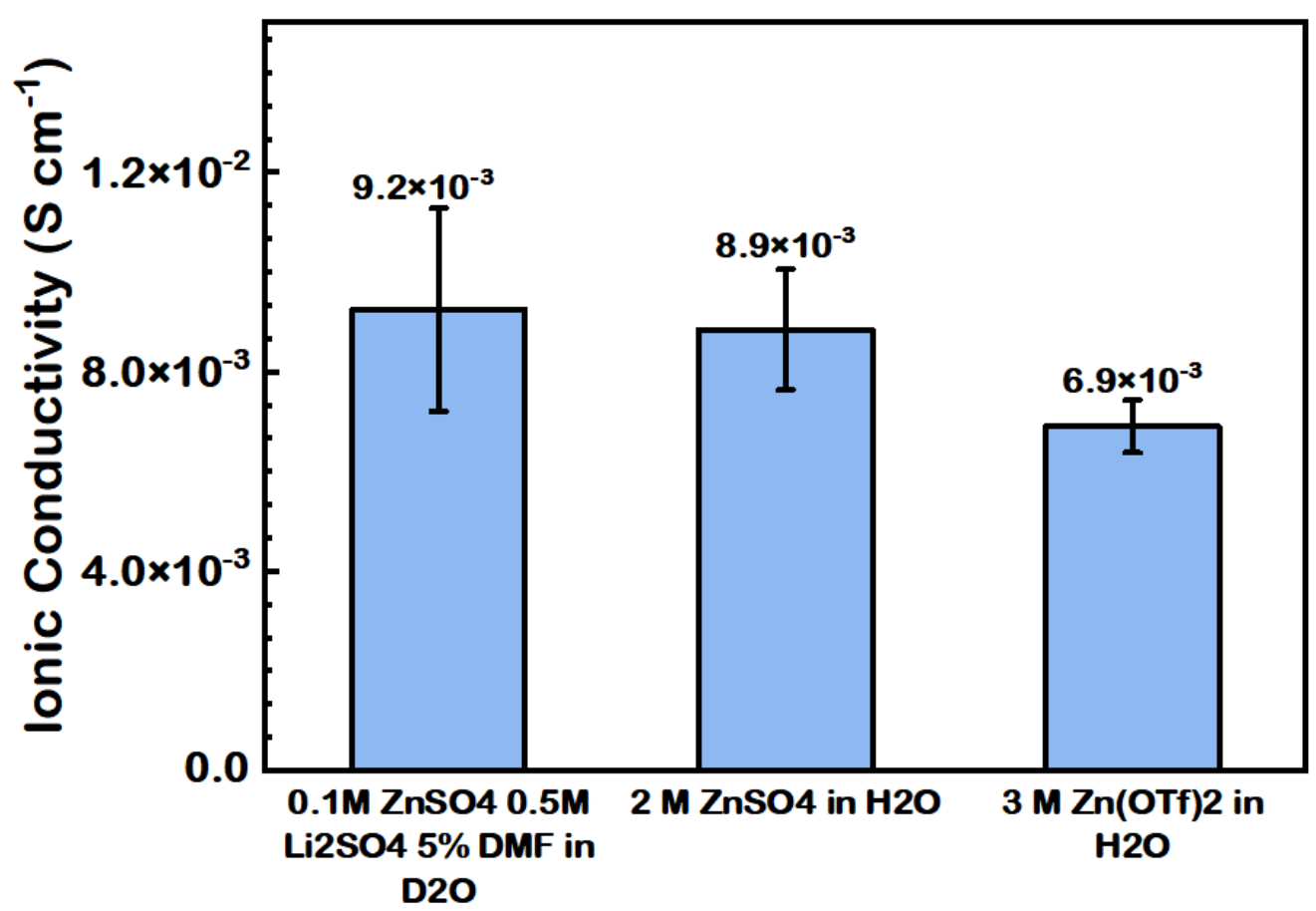

Fig. R4 Ionic conductivity of the ultra-dilute electrolyte and common electrolytes.

Manuscript edits:

Page 22-23

Ionic conductivity (σ) was measured using a symmetric stainless-steel blocking-electrode configuration (SS|electrolyte|SS) assembled in CR2032 coin cells. A donut-shaped Celgard 2325 separator, saturated with electrolyte, was sandwiched between two stainless steel electrodes. The bulk ionic resistance (R_b) was extracted from the high-frequency intercept of the Nyquist plot obtained via EIS. Ionic conductivity was calculated as

$$\sigma = \frac{L}{R_b \times A}$$

Where L is the compressed separator thickness and A is the cross-sectional area defined by the separator geometry. While the absolute conductivity values are subject to uncertainty arising from this geometric approximation, all measurements were performed under identical conditions, ensuring internally consistent comparisons across the electrolyte formulations.

3. Regarding Figure 2D, more thorough 3-electrode cell analysis must be done for the cyclic voltammetry
of reversible zinc deposition and dissolution, especially with respect to the Coulombic efficiency and overpotential.

Authors' Response: We thank the reviewer for requesting a more rigorous three-electrode quantification of Zn deposition/dissolution reversibility. We therefore performed 3-electrode CV coupled with chronocoulometry using Ti as the working electrode and Zn foil as both counter and reference electrodes *[Nat. Mater. 2018, 17, 6, 543-549]* (Fig. R5). This configuration isolates Zn plating/stripping kinetics from counter-electrode polarization and enables a direct assessment of Coulombic efficiency (CE) and overpotential. Integrating the plating and stripping charges yields a CE of 90.2%, indicating that parasitic reactions (primarily HER) contribute a limited fraction (~9.8%) of the charge within the selected voltage window. In addition, defining the onset potential as the point where |j| first exceeds 0.1 mA cm^{-2} *[Curr. Opin. Electrochem. 2023, 37, 101176]*, the Zn plating and stripping onset overpotentials are 0.136 V and 0.011 V, respectively. These results show that, despite operating in a dilute Zn-salt regime, the electrolyte still supports largely reversible Zn deposition/dissolution with modest kinetic polarization, and that the improved calendar-aging behavior reported in the manuscript is not achieved by severely hindering Zn electrochemistry, but by suppressing competing parasitic reactions during rest.

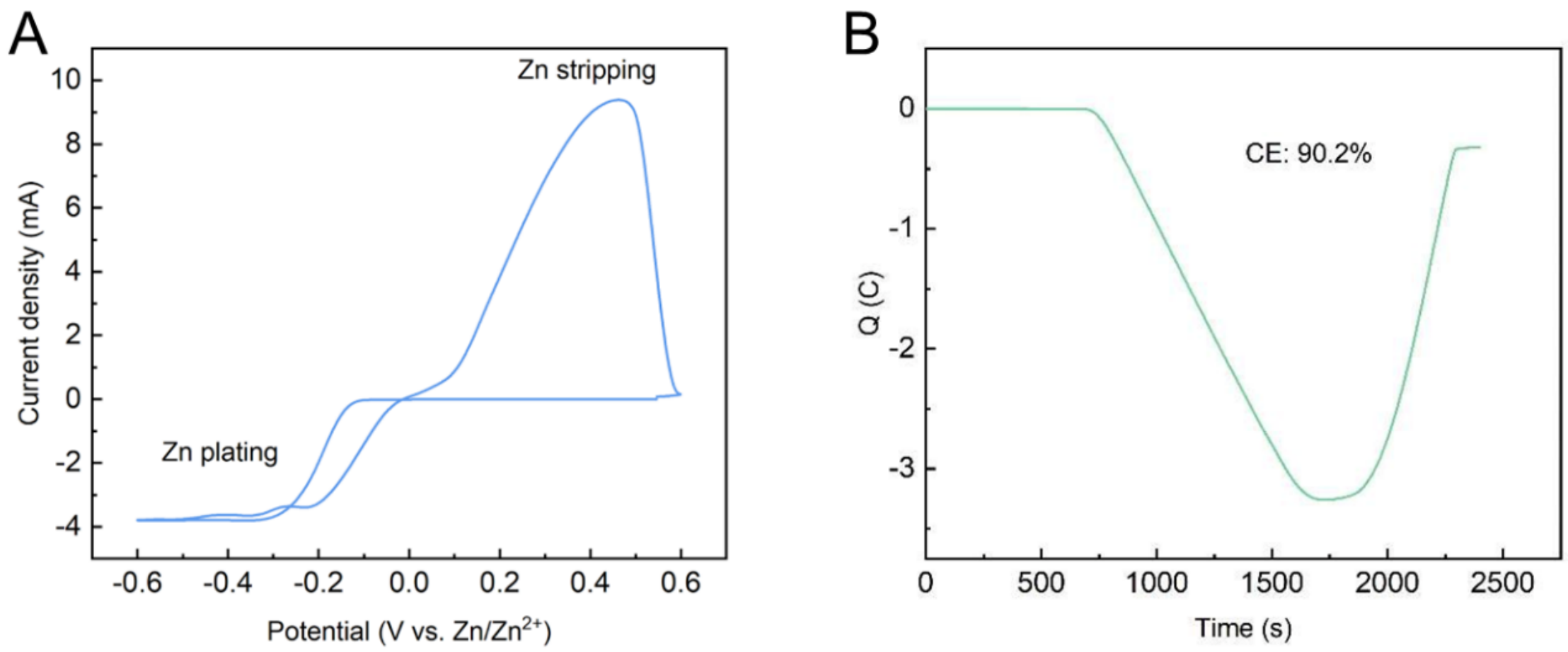

Fig. R5 (A) Cyclic voltammogram of Zn plating/stripping in a three-electrode cell using a Ti disc as the working and Zn as the reference and counter electrodes at a scan rate of 1 mV/s. (B) Chronocoulometry curves.

4. While the concentration of Zn is reduced, the pH is raised. Both factors seems to contribute to the H2O stability in the opposite ways. Quantitative comparison is necessary to determine the effects of O-H bond instability and the raise of HER onset potential by the lower Zn concentration.

Authors' Response: We thank the reviewer for raising this point and would like to clarify that the two trends observed in our electrolyte series, decreasing Zn^{2+} concentration and increasing pH toward neutrality, both act to *increase* the H_2O stability against H_2 evolution, rather than acting in opposite directions. Specifically, upon decreased concentrations, the measured pH intrinsically increases and approaches to ~7 without introducing additional alkalinity, which reflects suppressed Zn^{2+} -induced hydrolysis and a return toward bulk-water behavior with better stability against HER. Our Crystal Orbital Hamilton Population (COHP) analysis (Supplementary Fig. 1C-D) provides a quantitative comparison of O–H bond stability under different pH and Zn concentration environments, showing a weaker O–H bond (2.30 eV) in solvated water that is more susceptible to cleavage and stronger proton-donating ability compared to free water (3.94 eV). In addition, in our $ZnSO_4$ dilution series (Fig. 2C), the pH increases from ~3.4 (3 M $ZnSO_4$) to ~6.5 (0.1 M $ZnSO_4$), corresponding to a ~0.18 V negative shift in the HER equilibrium potential based on the Nernst equation ($\Delta E = -0.059 \times \Delta pH = -0.18$ V) *[J. Phys. Chem. C 2022, 126, 28, 11439-11446]*,

showing a quantitative basis for the raise of HER onset potential (negative shift) and reduced corrosion current in the dilute regime (Fig. 2F).

5. It has been suggested that free water activity is an important factor for the HER and OER, as evidenced by the widening of the voltage stability window in the water-in-salt electrolytes. In this work, theoretical DFT calculation suggested weakened O-H bond by the Zn-coordination. How to reconcile the two contridictory factors? Quantification of the instabilization is necessary to determine which is more important for diminishing HER.

Authors' Response: We thank the reviewer for raising the point and would like to take this opportunity to reconcile the two effects of the Water-in-salt electrolytes regime and our ultra-dilute strategy by explicitly distinguishing (i) the total population of reactive water molecules from (ii) intrinsic reactivity per water molecule.

(i) Water-in-salt electrolytes regime

In water-in-salt electrolytes, the broadened electrochemical stability window is widely attributed to *population-level suppression* of reactive water. They drastically reduce the *total number* of water molecules available and thus the *net* water reactivity, leading to the apparent voltage stability window. *[Science 2015, 350, 938-943; J. Am. Chem. Soc. 2025, 147, 39, 35953-35961]*

(ii) Our ultra-dilute strategy

Our DFT results do not contradict the water-in-salt electrolyte framework but isolate a *molecular-scale factor* that quantitatively reports that Zn^{2+} coordination weakens the O–H bonding energy of coordinated water (solvated water) relative to uncoordinated water (free water), thereby increasing its propensity to participate in HER. This trend is consistent with prior mechanistic studies showing that strong cation–water interactions can increase water reactivity by perturbing the O–H bond and facilitating hydrogen evolution *[Energy Storage Mater. 2023, 62, 102941; Battery Energy 2024, 3, 2, 20230063]*. Similarly, our goal is to minimize the absolute concentration of the more reactive species (solvated water), because our electrochemical trends predict and support the conclusion that this subset is more reactive than free water.

To avoid misunderstanding, we revise the wording to "weakened O–H bonding energy upon Zn^{2+} coordination relative to free water" in the manuscript.

Manuscripts edits:

Page 3-4:

A notable example is the "water-in-salt" electrolyte, which uses 1 m $Zn(TFSI)_2$ and 20 m LiTFSI or 30 m $ZnCl_2$ to reduce water activity by promoting the coordination of Zn^{2+} with TFSI anions.[14, 15] This strategy drastically reduces the total number of water molecules available and thus the net water reactivity, leading to the apparent voltage stability window.

Page 6:

Overall, Zn coordination in solvated water significantly weakens the O–H bond and enhances the HER by stabilizing the transition and final states compared to free water (Figure 2B).

6. Voltage profiles are to be represented in the main figures for the full cell operation of Fig. 4G and 5E.

Authors' Response: We have included the voltage profiles for the full-cell operation shown in Fig. 4G and 5E and incorporated them into the revised Supplementary Information for better figure arrangement (Supplementary Fig. 21B-C, Supplementary Fig. 23).

Manuscript edits:

Page 18:

Under 4 C condition, this cell achieves excellent cycling stability, maintaining 80% of its capacity over 3,300 cycles with an average CE of 99.8% (Figure 5E and Supplementary Fig. 23), which is comparable with previous reports.[14, 45, 46]

Page S26:

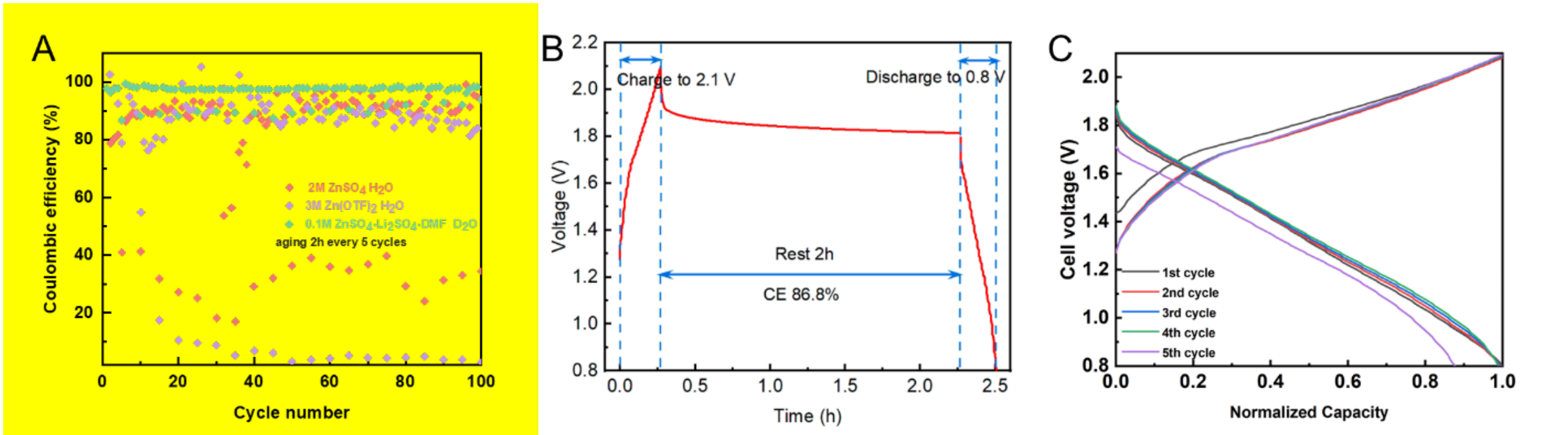

Supplementary Fig. 21. (A) CE of Graphite paper|ZMO anode-free cell in various electrolytes, (B) Voltage vs. time profile, and (C) voltage vs. capacity profile of Graphite paper|ZMO anode-free cell in our optimized electrolyte during calendar aging.

Page S28:

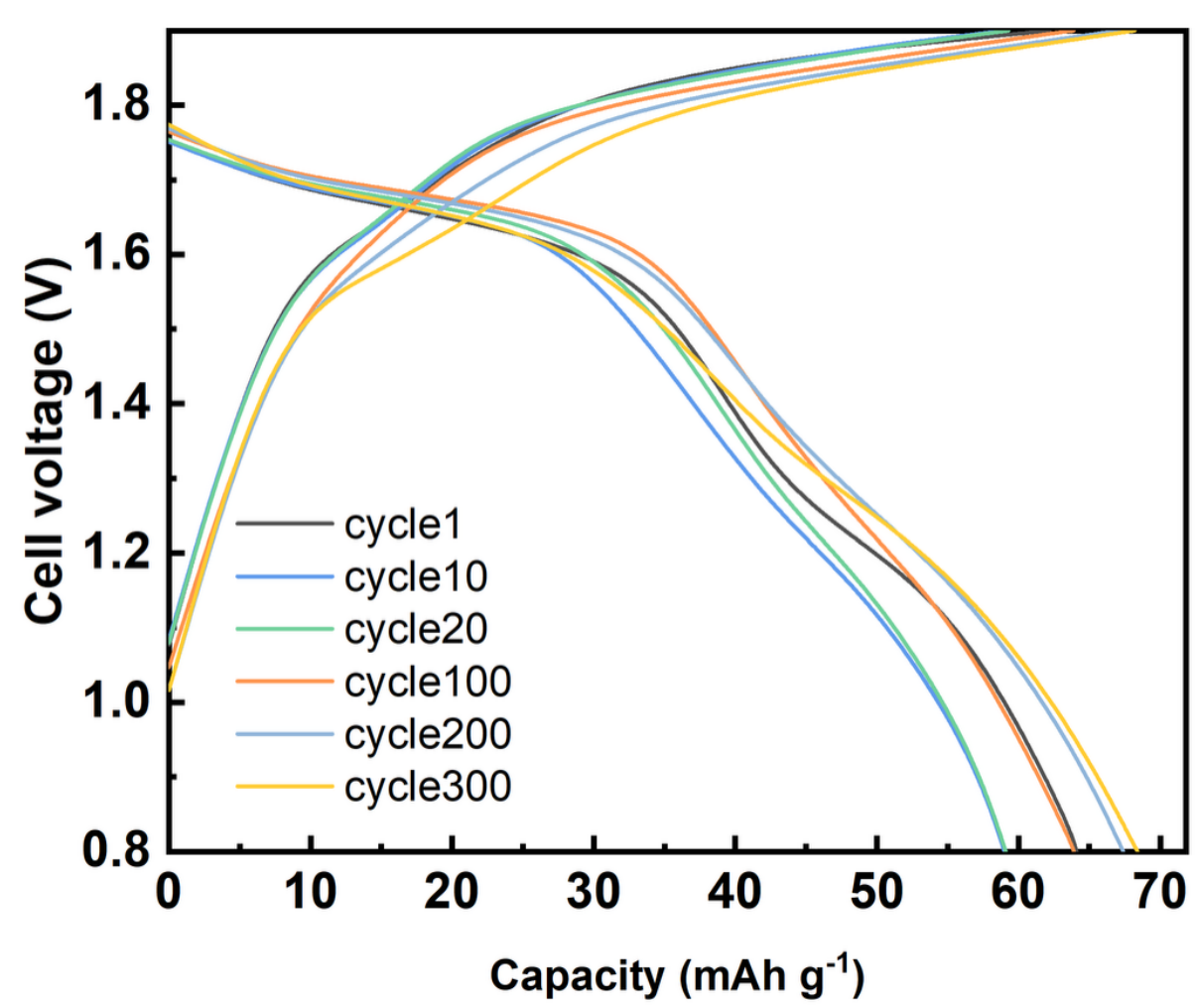

Supplementary Fig. 23. Voltage profiles of Zn|LMO full cell at a 4 C charge/discharge rate

7. To further substantiate Zn/ZnO coexistence, please include FFTs from the HRTEM ROIs, zone-axis identification, or simulated/standard FFT/diffraction overlays for wurtzite ZnO (P63mc) and hcp Zn (P63/mmc). Report the calibrated _d_-spacings with associated uncertainties used for phase indexing. Additionally, several figure panels and their captions appear mismatched in Figure 3, please reconcile panel labels and descriptions for consistency. Also, standardize the chemical notation by rewriting Li2SO4 with true subscripts (rather than pasted Unicode numerals) and applied this formatting consistently across the manuscript.

<u>Authors' Response:</u> We have revisited Figure 3 and the associated captions to more rigorously substantiate Zn/ZnO coexistence and to correct the formatting issues. Specifically:

(i) FFTs and zone-axis identification

We now include FFTs from the representative HRTEM ROIs (Fig. 3B and Supplementary Fig. 11A). The indexed reflections shown in Supplementary Fig. 11B-D and E-H are consistent with wurtzite ZnO ($P6_3mc$) and hcp Zn ($P6_3/mmc$), respectively, with zone-axis identification added in Supplementary Fig. 11D.

(ii) SAED overlays and d-spacings with uncertainty

We added three SAED patterns collected from different regions and overlaid the expected ring positions for ZnO with lattice planes labeled in the overlays (Supplementary Fig. 12). We also reported the calibrated d-spacings used for indexing, along with their uncertainties, and the standard-referenced values for transparency (Supplementary Table 3).

(iii) Figure/caption consistency and chemical notation

We reconciled the panel labels and caption descriptions throughout Fig. 3 to remove mismatches. We also standardized chemical notation throughout the manuscript (e.g., Li_2SO_4, D_2O) by consistently using true subscripts.

Manuscript edits:

Page 11:

Transmission electron microscopy (TEM) images (Figure 3A-B and Supplementary Fig. 11A) reveal a ~15 nm conformal SEI on the Zn metal surface after 3 plating/stripping cycles, exhibiting a characteristic core-shell structure in which the crystalline core is surrounded by an amorphous shell (Figure 3B-C and Supplementary Fig. 11A). Selected area electron diffraction (SAED, Figure 3D, Supplementary Fig. 12, and Table 3) and high-resolution TEM (HRTEM) analyses indicate the coexistence of ZnO (Figure 3E and Supplementary Fig. 11E-H) and metallic Zn (Figure 3F and Supplementary Fig. 11B-D).

Page 13:

Fig. 3 Incorporating Li_2SO_4 and DMF to elevate ionic transport and facilitate stable SEI formation. (A-B) TEM images of deposited Zn metal with a conformal SEI layer on the surface. (C) Schematic depiction of the amorphous SEI layer encapsulating the metallic Zn. (D) SAED pattern revealing ZnO and Zn. Lattice spacings of (E) ZnO and (F) Zn along the (002) direction.

Page S16:

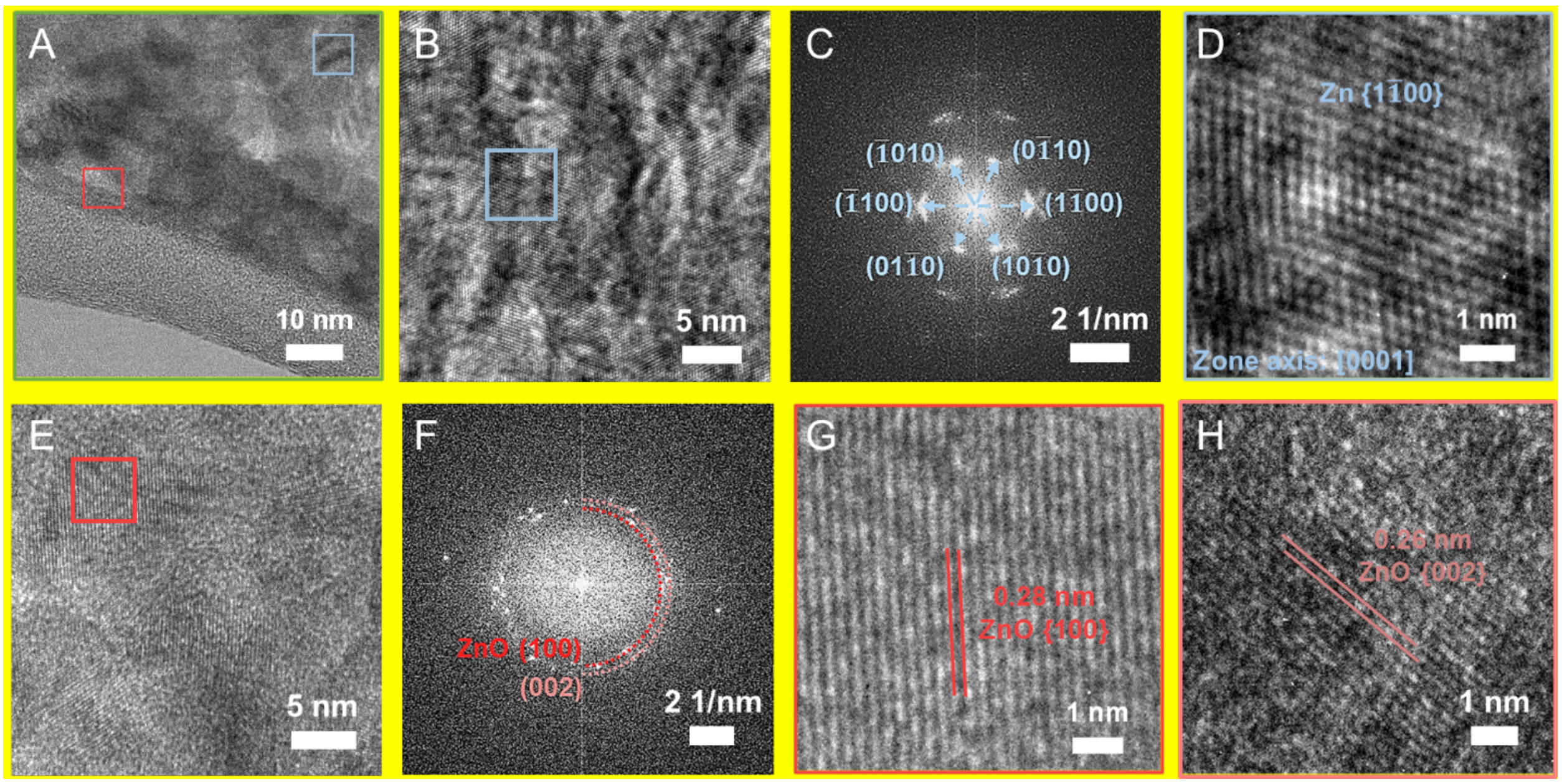

Supplementary Fig. 11. FFTs and zone-axis identification. FFTs and HETEM images from the representative (A) HRTEM ROIs (Fig. 3B). The indexed reflections in (B-C) and (E-H) are consistent with wurtzite ZnO ($P6_3mc$) and hcp Zn ($P6_3/mmc$), respectively, with zone-axis identification added in (D).

Page S17:

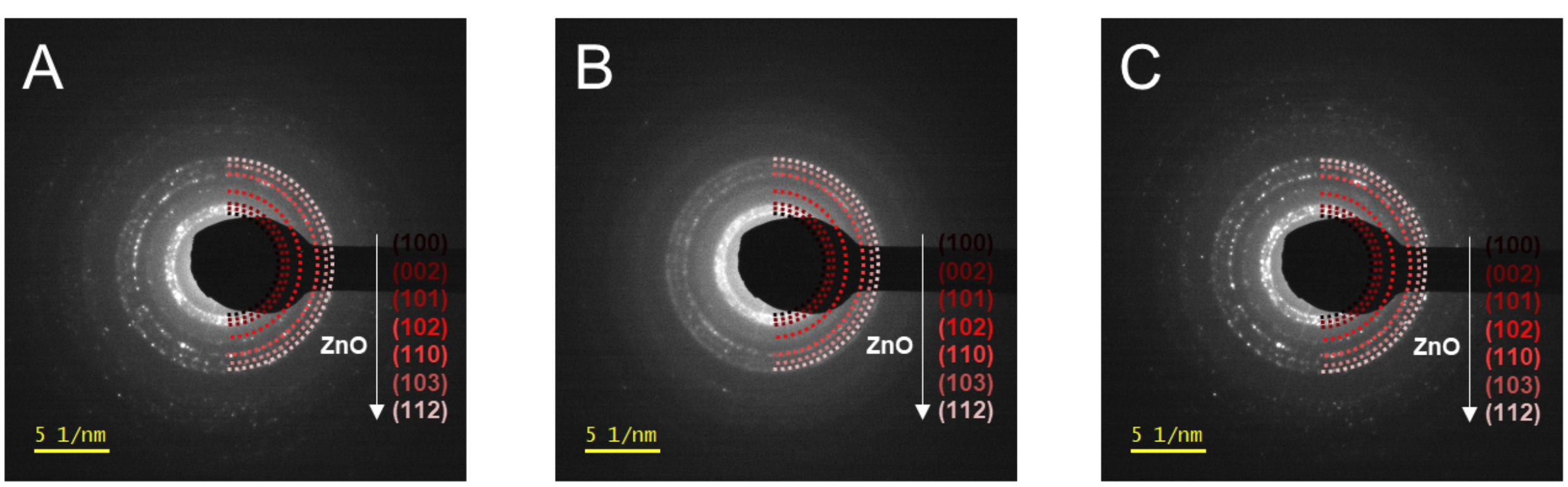

Supplementary Fig. 12. (A-C) Three SAED patterns collected from different regions and overlaid the expected ring positions for ZnO with lattice planes labeled in the overlays.

Page S34:

Supplementary Table 3. d-spacings with uncertainty measured from three SAED patterns in Supplementary Fig. 12 and the standard-referenced values.

Lattice plane (h k l)	d-spacing (Å, with uncertainties)	Standard d-spacing (Å)
**(100)**	**2.86 ± 0.01**	**2.81**
**(002)**	**2.63 ± 0.02**	**2.60**
**(101)**	**2.44 ± 0.03**	**2.48**
**(102)**	**1.87 ± 0.02**	**1.91**
**(110)**	**1.58 ± 0.01**	**1.63**
**(103)**	**1.45 ± 0.01**	**1.48**
**(112)**	**1.34 ± 0.01**	**1.38**

8. The significant S signal in the EDS maps (Fig. S9) suggests the possible formation of basic zinc sulfate (ZHS; Zn4SO4(OH)6×_x_H2O). To substantiate the hypothesis, DMF decomposes to (HCON(CH3)2) reacts initially with the Zn to form ZnCO3 and ZnO on the electrode surface, please provide surface-analytical evidence (XPS/ToF-SIMS/Raman) to distinguish ZHS from ZnCO3/ZnO and to confirm DMF-derived fragments within the interphase. What is the chemical identity, thickness evolution, and ion-transport property (Zn2+ transference, conductivity) of the DMF-derived SEI during long rest?

Authors' Response: We thank the reviewer for the comment regarding the chemical identity and stability of the interphase formed in our optimized electrolyte during calendar aging. In the revised manuscript, we therefore added XRD, XPS, EDS, TEM, EIS, and transference number characterizations to directly address these points.

(i) **Distinguishing ZHS from $ZnCO_3$/ZnO**

We note that the S signal in the EDS maps (Supplementary Fig. 13) could come from residual electrolyte salt (e.g., $ZnSO_4$, Li_2SO_4). To exclude the possibility and resolve this ambiguity, we performed high-resolution XPS after fully rinsing the aged/cycled Zn electrodes. In the XPS S 2p region (Supplementary Fig. 14C), we do not observe a detectable sulfate signal at the characteristic binding energy of sulfate (S $2p_{3/2}$ ~169 eV), which is commonly used to identify metal sulfates and sulfate-containing surface species *[Fuel 1990, 69, 8, 939-944]*. This absence indicates that ZHS is not a major constituent of the *surface* interphase under our conditions, within the XPS detection limit.

(ii) Confirmation of DMF-derived fragments within the interphase

In addition to ZnO evidence already shown by HRTEM in the manuscript (Fig. 3D-F), XPS reveals a carbonate-associated C 1s component consistent with carbonate species formation at 289.6 eV *[Surf. Interface Anal. 1991, 17, 3, 165-167; J. Spectrosc. 2018, 2079278, 14]*, supporting the presence of $ZnCO_3$ products (Supplementary Fig. 14A) as we proposed in the following equation:

$$Zn + Zn^{2+} + OH^{-} + HCON(CH_3)_2 + O_2 \rightarrow ZnCO_3 + ZnO + H_2N(CH_3)_2^{+}$$

We also detect an N-H bonding at 402.0 eV in the N 1s spectrum *[Polymers 2025, 17, 24, 3306; J. Phys. Chem. C 2014, 118, 1, 376-383]*, which is consistent with protonated amine-type species and supports the plausibility of DMF-derived nitrogenous products (i.e., $H_2N(CH_3)_2^{+}$) at the interface (Supplementary Fig. 14B). Collectively, we revised our manuscript to present the interface analysis with evidence for the presence of carbonate- and amine-containing products.

(iii) Chemical identity and SEI thickness evolution during long rest

To evaluate whether the interphase chemistry changes substantially during calendar aging, we compared EDS before and after aging (Fig. R6D, H). The Zn:O:S ratios remain similar (Zn:O:S ≈ 1:1.8:0.2 before aging vs. 1:2.3:0.2 after aging), suggesting that the sulfur fraction remains low and does not increase during aging, consistent with the XPS conclusion that sulfate-containing ZHS is not accumulating as a dominant surface phase. However, we found that the SEI becomes thicker and more heterogeneous after aging by TEM (Fig. R7), both before and after aging, indicating

that continuous corrosion of a carbonate/oxide-rich, DMF-involved interphase occurs within the interphase during long rest.

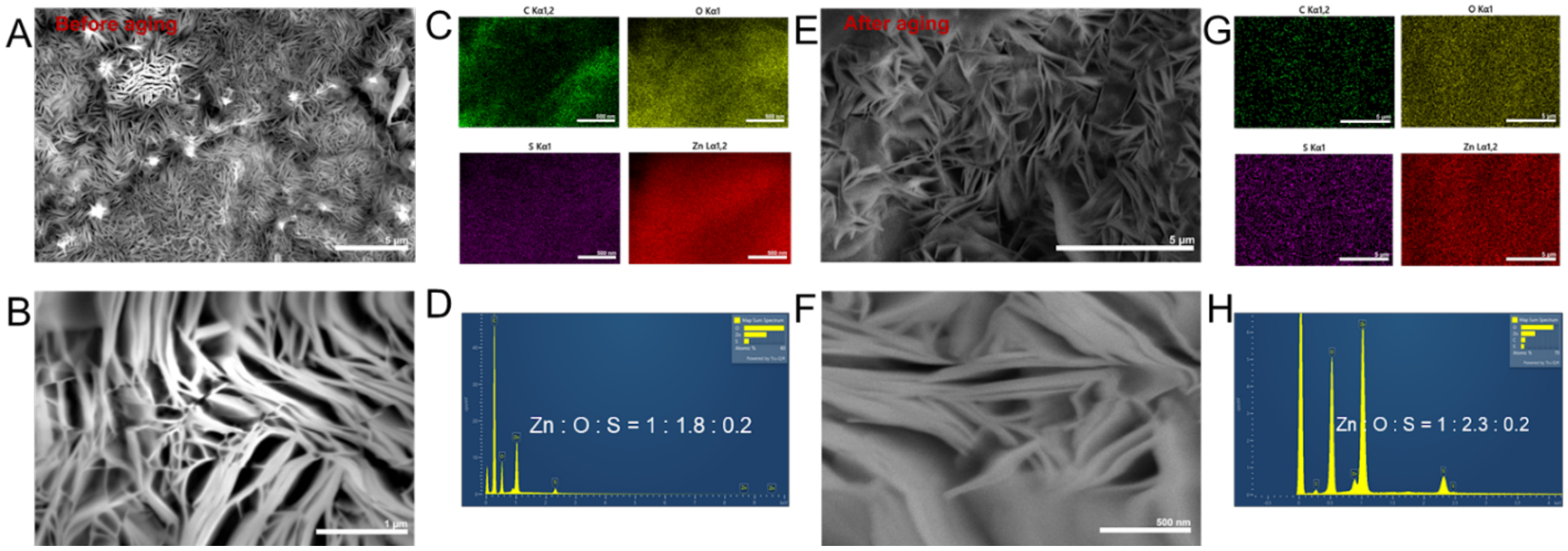

Fig. R6 SEM images of Zn deposits (A-B) before and (E-F) after aging, EDS mappings and quantitative elemental analysis (C-D) before and (G-H) after aging.

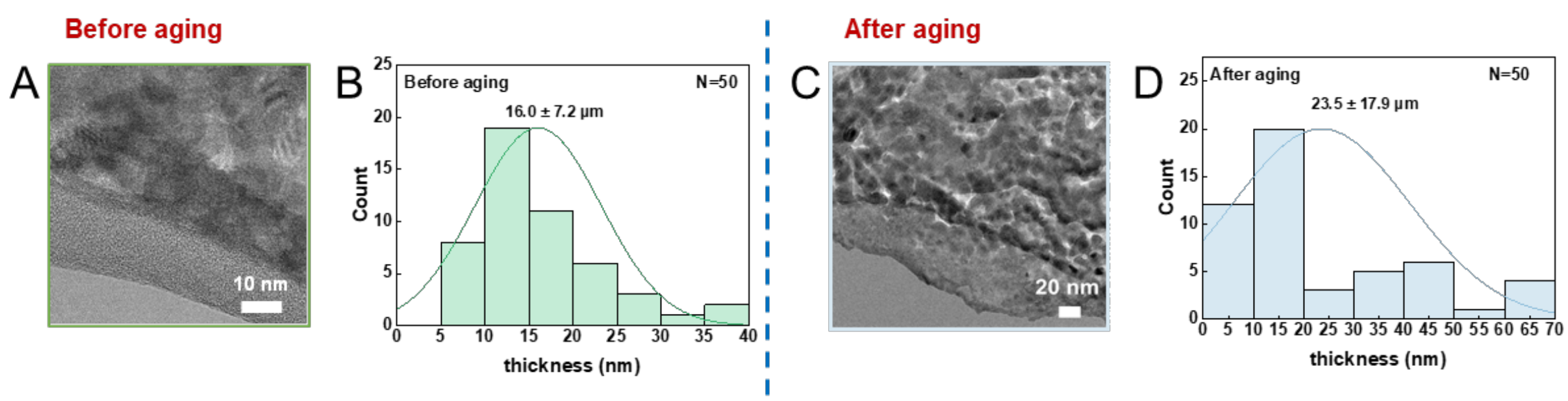

Fig. R7 TEM images of Zn deposits with statistical analysis of SEI thickness (A-B) before and (C-D) after aging.

(iv) Ion-transport property (transference number, conductivity) of the DMF-derived SEI during long rest

We measured the apparent transference number ($t_{+,app}$) in Zn||Zn symmetric cells using the protocol originally developed by Bruce and Vincent (see Method) and widely used as a comparative transport metric (Supplementary Fig. 8A-B) *[J. Am. Chem. Soc. 2022, 144, 17, 7583-7587; Polymer, Polymer 1987, 28, 13, 2324-2328]*. Because our electrolytes contain multiple ionic species (Zn^{2+}/Li^{+}/SO_4^{2-} and potentially solvated complexes), extracting a strict single-ion Zn^{2+}transference number would require more specialized treatments. Therefore, we report $t_{+,app}$ as a consistent comparative descriptor. Our optimized electrolyte shows substantially higher $t_{+,app}$ (0.32) than baseline

electrolytes (Supplementary Fig. 8C), consistent with improved effective cationic transport contribution in the presence of the DMF-enabled interphase and supporting salt.

In addition, we performed EIS measurements (Supplementary Fig. 7) before and after 24 h calendar aging. Nyquist plots were fitted with a standard equivalent circuit (Supplementary Fig. 7A), consistent with prior Zn battery EIS practice *[Nat. Commun. 2023, 14, 3067]*. From the fitting, we quantify $\Delta Rct = Rct\ (24\ h) - Rct\ (0\ h)$ (Supplementary Fig. 7H). The optimized electrolyte shows a much smaller ΔRct, indicating suppressed growth of interfacial polarization resistance, enabled by DMF-assisted interphase formation and the deuterated solvent environment during long rest.

Manuscript edits:

Page 10-11:

As shown in Supplementary Fig. 7, adding Li_2SO_4 substantially decreases the overall ionic transport resistance, offsetting the drawbacks of using an ultra-dilute Zn^{2+} without notably altering the reactivity of solvated water in the electrolyte.

Page 11-12:

XPS reveals a carbonate-associated C 1s component consistent with carbonate species formation at 289.6 eV,[38, 39] supporting the presence of $ZnCO_3$ products (Supplementary Fig. 13A). We also detect an N-H bonding at 402.0 eV in the N 1s spectrum,[40, 41] which is consistent with protonated amine-type species and supports the plausibility of DMF-derived nitrogenous products (i.e., $H_2N(CH_3)_2^+$) at the interface (Supplementary Fig. 13B).

Page 22-23:

The apparent transference number (t^+_{app}) was tested in Zn||Zn symmetric cells using the protocol originally developed by Bruce and Vincent and widely used as a comparative transport metric. Briefly, cells were rested for 12 h, then polarized at $\Delta V = 10$ mV for 1 h to reach steady state. Then, EIS was collected before/after polarization to obtain R_0 and R_{ss}, and t^+_{app} was calculated following the Bruce-Vincent framework:

$$t_+ = \frac{I_{ss}(\Delta V - I_0 R_0)}{I_0(\Delta V - I_{ss} R_{ss})}$$

Ionic conductivity (σ) was measured using a symmetric stainless-steel blocking-electrode configuration (SS|electrolyte|SS) assembled in CR2032 coin cells. A donut-shaped Celgard 2325 separator, saturated with electrolyte, was sandwiched between two stainless steel electrodes. The bulk ionic resistance (R_b) was extracted from the high-frequency intercept of the Nyquist plot obtained via EIS. Ionic conductivity was calculated as

$$\sigma = \frac{L}{R_b \times A}$$

Where L is the compressed separator thickness and A is the cross-sectional area defined by the separator geometry. While the absolute conductivity values are subject to uncertainty arising from this geometric approximation, all measurements were performed under identical conditions, ensuring internally consistent comparisons across the electrolyte formulations.

Page S12:

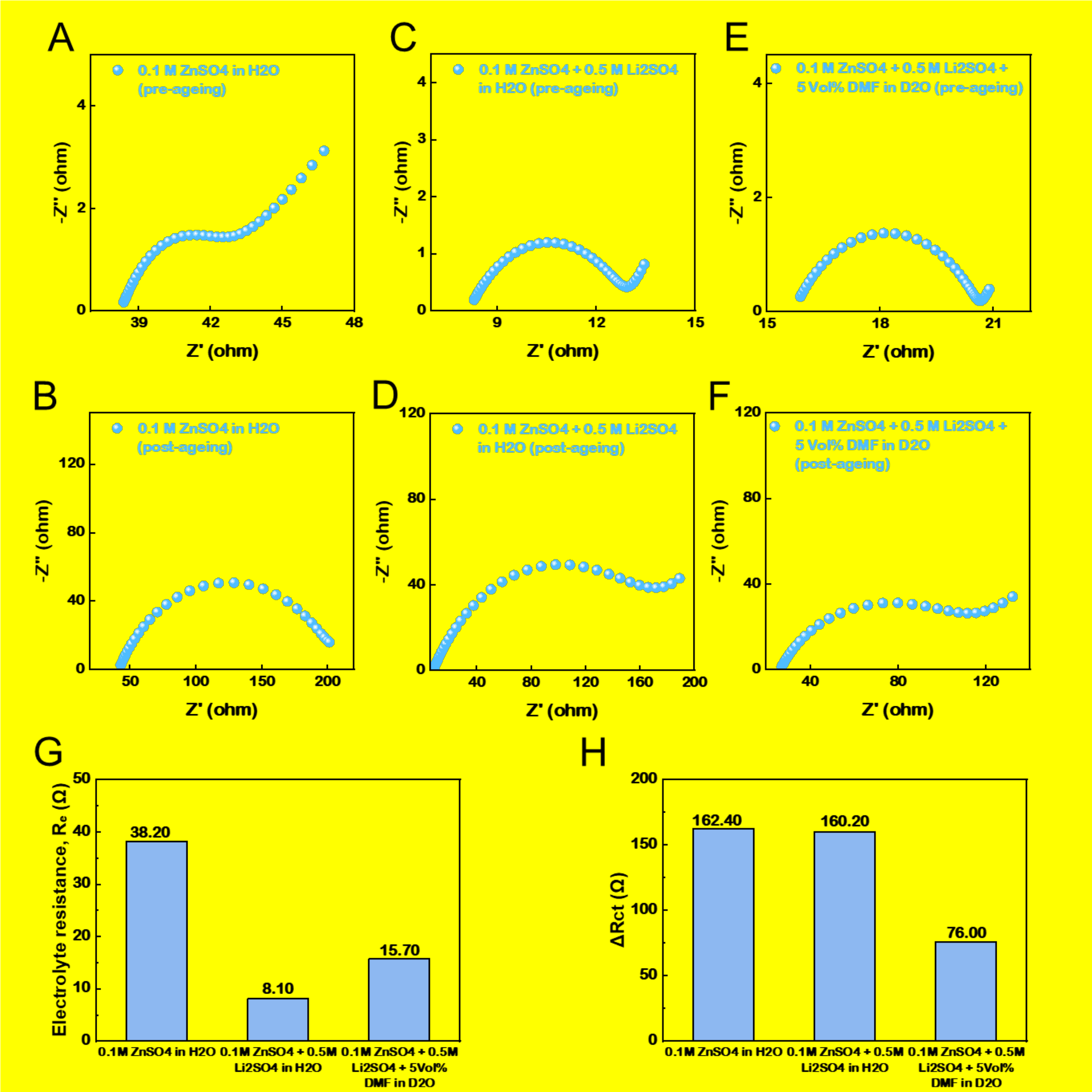

Supplementary Fig. 7. Cation concentration effects on electrolyte ionic conductivity. EIS profile before and after aging in (A-B) 0.1 M $ZnSO_4$ in H2O, (C-D) 0.1 M $ZnSO_4$ + 0.5 M Li_2SO_4 in H_2O, and (E-F) 0.1 M $ZnSO_4$ + 0.5 M Li_2SO_4 + 5 vol.% DMF in D_2O with equivalent circuit for fitting inset in (A). Summarized (G) electrolyte resistance and (H) aging induced charge transfer resistance growth.

Page S13:

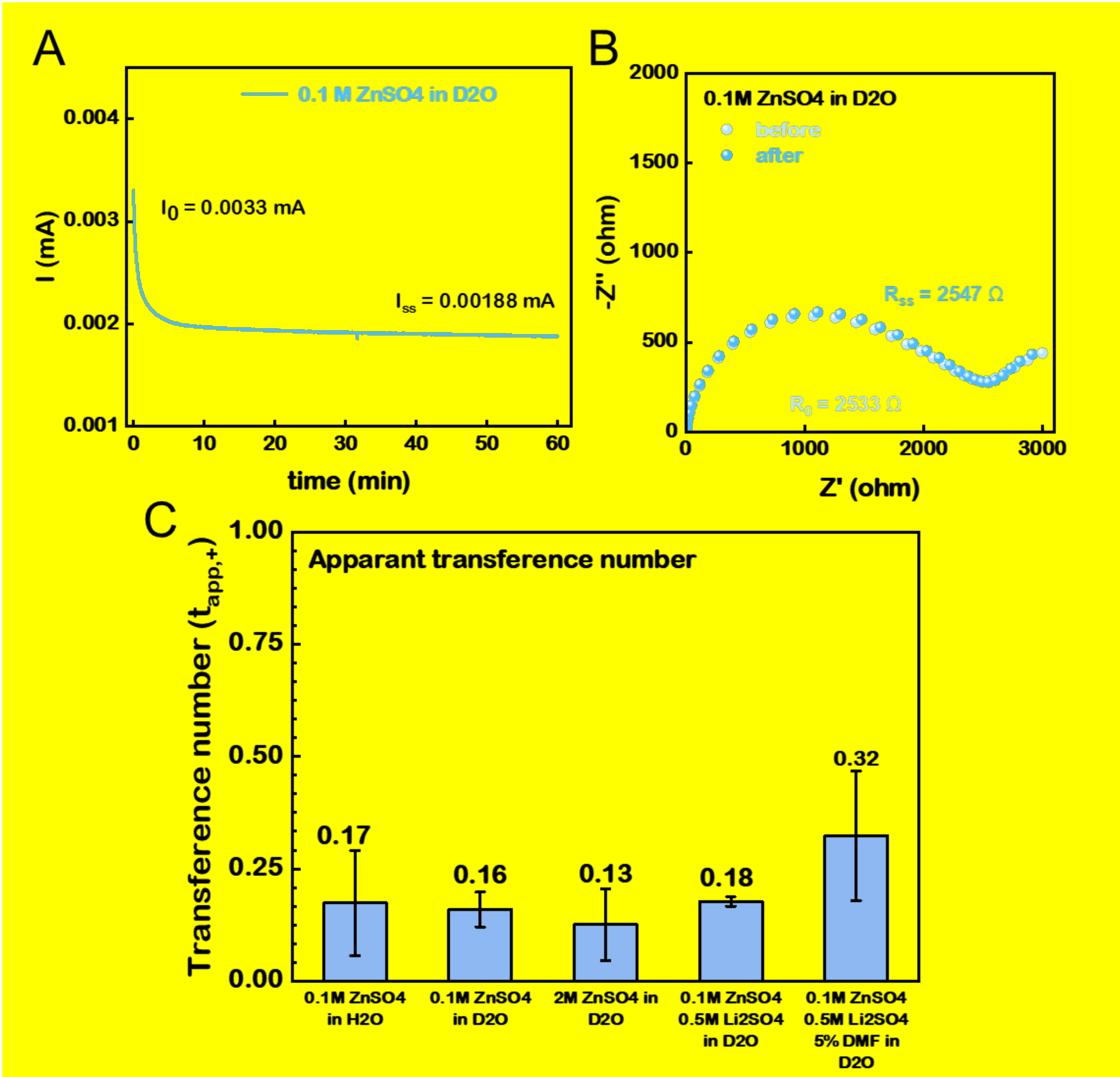

Supplementary Fig. 8. (A-B) Bruce Vincent method and (C) apparent transference number.

Page S19:

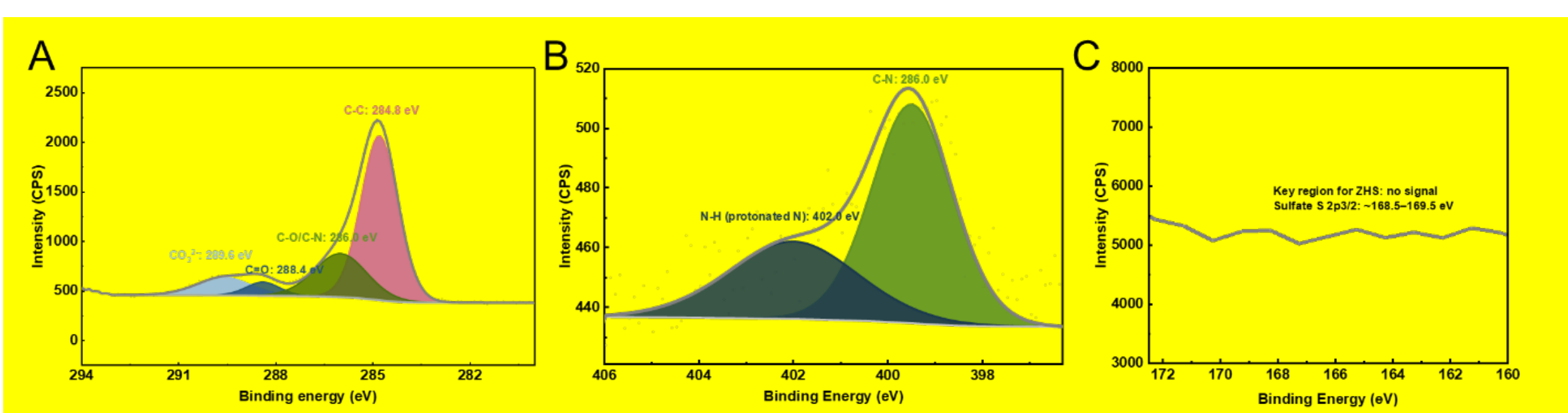

Supplementary Fig. 14. Post-cycling XPS analysis on the surface. (A) C 1s, (B) N 1s, and (C) S 2p.

9. Does the SEI mechanically survive storage? In-situ impedance during rest and post-rest morphology would clarify.

Authors' Response: We have added both in situ impedance measurements at rest and post-rest morphology characterizations to assess whether the SEI remains mechanically intact during calendar aging.

(i) In situ impedance measurements before and after ageing.

In the revised manuscript, we added EIS tracking during the 24 h OCV rest period (Supplementary Fig. 7). Our optimized electrolyte shows suppressed growth of interfacial polarization resistance during rest, as reflected by the much smaller increase in fitted charge-transfer resistance compared to baseline electrolytes (summarized as ΔRct in Supplementary Fig. 7E). In addition, the electrolyte resistance (summarized as R_e in Supplementary Fig. 7D) remains low, indicating that the interphase formed in our electrolyte does not introduce a severe transport bottleneck during storage. Together, the limited growth in impedance supports the conclusion that the SEI remains stable rather than progressively blocking ion transport during storage.

(ii) Post-rest morphology characterizations.

We further performed SEM before and after calendar aging. Post-rest electrodes retain an intact and layered morphology without evidence of collapse or pulverization (Fig. R6A-B, E-F). Complementary TEM images (Fig. R7) show that the interphase becomes thicker and more heterogeneous after rest but remains continuous rather than showing serious cracks to expose fresh Zn surface.

Overall, the combined evidence supports the conclusion that the SEI formed in our optimized electrolyte remains mechanically intact during storage and suppresses the growth of interfacial polarization during calendar aging.

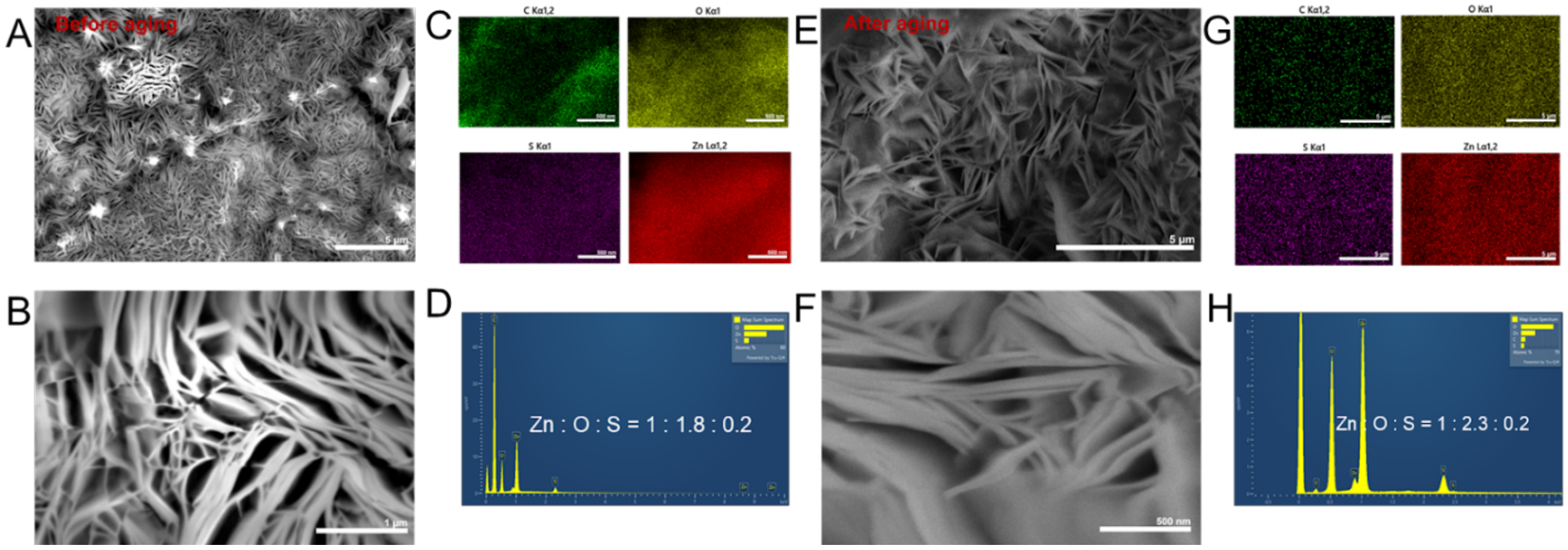

Fig. R6 SEM images of Zn deposits (A-B) before and (E-F) after aging, EDS mappings and quantitative elemental analysis (C-D) before and (G-H) after aging.

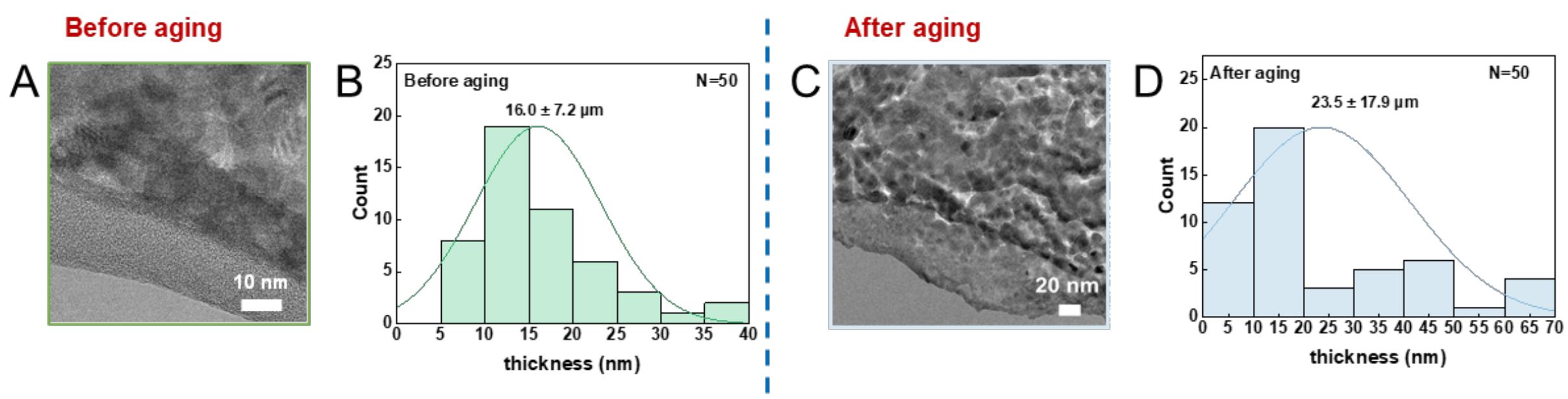

Fig. R7 TEM images of Zn deposits with statistical analysis of SEI thickness (A-B) before and (C-D) after aging.

Manuscript edits:

Page 10:

As shown in Supplementary Fig. 7, adding Li_2SO_4 substantially decreases the overall ionic transport resistance, offsetting the drawbacks of using an ultra-dilute Zn^{2+} without notably altering the reactivity of solvated water in the electrolyte.

Page 22-23:

Ionic conductivity (σ) was measured using a symmetric stainless-steel blocking-electrode configuration (SS|electrolyte|SS) assembled in CR2032 coin cells. A donut-shaped Celgard 2325 separator, saturated with electrolyte, was sandwiched between two stainless steel electrodes. The bulk ionic resistance (R_b) was extracted from the high-frequency intercept of the Nyquist plot obtained via EIS. Ionic conductivity was calculated as

$$\sigma = \frac{L}{R_b \times A}$$

Where L is the compressed separator thickness and A is the cross-sectional area defined by the separator geometry. While the absolute conductivity values are subject to uncertainty arising from this geometric approximation, all measurements were performed under identical conditions, ensuring internally consistent comparisons across the electrolyte formulations.

Page S12:

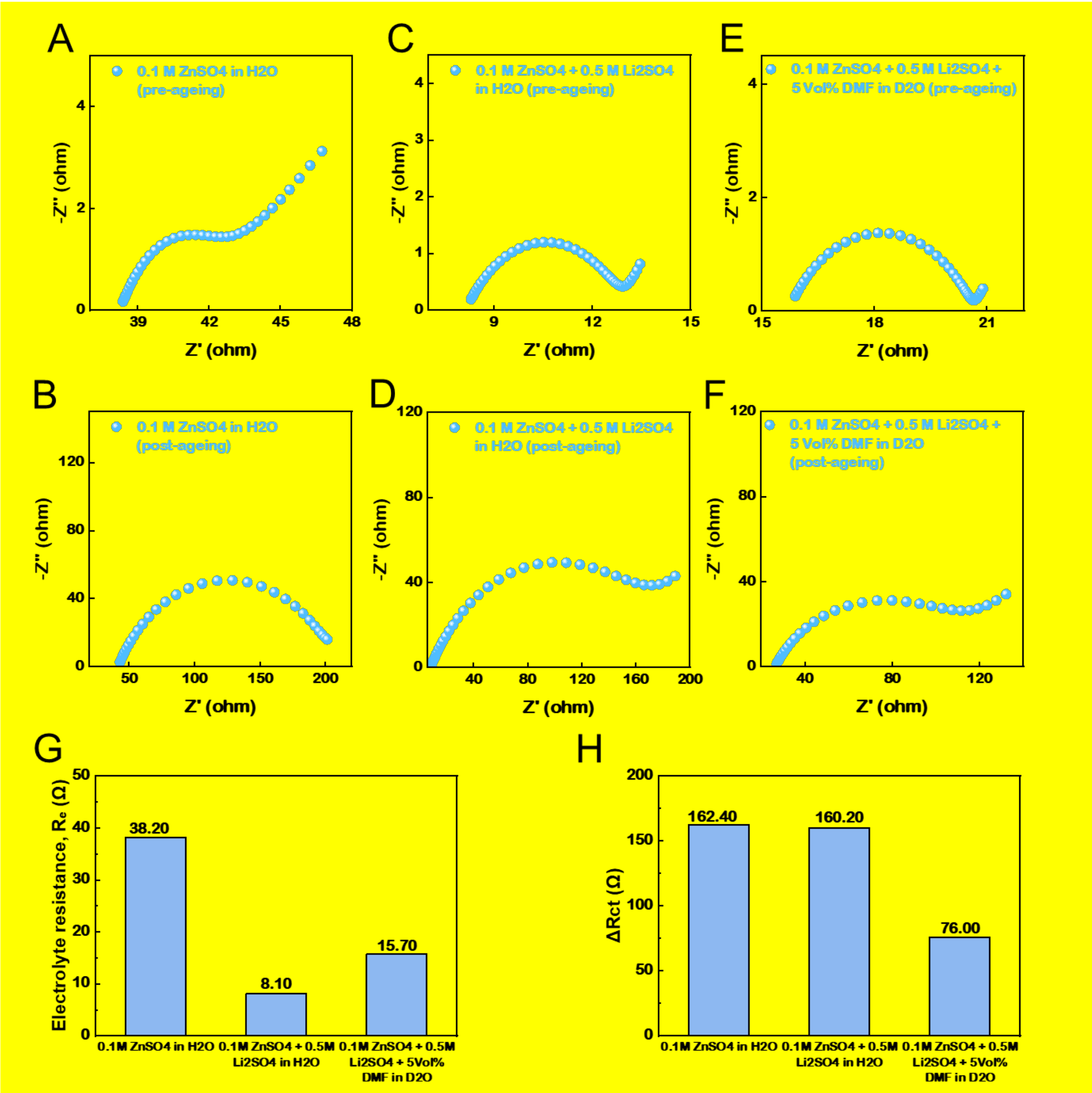

Supplementary Fig. 7. Cation concentration effects on electrolyte ionic conductivity. EIS profile before and after aging in (A-B) 0.1 M $ZnSO_4$ in H2O, (C-D) 0.1 M $ZnSO_4$ + 0.5 M Li_2SO_4 in H_2O, and (E-F) 0.1 M $ZnSO_4$ + 0.5 M Li_2SO_4 + 5 vol.% DMF in D_2O with equivalent circuit for fitting inset in (A). Summarized (G) electrolyte resistance and (H) aging induced charge transfer resistance growth.

10. The pH rises as concentration falls, and capacity retention tracks pH. Is corrosion suppression pH-driven or solvation-driven? Please quantify how does the pH (bulk and interfacial) influence Zn2+ primary/secondary solvation structure, _e.g._, H-bonding signatures, and link to corrosion/HER metrics.

<u>Authors' Response:</u> We thank the reviewer for this insightful question. **We first clarify that pH is not an independent "knob" here, but rather an indicator of the Zn^{2+}–H2O solvation activity**. In $ZnSO_4$ electrolytes, the measured bulk pH is governed primarily by Zn^{2+}-water chemistry, i.e., the extent to which water molecules reside in the solvation shell and become activated toward proton donation. Specifically, at higher Zn^{2+} concentration, a larger fraction of water is coordinated around Zn^{2+} and can undergo hydrolysis reactions that release H^+, yielding a more acidic bulk pH. As Zn^{2+} concentration decreases, the population of Zn^{2+}-coordinated (solvated) water decreases, and the electrolyte pH approaches ~7. We therefore treat bulk pH as a quantitative reporter of solvation activity, indicating the extent of solvated water, rather than as the sole causal origin of corrosion suppression.

The key question the reviewer raised is whether improved stability of corrosion/HER suppression is attributable to *higher pH* or to *reduced reactive solvated water*. We emphasize that **corrosion/HER suppression correlates most directly with solvated water reactivity, with pH tracking this same underlying change, and would like to** strengthen the mechanistic link between bulk pH, solvation signatures, and corrosion/HER metrics by explicitly linking the following three experimental aspects:

(i) Corrosion/HER kinetics (Tafel analysis)

The corrosion kinetics trend, as shown in the Tafel plot analysis (Fig. 2F and Supplementary Fig. 4), systematically decreases as Zn^{2+} concentration is reduced. This is also consistent with our mechanistic picture that the dominant proton-

donating population is **Zn^{2+}-coordinated water**, whose O–H bonding energy is weakened relative to bulk water based on the DFT calculations (Fig. 2A-B and Supplementary Fig. 1), making it more susceptible to cleavage and thus accelerating HER/corrosion at the Zn surface.

(ii) **Bulk-solvation signatures (FTIR)**

We expand the analysis in Supplementary Fig. 2 by comparing the OH-stretch region across different concentrations in $ZnSO_4$-based electrolytes. The concentration-dependent changes in the broad OH-stretch features and the attenuation of strongly perturbed water signatures upon dilution provide a spectroscopic proxy that dilution reduces the fraction of strongly solvated water and restores a more bulk-like water population, consistent with the observed pH rise and reduced corrosion kinetics.

In addition, we agree that local interfacial pH can deviate from the bulk due to HER-driven OH^- generation and transport limitations. Quantitative interfacial pH mapping would require specialized techniques (e.g., Scanning electrochemical microscopy (SECM) and micro-pH probes). Due to instrument limitations, we currently cannot reliably quantify interfacial effects and use them as solvation-activity indicators. However, we acknowledge the importance of interfacial pH measurements and agree that it will be a key direction for future work.

11. Because ultra-dilute electrolytes are prone to Zn2+ depletion and dendrite formation, it would strengthen the case to include operando imaging confirming uniform deposition at application-relevant current densities/areal capacities. Please show how Li2SO4 and the SEI shift these metrics.

Authors' Response: We appreciate the reviewer's point that including operando visualization would be a strong way to validate uniform deposition under application-relevant conditions. As the optical operando imaging instrument is under repair in our lab, we performed cross-sectional FIB-SEM as a practical surrogate to directly assess through-thickness morphology, compactness, and uniformity of Zn deposits formed at a high current density and areal capacity (20 mA cm^{-2}, 3 mAh cm^{-2}). As shown in Supplementary Fig. 10, the conventional 2 M $ZnSO_4$ electrolyte shows highly non-uniform and protruding deposits with micron range (~2.5–6.1 μm) thickness. In

contrast, under the same plating conditions, our electrolyte yields much thinner, denser, and more laterally uniform Zn deposits without the large needle-like protrusions observed, with measured thicknesses in the sub-micron regime (~0.26–0.48 μm). These results, compared with a conventional 2 M $ZnSO_4$ electrolyte, suggest that Li_2SO_4 substantially reduces the bulk transport resistance, thereby alleviating concentration polarization and delaying interfacial Zn^{2+} depletion under high-rate plating. On the other hand, DMF, as an additive, provides a more controlled interfacial environment that promotes more homogeneous Zn nucleation and growth.

Manuscript edits:

Page 11:

To fulfill this role, we selected low concentration, 5 vol%, DMF as an effective choice to promote in situ SEI formation guided by earlier studies,[37] where DMF acts as an additive to make a compact Zn morphology (Supplementary Fig. 9 and 10).

Page 23:

Focused-ion-beam scanning electron microscopy (FIB-SEM, Nova 600) was performed using a Ga^+ source. Electron imaging was carried out at 10 kV and 0.54 nA, while ion milling was conducted at 30 kV, using 5 nA for trench cutting and 0.5 nA for final surface cleaning.

Page S15:

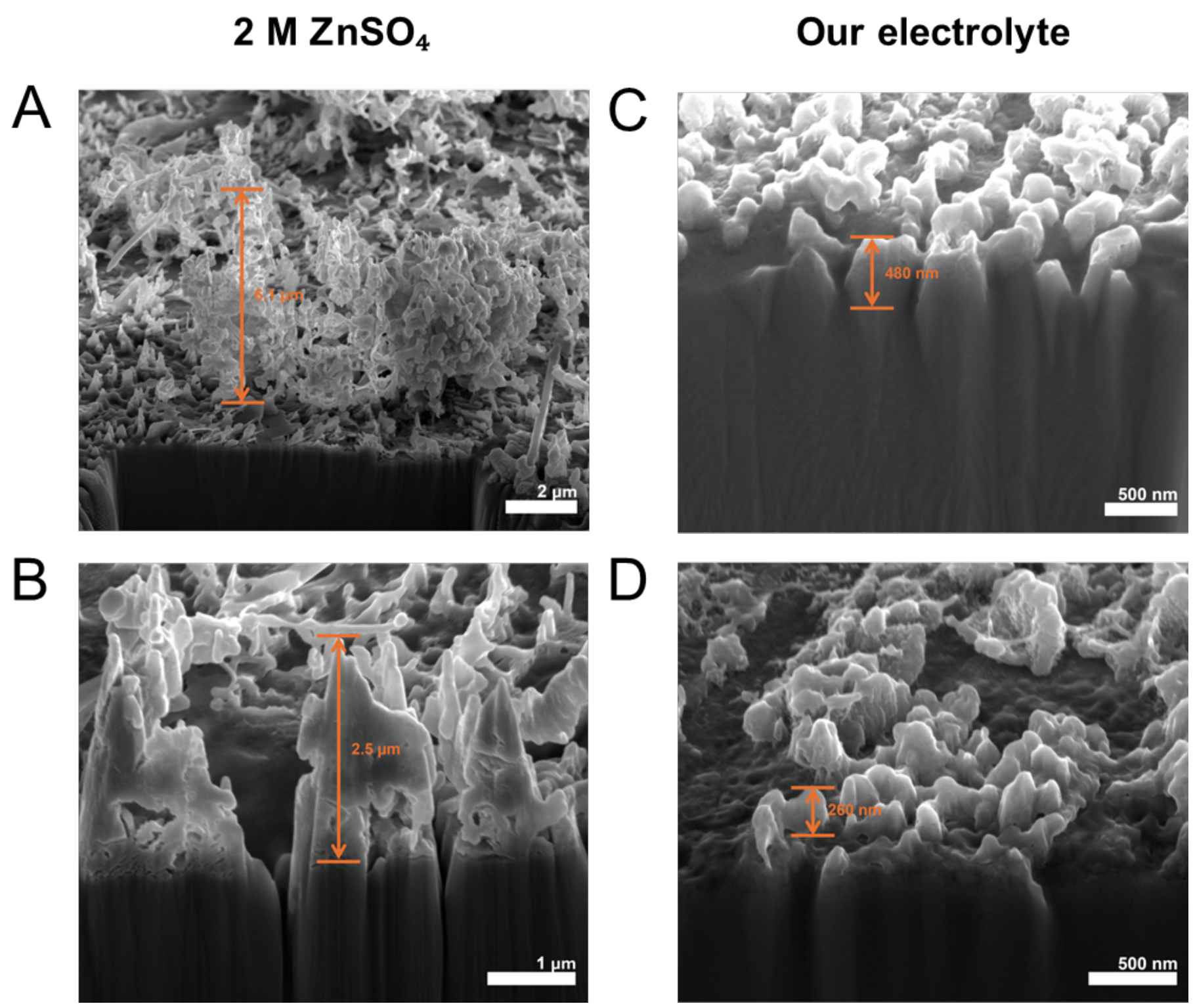

Supplementary Fig. 10. FIB-SEM cross-sectional images. (A-B) 2 M $ZnSO_4$ and (C-D) our optimized electrolyte.

12. Fig. 1A contains a typographical error: the term should read 'water-in-salt' electrolyte.

<u>Authors' Response:</u> We sincerely appreciate the reviewer pointing out this mistake. We have revised the typo in the manuscript.

Manuscript edits:

Page 5:

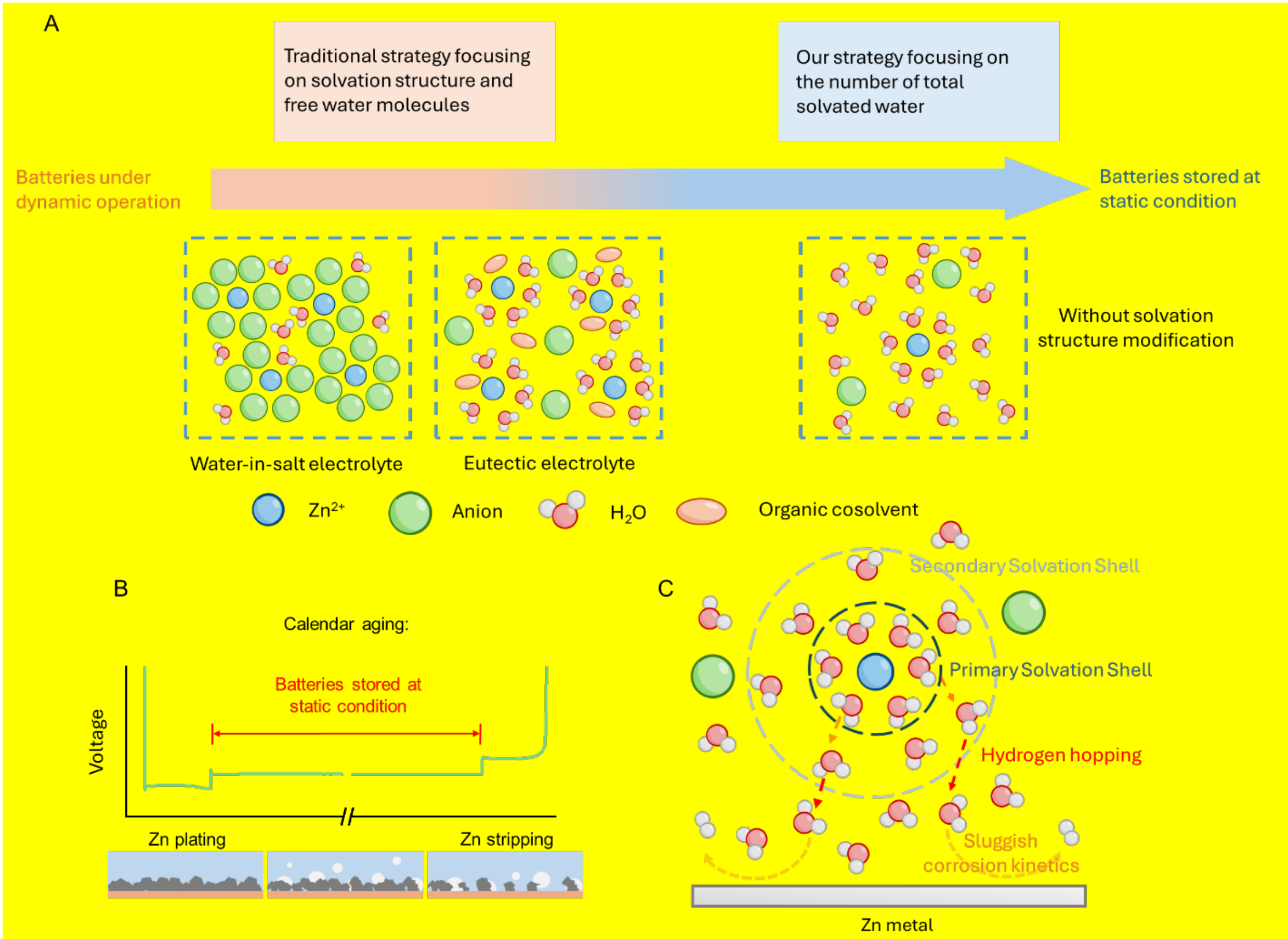

Fig. 1: Schematics of electrolyte design principle between traditional electrolyte and our electrolyte, calendar aging process, and Zn^{2+} solvation structure. (A) Design principle on solvation structure. (B) Chemical corrosion and "dead Zn" formation during 24-h calendar aging lead to colossal CE loss.[8] (C) Schematic illustration showing how an ultra-dilute electrolyte reduces Zn metal corrosion during calendar aging by minimizing the amount of solvated water molecules.

13. Regarding TGC analysis, more detailed information must be provided on the assumptions and formula to assign the measured signal to Zn0 and Zn2+ proportions.

Authors' Response: TGC was conducted in accordance with prior work in our group *[ACS Energy Lett. 2023, 8, 9, 3820-3828]*, which can be detailed as follows:

(1) Cu foils, together with the separators near the Cu foil side with inactive Zn species on top in 5 Zn|Gr half cells, were collected and put into a 12.5 mL vial without washing.

(2) The container was then sealed with a rubber septum and further capped by a stainless-steel ring to minimize the deformation of the rubber septum for safety when gas was generated later in an Ar-filled glovebox (O2 < 0.2 p.p.m., H2O < 0.02 p.p.m.).

(3) After transferring the container from the glovebox, a proper amount of hydrochloric acid (HCl, Fisher Scientific, 36.5 to 38.0%) was injected into the container to react with the residual Zn metal (Zn^0) completely, forming hydrogen gas (H_2) products.

(4) The as-generated gas was prevented from accumulating on top of the container by shaking. A gas-tight syringe was used to quickly take 50 μL of the well-mixed gas from the sealed container into the GC system for H_2 measurement. TGC collected three data points at each measurement point to ensure reliable results.

Key assumptions and formulas include:

(1) All metallic Zn^0 residues react completely with injected HCl in the sealed vial as follows

$$Zn^0 + 2HCl \rightarrow ZnCl_2 + H_2$$

(2) The generated H_2 is fully retained and well-mixed before sampling (we shake the vial to avoid gas stratification).

(3) Electrochemical capacity is converted to Zn mass using Zn's theoretical specific capacity of 819 mAh/g, or equivalently, 1 mAh Zn means 1.221 mg Zn.

(4) The number of Zn metal mass within the container (mZn) versus H2 peak area is based on the relationship 1 p.p.m. = 4.08×10^{-8} mmol mL^{-1} at 1 atm and 298 K.

We have added the above assumptions and equations explicitly to the revised Methods section to make the Zn^0 vs. Zn^{2+} assignment clear.

Manuscript edits:

Page 24:

To assign the measured signal to Zn^0 and Zn^{2+} proportions, we have the following key assumptions and formulas:

(1) All metallic Zn^0 residues react completely with injected HCl in the sealed vial as follows:

$$Zn + 2HCl \rightarrow ZnCl_2 + H_2$$

(2) The generated H_2 is fully retained and well-mixed before sampling (we shake the vial to avoid gas stratification).

(3) Electrochemical capacity is converted to Zn mass using Zn's theoretical specific capacity of 819 mAh g^{-1}, or equivalently, 1 mAh Zn means 1.221 mg Zn.

(4) The number of Zn metal mass within the container (m_{Zn}) versus H_2 peak area is based on the relationship 1 p.p.m. = 4.08×10^{-8} mmol mL^{-1} at 1 atm and 298 K.

Response to Reviewer #4:

I co-reviewed this manuscript with one of the reviewers who provided the listed reports. This is part of the Nature Communications initiative to facilitate training in peer review and to provide appropriate recognition for Early Career Researchers who co-review manuscripts.

Authors' Response: We sincerely appreciate the reviewer's contribution and thank them for their thoughtful evaluation of our work.

Response to Reviewer #5:

This manuscript presents a strategy to significantly suppress capacity fade of Zn metal during static storage through the design of an ultra-low concentration ZnSO4 electrolyte (0.1 M), while maintaining excellent cycling performance. The work identifies solvated water molecules as the primary driver of corrosion and demonstrates that a combination of reduced salt concentration, isotopic solvent substitution, and targeted additives can simultaneously mitigate static decay and enhance cycling stability, offering a promising approach for long-lived metal-anode batteries. However, in view of the construction of electrolyte, there are still some shortcomings in the study of the mechanism of improving the performance of the manuscript in the comparison group and the specific components, and a major revision is required.

Authors' Response: We thank the reviewer for the overall assessment and for recognizing the practical significance of suppressing Zn calendar-aging fade using an ultra-dilute $ZnSO_4$ electrolyte while maintaining long-term cycling stability. We take the opportunity to address the reviewer's concerns and improve the clarity of our work.

1. In order to clarify the enhancement of each component in the modified electrolyte system for corrosion resistance and aging mitigation, respectively, the reviewers suggested the authors to supplement the corresponding test results of the 2 M $ZnSO_4$+(0.5 M Li_2SO_4/5 vol% DMF in D_2O)-H_2O system.

Authors' Response: To address this point, we performed several control experiments using the following four electrolytes: (1) 2 M $ZnSO_4$ in H_2O, (2) 2 M $ZnSO_4$ + 0.5 M Li_2SO_4 in H_2O, (3) 2 M $ZnSO_4$ + 0.5 M Li_2SO_4 + 5 vol% DMF in H_2O, and (4) 2 M $ZnSO_4$ + 0.5 M Li_2SO_4 + 5 vol% DMF in D_2O. For each formulation, we conducted EIS before and after a 24 h calendar aging (Fig. R8A-H) and extracted the charge transfer resistance (ΔRct) induced by the calendar aging process after fitting to quantitatively show their aging mitigation effect (Fig. R8I). Overall, calendar aging results in increased interfacial polarization in all cases, as evidenced by the increase in the fitted Rct after 24 h. Quantitatively, the baseline electrolyte (2 M $ZnSO_4$ in H_2O) shows ΔRct = 223.9 Ω, while adding Li_2SO_4 alone gives a similar ΔRct = 215.8 Ω, indicating that Li_2SO_4 by itself provides limited interfacial stabilization against aging-induced impedance growth. This is

consistent with our interpretation that Li_2SO_4 primarily serves as a supporting salt to reduce bulk transport resistance rather than directly suppress corrosion-driven interfacial degradation. In contrast, once DMF is introduced, ΔRct decreases substantially to 149.2 Ω, demonstrating that DMF is the dominant contributor to mitigating aging-induced buildup of interfacial resistance, owing to its role in enabling a protective SEI-like interphase. Notably, replacing H_2O with D_2O further reduces ΔRct to 114.8 Ω. These stepwise controls support the distinct roles of each component under the 2 M baseline: **Li_2SO_4 improves transport**, **DMF provides interphase protection, and D_2O provides additional corrosion suppression by reducing solvent reactivity.**

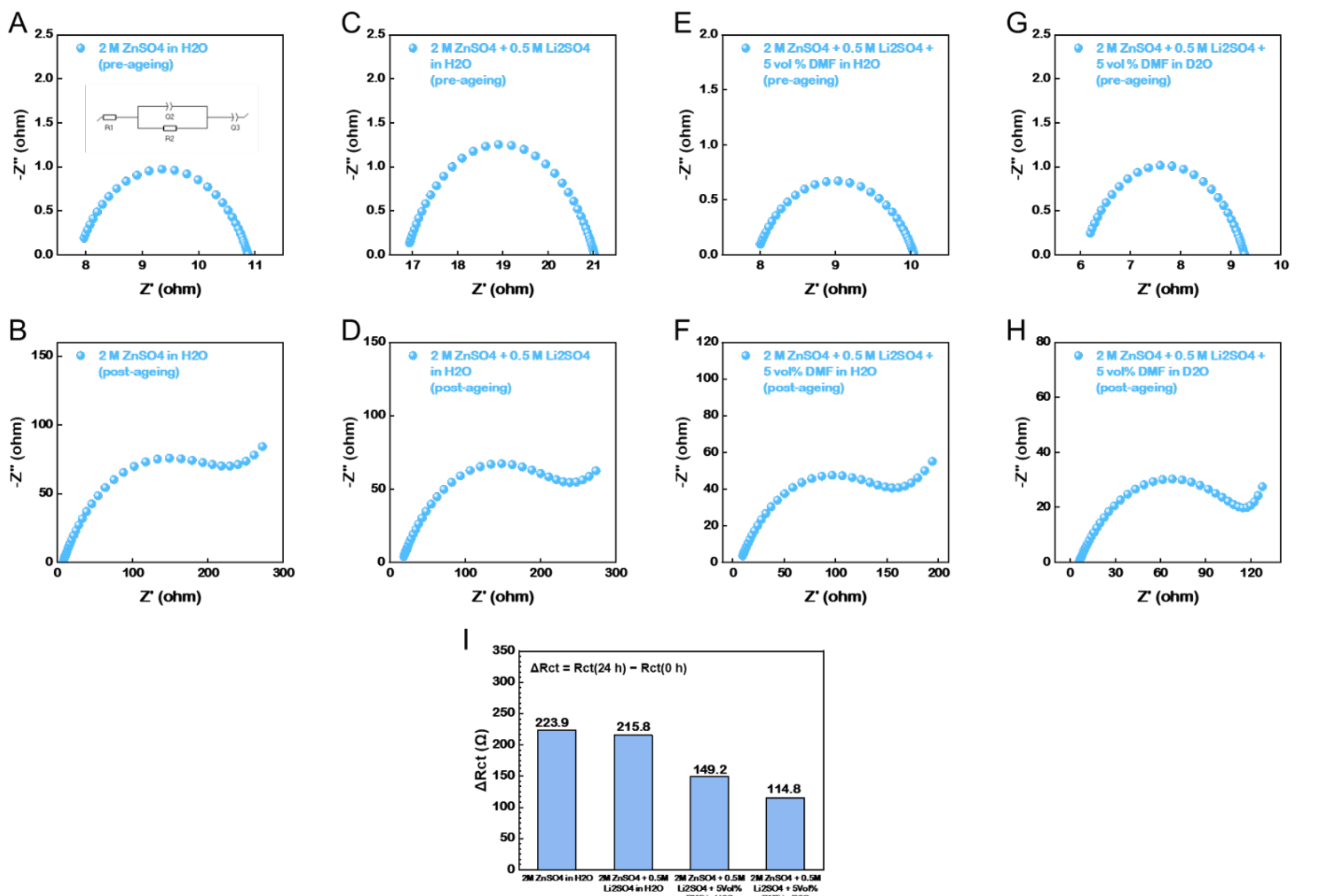

Fig. R8 EIS profile before and after aging in (A-B) 2 M $ZnSO_4$ in H_2O, (C-D) 2 M $ZnSO_4$ + 0.5 M Li_2SO_4 in H_2O, (E-F) 2 M $ZnSO_4$ + 0.5 M Li_2SO_4 + 5 vol.% DMF in H_2O, (G-H) 2 M $ZnSO_4$ + 0.5 M Li_2SO_4 + 5 vol.% DMF in D_2O with an equivalent circuit for fitting inset in (A). Summarized (I) aging-induced charge transfer resistance growth.

Manuscript edits:

Page 22-23:

Ionic conductivity (σ) was measured using a symmetric stainless-steel blocking-electrode configuration (SS|electrolyte|SS) assembled in CR2032 coin cells. A donut-shaped Celgard 2325 separator, saturated with electrolyte, was sandwiched between two stainless steel electrodes. The bulk ionic resistance (R_b) was extracted from the high-frequency intercept of the Nyquist plot obtained via EIS. Ionic conductivity was calculated as

$$\sigma = \frac{L}{R_b \times A}$$

Where L is the compressed separator thickness and A is the cross-sectional area defined by the separator geometry. While the absolute conductivity values are subject to uncertainty arising from this geometric approximation, all measurements were performed under identical conditions, ensuring internally consistent comparisons across the electrolyte formulations.

2. T haviour within the electrolyte at different temperatures, different undesirable phenomena affecting ion transport can occur. The reviewer suggests adding the possible anti-aging behaviour of electrolyte systems below 4°C under conditions of ice crystal appearance. At the same time, a specific analysis of the modification of the electrolyte for the modulation of hydrogen bonding behaviour and dissolution sheaths at different temperatures should be added.

Authors' Response: We agree that, if ice crystallization occurs, phase separation and freeze-concentration can locally amplify salt concentration and alter ion transport, complicating interpretation. Therefore, we added a 24 h calendar aging test at 0 °C (Supplementary Fig. 18) and found the cell remained electrochemically operable, exhibiting a stable voltage profile. It delivered a Coulombic efficiency of 97.3% after calendar aging, demonstrating that the electrolyte system remains effective for suppressing aging even under near-freezing conditions (below 4°C).

At the same time, to further confirm the solvation structure regulation and the hydrogen bonding behavior at different temperatures, we conducted Tafel analyses at 0, 4, 25, and 45 °C to compare 2 M $ZnSO_4$ in H_2O and 2 M $ZnSO_4$ + 0.5 M Li_2SO_4 + 5 vol% DMF in D_2O (Supplementary Fig. 4D). The electrolyte with additive and solvent modifications exhibits a lower corrosion current density across different temperatures, highlighting DMF-assisted SEI formation with solvation modulation and reduced HER kinetics in D_2O (kinetic isotope effect) that protects Zn during calendar aging.

Overall, these data provide quantitative, temperature-resolved confirmation that the modified electrolyte suppresses corrosion/HER kinetics across a broad range of temperatures, including below 4 °C.

Manuscript edits:

Page 14-15:

Under lower-temperature conditions (0 and 4 °C), the Zn anode demonstrates reversible capacities of 97.3% and 98.8% with only 2.7% and 1.2% capacity loss during calendar aging, respectively, demonstrating that the electrolyte system remains effective for suppressing aging even under near-freezing conditions, which is the best aging performance reported to date (Figure 4E and Supplementary Fig. 17).

Page S9:

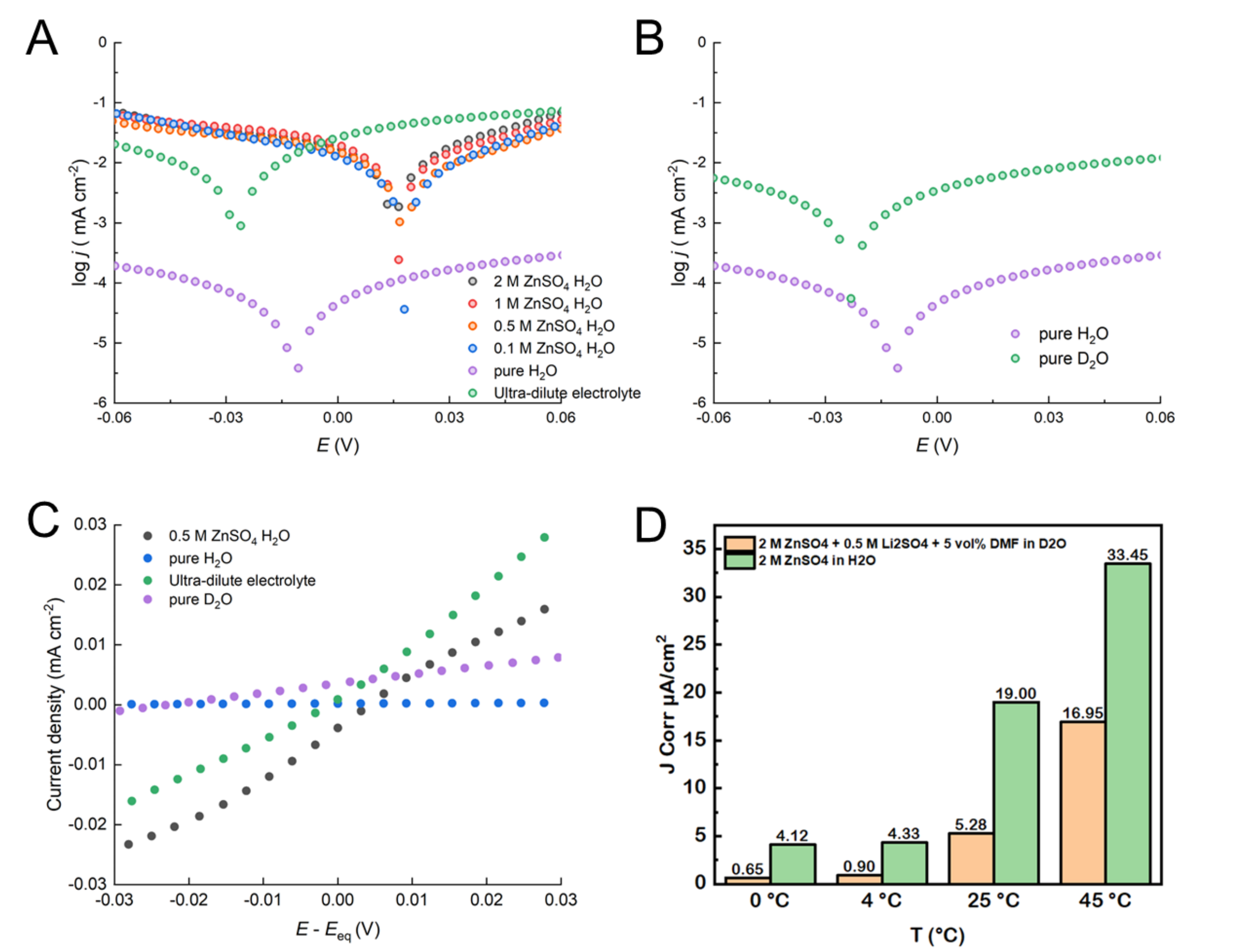

Supplementary Fig. 4. Tafel analysis. (A-B) Tafel plot of different electrolytes derived by LSV measurements. (C) Representative LSV data. (D) Corrosion current density from Tafel analysis at different temperatures.

To further confirm the solvation structure regulation and the hydrogen bonding behavior at different temperatures, we conducted Tafel analyses at 0, 4, 25, and 45 °C to compare 2 M $ZnSO_4$ in H_2O and 2 M $ZnSO_4$ + 0.5 M Li_2SO_4 + 5 vol% DMF in D_2O (Supplementary Fig. 4D). The electrolyte with additive and solvent modifications exhibits a lower corrosion current density across different temperatures, highlighting DMF-assisted SEI formation with solvation modulation and reduced HER kinetics in D_2O (kinetic isotope effect) that protects Zn during calendar aging.

Page S23:

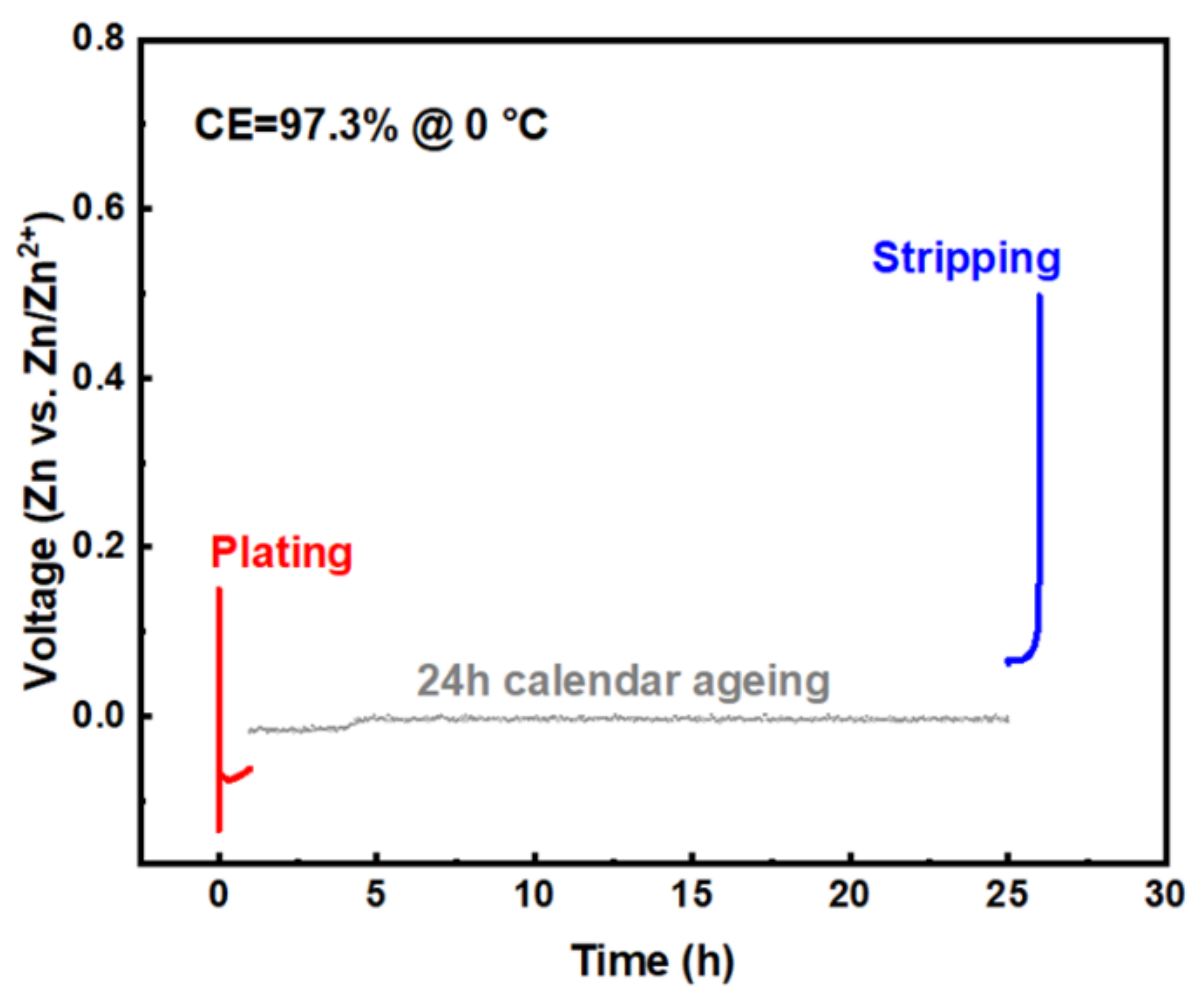

Supplementary Fig. 18. Voltage profile and capacity retention at 0 °C.

3. Undoubtedly, higher concentrations of metal salts are beneficial to enhance the ionic conductivity and ion mobility number of the system. Very low concentration of modified electrolyte system can slow down the negative electrode corrosion by reducing the hydrated H2O in the dissolved sheath, but it may also lead to a decrease in the number and kinetic performance of the transported Zn ions (as shown in Fig. 2F). The authors should address this issue by giving a basis for screening appropriate concentrations.

Authors' Response: We thank the reviewer for raising this key point regarding the trade-off between calendar-aging suppression with dilute Zn-salt and ion transport at higher salt concentrations. To provide an experimental basis for concentration screening, we measured EIS for 0.01 M $ZnSO_4$ in H_2O (Fig. R9). The Nyquist plot shows an extremely large impedance extending to ~3.6 kΩ, indicating severely limited ionic transport and sluggish interfacial kinetics. This directly shows that ultra-dilution beyond a certain point can degrade deposition/stripping performance. By contrast, 0.1 M $ZnSO_4$ represents the *lowest practical Zn-salt regime* in which the "ultra-dilute" concept is retained without inducing transport-dominated failure. The electrolyte resistance R_e is ~37.86 Ω for 0.1 M $ZnSO_4$ in H_2O, which is further reduced to ~8.10 Ω upon adding 0.5 M Li_2SO_4 as a supporting salt (Supplementary Fig. 7). Our optimized electrolyte (0.1 M $ZnSO_4$ + 0.5 M Li_2SO_4 + 5 vol% DMF in D_2O) maintains a practical R_e ~15.70 Ω, while exhibiting substantially improved interfacial stability during rest (Supplementary Fig. 7).

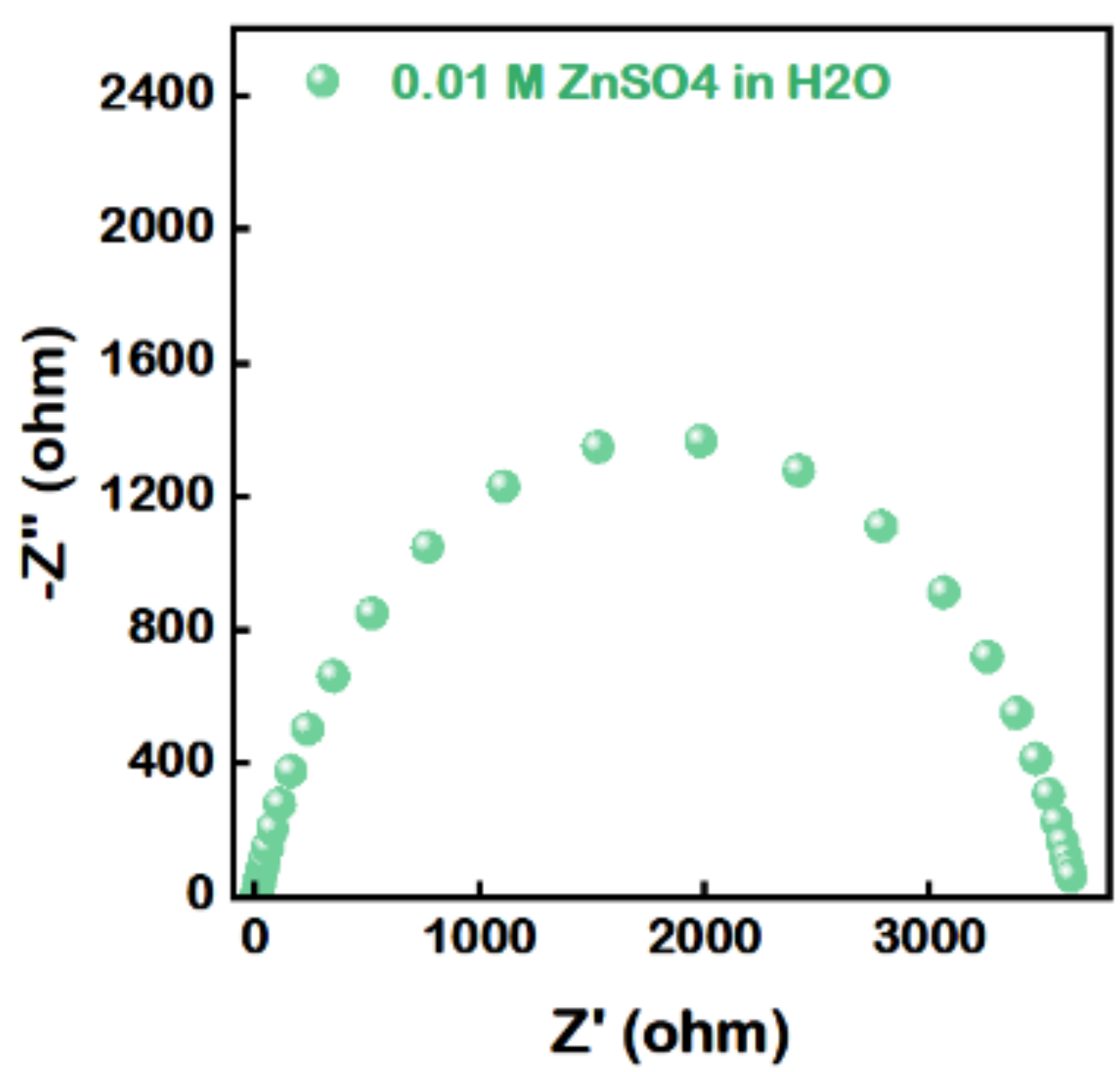

Fig. R9 Nyquist plot in 0.01 M ZnSO4 in H2O

Manuscript edits:

Page 10-11:

As shown in Supplementary Fig. 7, adding Li_2SO_4 substantially decreases the overall ionic transport resistance, offsetting the drawbacks of using an ultra-dilute Zn^{2+} without notably altering the reactivity of solvated water in the electrolyte.

Page 22-23:

Ionic conductivity (σ) was measured using a symmetric stainless-steel blocking-electrode configuration (SS|electrolyte|SS) assembled in CR2032 coin cells. A donut-shaped Celgard 2325 separator, saturated with electrolyte, was sandwiched between two stainless steel electrodes. The bulk ionic resistance (R_b) was extracted from the high-frequency intercept of the Nyquist plot obtained via EIS. Ionic conductivity was calculated as

$$\sigma = \frac{L}{R_b \times A}$$

Where L is the compressed separator thickness and A is the cross-sectional area defined by the separator geometry. While the absolute conductivity values are subject to uncertainty arising from this geometric approximation, all measurements were performed under identical conditions, ensuring internally consistent comparisons across the electrolyte formulations.

Page S12:

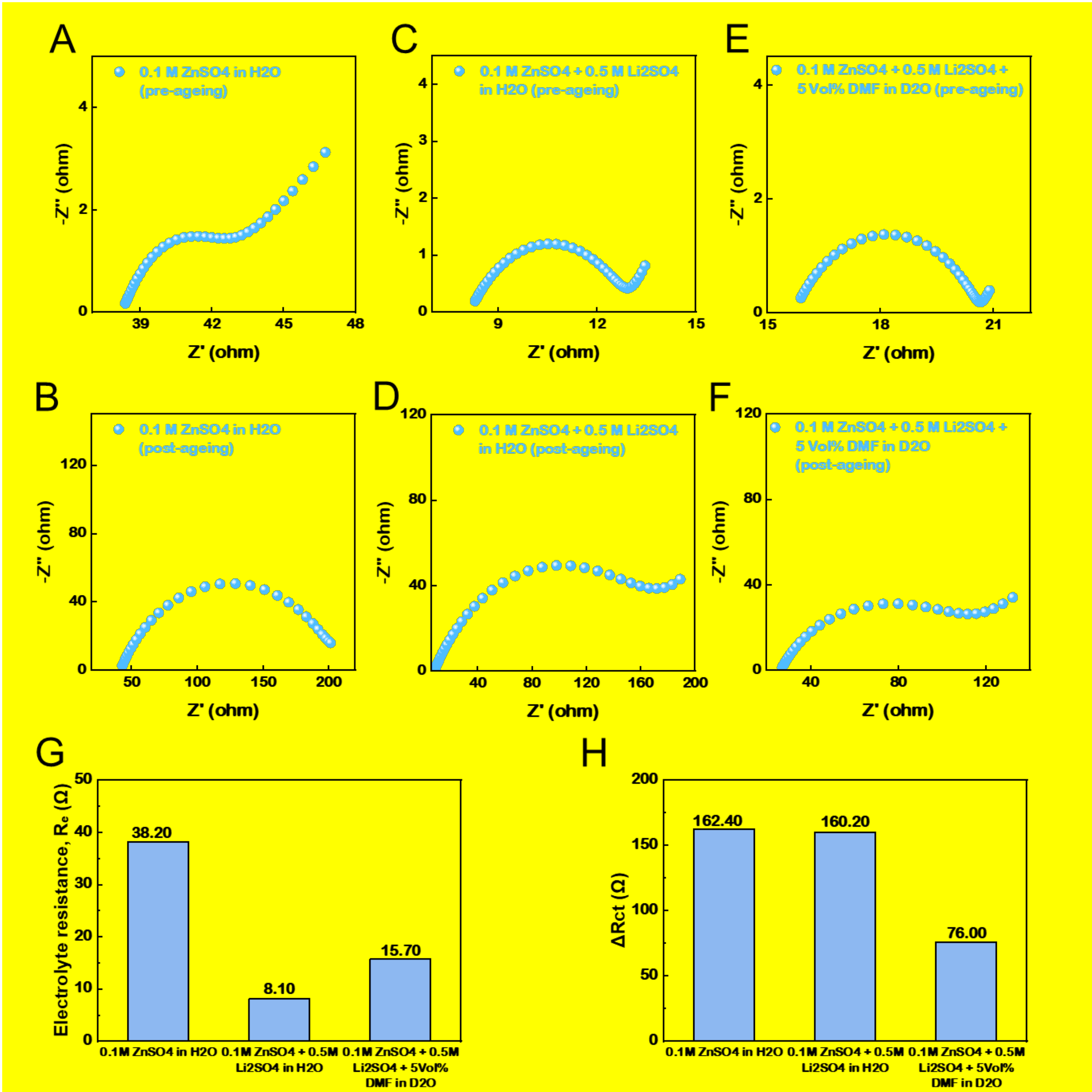

Supplementary Fig. 7. Cation concentration effects on electrolyte ionic conductivity. EIS profile before and after aging in (A-B) 0.1 M $ZnSO_4$ in H2O, (C-D) 0.1 M $ZnSO_4$ + 0.5 M Li_2SO_4 in H_2O, and (E-F) 0.1 M $ZnSO_4$ + 0.5 M Li_2SO_4 + 5 vol.% DMF in D_2O with equivalent circuit for fitting inset in (A). Summarized (G) electrolyte resistance and (H) aging induced charge transfer resistance growth.

4. The authors concluded that Li2SO4 could further reduce Zn ion transport resistance through solvent sheath modulation, and notably the addition of a high concentration of heterogeneous

cationic support salts was shown to construct a high-quality SEI layer and avoid dead Zn formation through, for example, electrostatic interactions, in addition to modulating solvation sheath behaviour (10.1038/s41893-025-01646-1; 10.1002/ adfm.202514585). The reviewers suggest further testing and analysis for possible differences in the mechanism of action of this work at very low addition concentrations compared to traditional high concentration double salts. Specifically, a variety of tests including zinc ion mobility and reaction activation energies for different systems would help to elucidate the mechanism of modification.

<u>Authors' Response:</u> We appreciate the reviewer's suggestion to distinguish the role of Li_2SO_4 under ultra-dilute conditions from the "dual-salt/high-concentration" electrolyte literature *[Nat. Sustain. 2025, 8, 11, 1349-1359; Adv. Funct. Mater. 2026, 36, 2, e14585]*, where heterogeneous cations can additionally regulate solvation structure at the interface beyond bulk transport. As suggested by the reviewer, to address this point quantitatively, we performed temperature-dependent EIS (0, 4, 25, 45 °C) for four electrolytes: (i) 0.1 M $ZnSO_4$ in H_2O, (ii) 0.1 M $ZnSO_4$+ 0.5 M Li_2SO_4 in H_2O, (iii) 2 M $ZnSO_4$ in H_2O, and (iv) 2 M $ZnSO_4$ + 0.5 M Li_2SO_4 in H_2O. From the Arrhenius temperature dependence of the extracted ionic resistance (Fig. R10A), we obtained the activation energy (Ea) for ion transport (Fig. R10B). The results show that in the dilute regime (0.1 M $ZnSO_4$), adding Li_2SO_4 decreases the ionic resistance at 25 °C from 2947 to 1467 Ω, while E_a remains essentially unchanged. This indicates that, under ultra-dilute $ZnSO_4$ conditions, Li_2SO_4 primarily improves transport by increasing charge-carrier availability rather than fundamentally changing the effective transport barrier with solvation structure modulation. However, in the higher concentrated regime (2 M $ZnSO_4$), adding Li_2SO_4 reduces both the ionic resistance (from 632 to 442 Ω at 25 °C) and Ea (from 0.633 to 0.570 eV, ~10% decrease). The decreased Ea suggests that Li_2SO_4 lowers the effective transport barrier under concentrated conditions. This trend is consistent with prior dual-cation electrolyte studies *[Nat. Sustain. 2025, 8, 11, 1349-1359; Adv. Funct. Mater. 2026, 36, 2, e14585]*, which reports pronounced Li^+/Zn^{2+} competitive solvation and corresponding Zn^{2+} coordination-environment restructuring at high salt concentrations. Such solvation changes may influence the effective transport barrier.

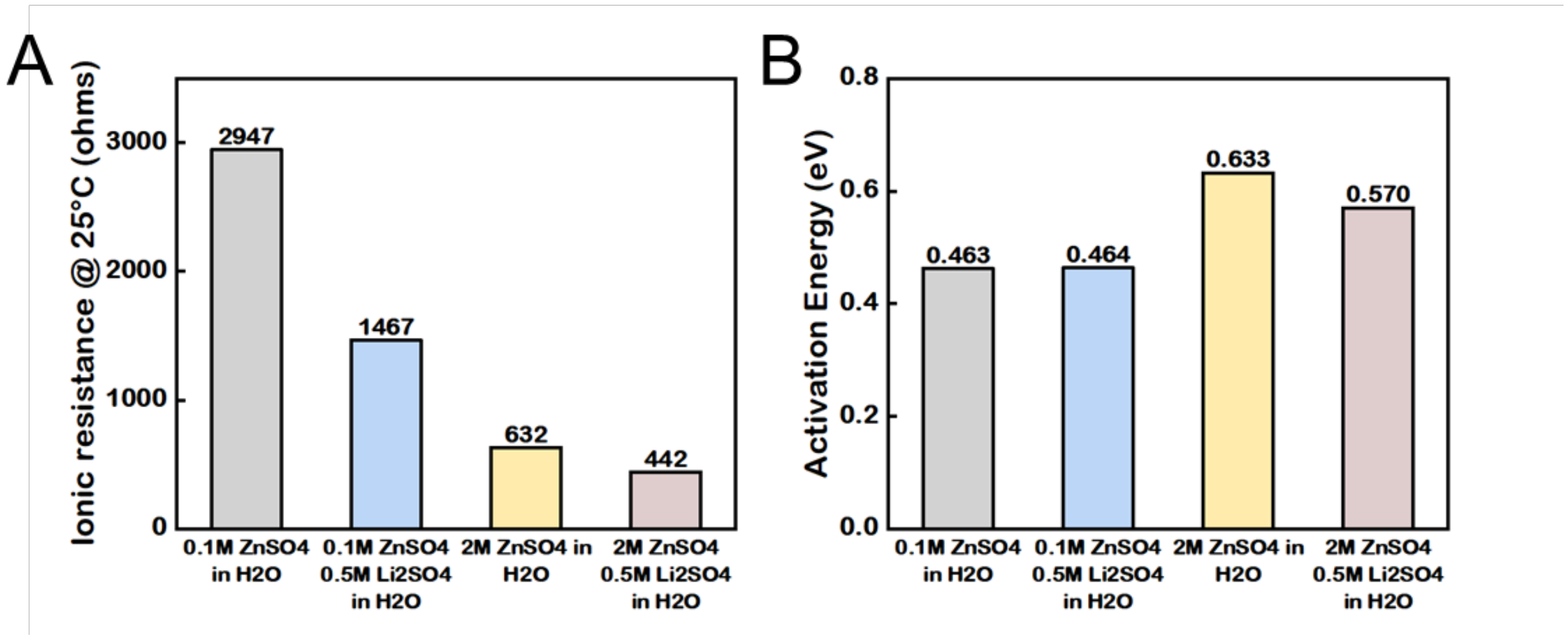

Fig. R10 (A) Ionic resistance at 25°C of the compared electrolyte; (B) Activation energy

Manuscript edits:

Page 22-23:

Ionic conductivity (σ) was measured using a symmetric stainless-steel blocking-electrode configuration (SS|electrolyte|SS) assembled in CR2032 coin cells. A donut-shaped Celgard 2325 separator, saturated with electrolyte, was sandwiched between two stainless steel electrodes. The bulk ionic resistance (R_b) was extracted from the high-frequency intercept of the Nyquist plot obtained via EIS. Ionic conductivity was calculated as

$$\sigma = \frac{L}{R_b \times A}$$

Where L is the compressed separator thickness and A is the cross-sectional area defined by the separator geometry. While the absolute conductivity values are subject to uncertainty arising from this geometric approximation, all measurements were performed under identical conditions, ensuring internally consistent comparisons across the electrolyte formulations.

5. The manuscript suggests that an ultra-low concentration electrolyte system depleted of solubilised water would be beneficial in preventing negative electrode corrosion, however this needs to be demonstrated more. Compositional distribution and morphological analysis of fully aged electrodes would be more effective in confirming this view. In addition, the reviewer believes that the ultra-low concentration electrolyte system may have a special effect on the transport and

de-embedding of zinc ions at the electrode-electrolyte interface, and suggests that further analysis of the interfacial behaviour should be carried out.

Authors' Response: We thank the reviewer for this constructive suggestion. In the revised manuscript, we therefore added (i) compositional distribution and morphological analysis of fully aged Zn electrodes via SEM, EDS, and TEM, and (ii) interfacial behavior analysis using EIS before/after calendar aging with equivalent-circuit fitting.

(i) Compositional distribution and morphological analysis of fully aged electrodes

As shown in Fig. R7A-B, E-F, post-rest morphology indicates that the morphological structure is stable and intact, with layers more orderly and closely packed. And the compositional distribution of elemental ratios (Fig. R7C-D, G-H) remains comparable (Zn:O:S ~ 1:1.8:0.2 before aging vs. 1:2.3:0.2 after aging), suggesting no ZHS growth as a dominant by-product. Together, these results support the idea that our optimized electrolyte reduces the driving force for aggressive corrosion during rest.

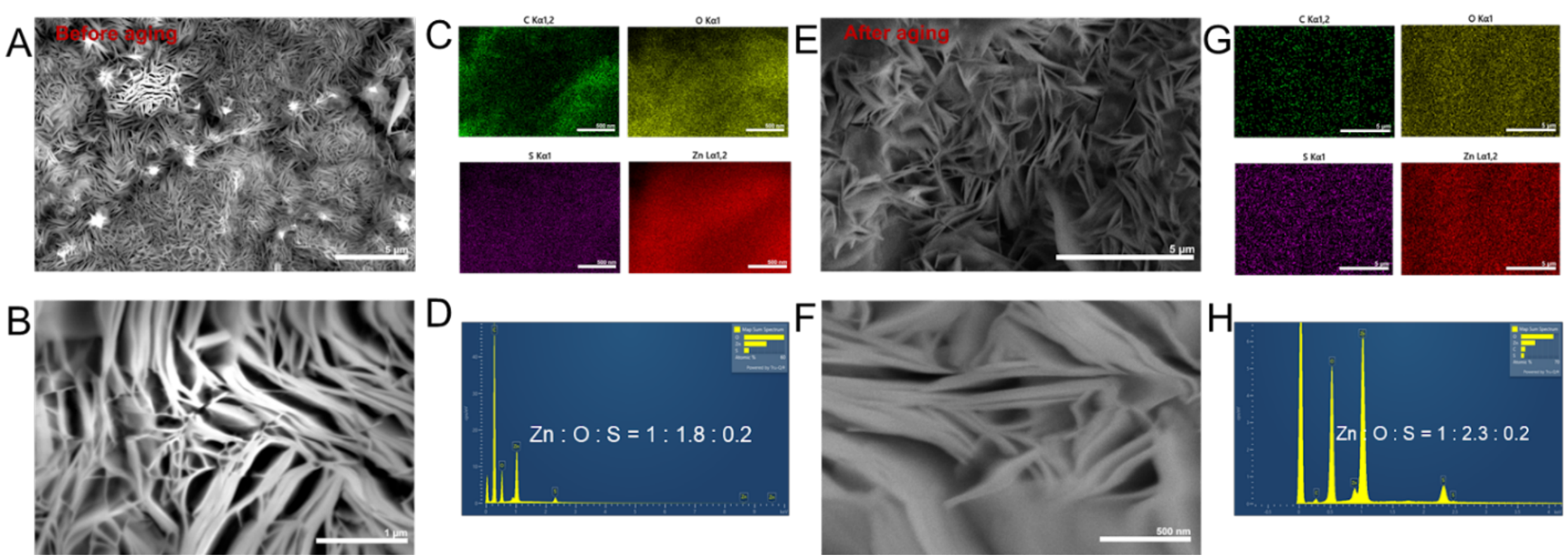

Fig. R6 SEM images of Zn deposits (A-B) before and (E-F) after aging, EDS mappings and quantitative elemental analysis (C-D) before and (G-H) after aging.

(ii) **Interfacial behavior analysis of Zn^{2+} transport at the electrode–electrolyte interface**

We agree that ultra-low Zn^{2+} concentration may influence interfacial Zn^{2+} transport and kinetics. To directly probe this, we added full Nyquist plots with equivalent-circuit fitting before/after 24 h aging (Supplementary Fig. 7A-F). Nyquist plots were fitted

with a standard equivalent circuit (Supplementary Fig. 7A), consistent with prior Zn battery EIS practice *[Nat Commun 14, 3067 (2023)]*. From the fitting, we quantified:

- **Electrolyte resistance R_e (high-frequency intercept)** as a transport-related metric (Supplementary Fig. 7G). Adding Li_2SO_4 to 0.1 M $ZnSO_4$ in H_2O reduces R_e from 37.86 Ω to 8.10 Ω (~79% decrease), providing direct quantitative evidence that Li_2SO_4 substantially decreases ionic transport resistance, consistent with our original qualitative statement. Our optimized electrolyte also shows a lower R_e (15.70 Ω) than 0.1 M $ZnSO_4$ in H_2O (37.86 Ω).
- **Aging-induced interfacial polarization**, quantified as ΔRct = Rct (24 h) – Rct (0 h) (Supplementary Fig. 7H). The baseline 0.1 M $ZnSO_4$ in H_2O shows a large ΔRct (163.5 Ω), while 0.1 M $ZnSO_4$ + 0.5 M Li_2SO_4 in H_2O remains similarly large (160.2 Ω), indicating that improving bulk transport alone does not sufficiently stabilize the interface during storage. In contrast, our optimized electrolyte shows a much smaller ΔRct (76.0 Ω), suggesting an interphase that maintains more favorable charge-transfer kinetics during calendar aging.

Taken together, ultra-dilute Zn^{2+} reduces corrosion propensity during calendar aging, but should be paired with interphase engineering. In our system, Li_2SO_4 primarily improves bulk transport R_e, whereas DMF-enabled interphase formation and D_2O solvent environment primarily suppresses aging-induced interfacial degradation ΔRct.

Manuscript edits:

Page 10-11:

As shown in Supplementary Fig. 7, adding Li_2SO_4 substantially decreases the overall ionic transport resistance, offsetting the drawbacks of using an ultra-dilute Zn^{2+} without notably altering the reactivity of solvated water in the electrolyte.

Page 22-23:

Ionic conductivity (σ) was measured using a symmetric stainless-steel blocking-electrode configuration (SS|electrolyte|SS) assembled in CR2032 coin cells. A donut-shaped Celgard 2325 separator, saturated with electrolyte, was sandwiched between two stainless steel electrodes. The

bulk ionic resistance (R_b) was extracted from the high-frequency intercept of the Nyquist plot obtained via EIS. Ionic conductivity was calculated as

$$\sigma = \frac{L}{R_b \times A}$$

Where L is the compressed separator thickness and A is the cross-sectional area defined by the separator geometry. While the absolute conductivity values are subject to uncertainty arising from this geometric approximation, all measurements were performed under identical conditions, ensuring internally consistent comparisons across the electrolyte formulations.

Page S12:

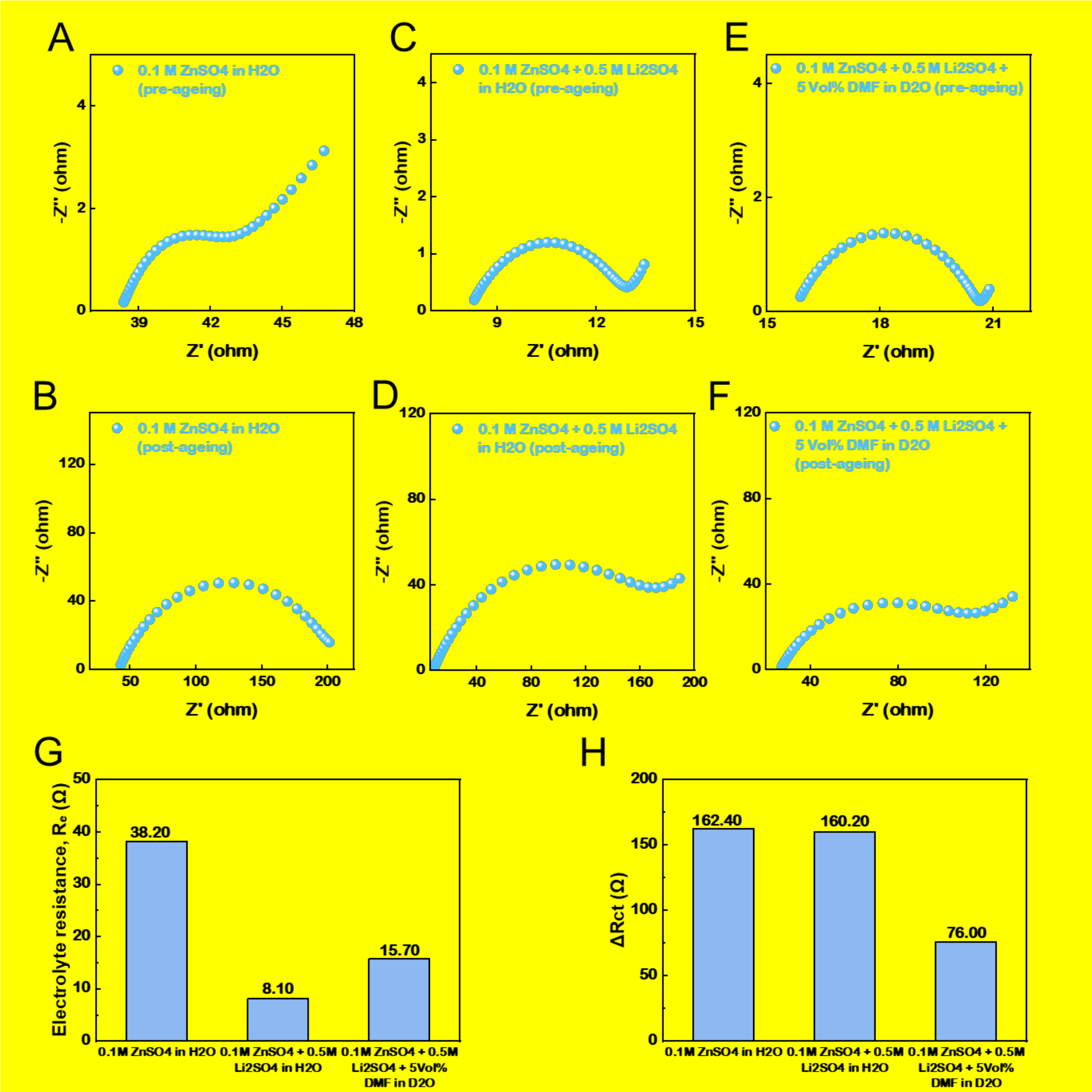

Supplementary Fig. 7. Cation concentration effects on electrolyte ionic conductivity. EIS profile before and after aging in (A-B) 0.1 M $ZnSO_4$ in H2O, (C-D) 0.1 M $ZnSO_4$ + 0.5 M Li_2SO_4 in H_2O, and (E-F) 0.1 M $ZnSO_4$ + 0.5 M Li_2SO_4 + 5 vol.% DMF in D_2O with equivalent circuit for fitting inset in (A). Summarized (G) electrolyte resistance and (H) aging induced charge transfer resistance growth.

6. "This nanostructured SEI, rich in … Zn deposition by minimizing uncontrolled side reactions." Are there any specific data, such as ion transference numbers or diffusion coefficients, that demonstrate the SEI film actively promotes Zn2+ migration?

Authors' Response: We thank the reviewer for the important question. While directly extracting a rigorous Zn^{2+} single-ion transference number or Zn^{2+} diffusion coefficient in our multi-ionic electrolytes requires specialized methods, we performed quantitative transport measurements and report $t_{+,app}$ as a consistent comparative descriptor (See Methods). We found a ~2× increase in the cationic transport fraction after introducing DMF (0.18 for 0.1 M $ZnSO_4$ + 0.5 M Li_2SO_4 in D_2O vs. 0.32 for 0.1 M $ZnSO_4$ + 0.5 M Li_2SO_4 + 5 vol% DMF in D_2O, Supplementary Fig. 8), supporting our statement that the DMF-derived SEI helps maintain reversible Zn deposition. We also revised the manuscript to claim more precisely as "cationic transport" rather than "Zn ion transport".

Manuscript edits:

Page 10-11:

As shown in Supplementary Fig. 7, adding Li_2SO_4 substantially decreases the overall ionic transport resistance, offsetting the drawbacks of using an ultra-dilute Zn^{2+} without notably altering the reactivity of solvated water in the electrolyte.

Page 22:

The apparent transference number (t^{+}_{app}) was tested in Zn||Zn symmetric cells using the protocol originally developed by Bruce and Vincent and widely used as a comparative transport metric. Briefly, cells were rested for 12 h, then polarized at ΔV = 10 mV for 1 h to reach steady state. Then, EIS was collected before/after polarization to obtain R_0 and R_{ss}, and t^{+}_{app} was calculated following the Bruce-Vincent framework:

$$t_{+}=\frac{I_{ss}(\Delta V-I_0R_0)}{I_0(\Delta V-I_{ss}R_{ss})}$$

Page S13:

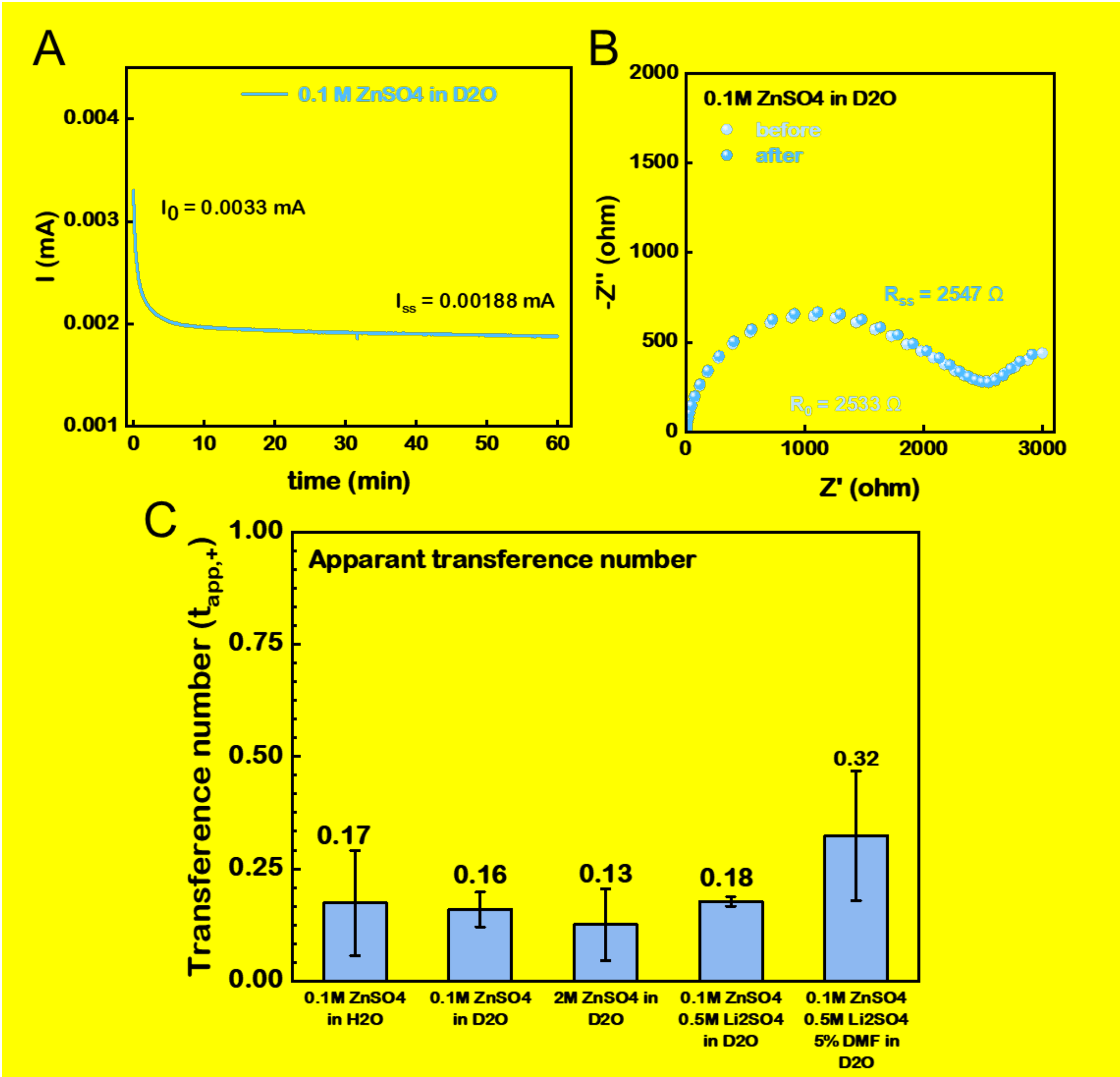

Supplementary Fig. 8. (A-B) Bruce Vincent method and (C) apparent transference number.

The manuscript addresses a timely and practically significant problem in aqueous zinc ion battery research—the severe capacity loss of metal anodes during calendar aging. The authors propose an unconventional "ultra-dilute" electrolyte strategy (0.1 M $ZnSO_4$ + Li_2SO_4 + DMF in D_2O) as an alternative to the widely adopted high-concentration "water-in-salt" concept, claiming that corrosion is mainly driven by solvated rather than free water molecules. The study combines density-functional analysis, spectroscopy, and electrochemical testing and reports markedly improved capacity retention and cycling stability.

While the topic is relevant and potentially influential, the current work does not yet meet the standard expected for publication in *Nature Communications*. The central mechanistic claim—that solvated water dominates corrosion—is not experimentally verified, the stability of the complex electrolyte are insufficiently characterized. The study's conceptual framework is appealing but currently more correlative than causal. Before acceptance, the authors should address the additional validation and mechanistic clarifications outlined below.

1. Mechanistic basis: "solvated water drives corrosion" lacks direct proof

The central hypothesis that solvated rather than free water molecules govern Zn corrosion is the conceptual foundation of the paper, yet it is supported solely by static DFT/COHP calculations and correlative electrochemistry. Actually, no experimental discrimination between solvated and free water species is provided. Without spectroscopic validation (e.g., operando Raman, ^{17}O NMR, isotopic exchange kinetics, or neutron scattering), the claim remains speculative.

The assertion that "free water is not reactive" is overstated. Even if free water has higher HER overpotential, the corrosion process in Zn involves mixed chemical-electrochemical steps ($Zn + 2H_2O \rightarrow Zn(OH)_2 + H_2$), in which both solvated and bulk species participate.

The authors should consider about complementing the DFT results with one characterization (for example, Raman or FTIR) to track coordinated vs. free-water vibrational modes under potential control.

2. Validation of electrolyte composition and stability

The multi-component electrolyte ($ZnSO_4$ + Li_2SO_4 + DMF + D_2O) introduces several reactive species. Stability of each under operating conditions is not demonstrated. DMF is known to decompose reductively on Zn and Cu surfaces, forming amides, formates, and carbonate species. The hypothesized reaction leading to $ZnCO_3$ + ZnO lacks evidence.

Li_2SO_4 can alter ionic transport but may also precipitate as Li_2CO_3 or $Li_2Zn(SO_4)_2 \cdot xH_2O$ during extended cycling. In the manuscript, no post-cycling XRD or ICP-OES analysis of electrolyte residues is shown.

So, I suggest the authors consider including more technology (such as XPS, ToF-SIMS, or ICP) to support the conclusion.

3. Electrochemical testing details should be provided

First, the aging protocol ("24 h rest after plating") lacks procedural details: OCV conditions, temperature control, and self-discharge should be monitored. Besides, electrolyte volume-to-electrode-area ratio is critical for ultra-dilute systems; it must be reported.

4. Absence of impedance and transport analysis

The claim that Li_2SO_4 addition "substantially decreases ionic transport resistance" is only qualitatively described. So, the authors should provide full Nyquist plots and fitted equivalent-circuit parameters before and after aging; Zn^{2+} transference numbers and apparent diffusion coefficients from GITT or EIS to quantify transport effects of dilution and isotopic substitution.

In addition, discussion of how 0.1 M Zn^{2+} maintains dendrite-free deposition without severe concentration polarization—contradictory to classical analysis, should also be included.

5. Terminology and claims

"Ultra-dilute" is an overstatement for 0.1 M $ZnSO_4$; typical aqueous Zn batteries operate in the 0.5–3 M range, so "low-concentration regime" would be more accurate.

Besides, the concluding statement that the design is "broadly applicable to Fe metal and Na-ion batteries" is speculative and should be deleted unless supporting data exist.

6. Besides, there are also some small problems:

(1)For example, The authors should clarify transport-limiting regime: Since ion conductivity is a critical trade-off in ultra-dilute systems, a brief discussion or table comparing the measured ionic conductivities of various electrolyte formulations (possibly from Supplementary Figure 3) would help contextualize the practical feasibility without new experiments.

(2)Add reference to broader aqueous systems: As mentioned above, there is no experimental evidence about the Na- or Fe-based batteries in the manuscript. If the authors want to prove the conceptual generality, they should consider mentioning analogous solvation-corrosion relationships in Na- or Fe-based aqueous chemistries in the Discussion (combined experimental results would be better). This would underscore the broad applicability of the 'solvated-water reactivity' principle.

(3)Optional visualization suggestion: A schematic summarizing how concentration, isotopic substitution, and additive choice synergistically suppress solvated-water activity could be added to the main text or Supplementary Information for readers' clarity. No additional experiments are needed.
